# Personalized polygenic profiling based on the genetic architecture of lipid metabolism in the Russian population

**DOI:** 10.3389/fcvm.2026.1707598

**Published:** 2026-06-02

**Authors:** Aleksandra Mamchur, Maria Bruttan, Veronika Daniel, Daria Kashtanova, Elena Zelenova, Irina Dzhumaniiazova, Mikhail Ivanov, Lorena Matkava, Olga Blinova, Sergey Mitrofanov, Liliya Golubnikova, Naiana Kumar, Ekaterina Maralova, Andrey Shingaliev, Marat Ezhov, Aleksey Meshkov, Uliana Chubykina, Olga Pogorelova, Mariia Tripoten, Tatyana Balahonova, Alexandra Viskova, Madina Komarova, Timur Gurtsiev, Natalia Gomyranova, Yulia Vorobeva, Anastasia Hotuleva, Maria Kolyaskina, Vladimir Yudin, Valentin Makarov, Anton Keskinov, Lyudmila Kuzmina, Sergey Boytsov, Sergey Yudin, Veronika Skvortsova

**Affiliations:** 1 Federal State Budgetary Institution «Centre for Strategic Planning and Management of Biomedical Health Risks» of the Federal Medical and Biological Agency (Centre for Strategic Planning, of the Federal Medical and Biological Agency), Moscow, Russia; 2 Federal State Budgetary Institution «National Medical Research Center for Cardiology Named After Academician Yevgeniy Chazov» of the Ministry of Health of the Russian Federation, Moscow, Russia; 3 Federal State Budgetary Institution «National Medical Research Center for Therapy and Preventive Medicine» of the Ministry of Healthсare of the Russian Federation, Moscow, Russia; 4 Federal State Budgetary Scientific Institution «Izmerov Research Institute of Occupational Health», Moscow, Russia; 5 The Federal Medical Biological Agency (FMBA of Russia), Moscow, Russia

**Keywords:** cholesterol, dyslipidemia, GWAS, HDL, LDL, polygenic score (PGS)

## Abstract

**Objective:**

Elevated cholesterol levels are associated with the risk of the most socially significant cardiovascular diseases, such as atherosclerosis, ischemic heart disease, and myocardial infarction.

**Methods:**

In this study, we sought to study the genetic architecture of lipid metabolism by conducting genome-wide association studies of total cholesterol, LDL-C, and HDL-C levels in a sample of the Russian population (*n* = 8,732) who were not carriers of variants linked to familial hypercholesterolemia and did not take lipid-regulating agents. Based on the detected associations and machine learning methods, several polygenic score models were constructed for each lipid type, and the link between polygenic scores and atherosclerosis, ischemic heart disease, and myocardial infarction was examined in an additional sample of patients diagnosed with either of these diseases (*n* = 3,954).

**Results:**

A meta-analysis of the results of the conducted genome-wide association studies showed that total cholesterol and LDL-C were largely associated with the same variants located in the *HMGCR, CERT1, POLK, ANKDD1B, APOC3, BCL3, CBLC, BCAM, NECTIN2, TOMM40, APOE, APOC1, APOC4,* and *SMARCA4* genes, while HDL-C was associated with variants located in the *LPL, ALDH1A2, LIPC,* and *CETP* genes. Men and women differed in genetic predictors of lipid levels, with variants in the *SMARCA4* and *LDLR* genes associated with cholesterol levels only in women.

**Conclusion:**

The models developed in this study consider age and a modifiable factor, BMI. Therefore, the personalized polygenic profiling approach presented in this study enables life-long CVD risk assessment.

## 1 Introduction

Elevated levels of total cholesterol or low-density lipoprotein cholesterol (LDL-C) and diminished high-density lipoprotein cholesterol (HDL-C) are key indicators of hypercholesterolemia. These indicators are linked to an increased risk of cardiovascular diseases (CVDs), which represent the leading causes of mortality worldwide. Consequently, effective management of hypercholesterolemia is imperative for mitigating the risk of CVDs, such as atherosclerosis (1) and its clinical manifestations, including ischemic heart disease (IHD) (2, 3).

The integration of genetic testing for monogenic dyslipidemia, or familial hypercholesterolemia (FH), into clinical practice has proven successful. FH ranks among the most common genetic disorders, with an estimated prevalence of one in 313 individuals globally (4). However, lipid levels in the majority of individuals are simultaneously influenced by multiple gene variants dispersed throughout their genomes. While these gene variants do not significantly impair lipid metabolism individually, their combined effect can contribute to polygenic dyslipidemia (PD). Polygenic inheritance may be influenced by lifestyle choices and can be addressed by managing modifiable risk factors. Assessing an individual's polygenic risk for dyslipidemia and related diseases could facilitate more effective healthcare, resulting in more personalized strategies for diagnosis, management, and prevention of this disease.

In 2021, Wu et al. presented a polygenic risk score model predicting LDL-C levels based on UK Biobank data from 377,286 White British individuals with relatively high accuracy (*r*^2^ = 0.215 on the validation sample) (5). Nomura et al. detected differences in predicted LDL-C levels between individuals with PD and FH (6). However, most existing polygenic risk score models do not differentiate between PD and FH. Failing to identify and account for differences in the underlying mechanisms of these two forms of dyslipidemia may not yield reliable results, especially when assessing polygenic risk.

Furthermore, a significant limitation of many studies is their reliance on open data sources that comprise results from different laboratories using varying protocols. These datasets are predominantly generated through whole-exome sequencing (WES), which fails to capture potentially significant variants located in regulatory genomic regions. Belkadi et al. have highlighted the advantages of whole-genome sequencing (WGS) over WES in identifying exome variants, with WGS yielding fewer false positive results (7).

It is also important to recognize that the ethnicity sensitivity of polygenic risk score models may vary (8). Hence, it is essential to develop population-specific models that are adjusted for ethnicity. Studies on the genetic architecture of lipid metabolism have been conducted across various ethnic groups, including Czech (9), Japanese (10), Chinese (11), and African-American populations (12), among others. In their meta-analysis, Graham et al. demonstrated that ethnic diversity can affect the outcomes of genome-wide association studies of lipid levels (13).

In light of the challenges outlined, our objective in this study was to examine the genetic architecture of PD within a comprehensive sample of the Russian population through WGS and identify variants affecting total cholesterol, LDL-C, and HDL-C levels. Leveraging the obtained data, we developed polygenic score models predicting changes in lipid levels depending on an individual's age, BMI, and genetic profile. Additionally, we analyzed the link between the polygenic scores and the risk of CVDs. The tools presented in this study could facilitate a timely prediction of changes in lipid levels across one's lifespan and enable personalized strategies for the prevention of metabolic disorders.

## 2 Methods

### 2.1 General population sample

Data for the general population study (*n* = 8,732 participants) were obtained from the database of the Centre for Strategic Planning and Management of Biomedical Health Risks of the Federal Medical and Biological Agency of Russia (14). Data from individuals undergoing lipid-lowering therapy and carrying pathogenic and likely pathogenic variants (PVs and LPVs) linked to FH were removed from the study. The assessment for PVs was carried out using the protocol provided in our previous study (15). The levels of total cholesterol and HDL-C in the biochemical blood analysis were measured using direct methods, while the concentration of LDL-C was calculated using the Friedewald formula.

The study was approved by the Ethics Committee of the FMBA's Center for Strategic Planning and Management of Biomedical Health Risks (excerpt from Protocol No. 5, dated December 28, 2020, and Protocol No. 2, dated June 1, 2021). All participants provided informed consent to participate in the study.

From the same population, 383 individuals were selected as the external validation set using an identical protocol.

### 2.2 Cardiac patients

Data for cardiac patients (*n* = 3,954 participants) were obtained from the database compiled as a result of an ‘omics’ technology-based study conducted by the National Medical Research Center of Cardiology, named after Academician E.I. Chazov (NMRCCAC), and the Center for Strategic Planning and Management of Biomedical Health Risks of the Federal Medical Biological Agency between 2021 and 2023 to examine molecular diagnostic markers for cardiovascular diseases (CVDs) in blood and serum samples of 4,856 inpatients admitted to the NMRCCAC and recruited on an all-comers basis (ClinicalTrials ID NCT06253481). The levels of total cholesterol and HDL-C in the biochemical blood analysis were measured using direct methods, while the concentration of LDL-C was calculated using the Friedewald formula.

Data from individuals carrying PVs and LPVs linked to FH were removed from further analysis. The assessment for PVs was carried out using the protocol provided in our previous study (15).

The study was approved by the ethics committee of the NMRCCAC (excerpt from Protocol No. 271, dated September 27, 2021). All patients provided informed consent to participate in the study.

### 2.3 Blood sampling and DNA sequencing

The QIAamp DNA Mini Kit (Qiagen, Germany) was used for DNA extraction from whole blood samples. The Nextera DNA Flex kit (Illumina, USA) was used for WGS library preparation. The NovaSeq 6000 S4 Reagent Kit (300 cycles) (Illumina, USA) was used to sequence samples to 150 bp reads.

Sequenced data in BCL format were demultiplexed using the bcl2fastq2 Conversion Software v2.20.0.422 (Illumina) (16) to obtain FASTQ files. The sequencing quality was checked using Sequencing Analysis Viewer v2.4.7 (Illumina) (17). The quality of FASTQ files was checked using FastQC v0.11.9 (18). Reads were aligned to the reference genome (GRCh38.d1.vd1) (19) using the Illumina DRAGEN Bio-IT Platform (v07.021.510.3.5.7) (20). The quality of alignment of BAM files was checked using DRAGEN, FastQC v0.11.9 (21), SAMtools v1.13 (22), and mosdepth v0.3.1 (23). All samples were checked for duplicates, unmapped reads, and other quality metrics. The mean sequencing coverage was 30x for all samples.

Strelka2 v2.9.10 (Illumina) (24) with default settings was used for small variant calling (of up to 50 b.p.). CrosscheckFingerprints (Picard) containing a haplotype map file (25) was used to check for duplicates.

The bioinformatics pipeline was validated using the HG001 reference genome from the Genome in a Bottle (GIAB) consortium (v.3.3.2) (F-score = 99.83%) (26).

### 2.4 Genome-wide association study

To adjust for ethnic diversity, the first 10 principal components from the principal component analysis (PCA) of the genomes were used as covariates.

Variants violating the Hardy-Weinberg equilibrium (*p*-value < 10^−6^); variants with an allele frequency (AF) of more than 0.98; multiallelic variants; and variants with a minor allele frequency (MAF) of less than 0.01 were removed.

Genome-wide associations were tested using the following linear regression equation:

$$Y=\beta_{0}+\beta_{c}\;\times \;C+\beta_{g}\;\times \;G,$$

where:

*β_0_* = constant,

*β_c_* = covariate effect vector,

*C* = covariate vector,

*β_g_* = genotype effect vector,

*G* = genotype vector.

Calculations were performed using statsmodels v0.12.2 (Python) and parallelized in Spark Cluster. Age, sex, and BMI were used as additional covariates. Variants with a *p*-value of less than 5,0 × 10^−8^ were considered significant. Results were visualized using LocusZoom, a Javascript library.

### 2.5 Gene significance assessment and gene networks

The significance of each gene was assessed as the cumulative significance of all its detected SNPs using the VEGAS2 algorithm (27). All obtained *p*-values were adjusted for multiple testing using the Benjamini–Hochberg procedure.

The interactions between proteins of key genes involved in cell metabolism were visualized as a gene interaction network utilizing the STRING database (28). A pair of genes was considered to be interacting if their respective proteins had been experimentally shown to interact. A search was conducted for gene strings of no more than three genes connecting the highest number of significant genes. The results were visualized using the pyvis library in Python v. 3.9.12.

### 2.6 Meta-analysis of GWAS results

The statistical analysis was conducted using Python version 3.9.12. To compare GWAS results, variants with a *p*-value < 10^−5^ in at least one GWAS were selected, and for each of the selected variants, the relationship between its *p*-values in two GWASs was examined. When analyzing the results of three GWASs concurrently, the variants were visualized as points in a UMAP space. Variant clusters were identified visually, indicating the names of genes whose variants were more frequently represented within each of the identified clusters.

### 2.7 Polygenic models

To construct polygenic score model, we selected polymorphisms that had a frequency of occurrence of over 2% in the training set and a *p*-value < 10^−6^. Before modeling, the sample was divided into training and test sets in a 4:1 ratio and stratified by the target variable. All polygenic models were constructed using two machine learning methods: linear regression (LR) and random forest regression (RF).

Feature selection for the logistic regression (LR)-based model was performed using ANOVA test results. The model was constructed using LinearRegression algorithm (Python v3.10, scikit-learn v1.6.1 library).

The RF-based predictive models were constructed using RandomForestRegressor (scikit-learn v1.2.1) in Python (v3.8) (29). The optimal number of decision trees, providing the highest rate of explained variance, was estimated within the range of 500 to 3,000 trees spaced evenly at intervals of 100. The optimization included hyperparameter tuning and 10-fold cross-validation.

Sex (excluding sex-specific models), age, BMI, and the first 10 principal components from the principal component analysis of the data from the general population sample were used as covariates.

The final model was selected based on the highest coefficient of determination, *r*^2^, on the test set.

### 2.8 Polygenic models validation

External validation set of participants was used to perform independent validation of the created models. Moreover, we have performed a comparison between created models and previously published ones which were deposited in the Polygenic Score (PGS) Catalog database. For the traits “total cholesterol,” “HDL cholesterol,” and “LDL cholesterol,” lists of all available polygenic scores were compiled from PGS Catalog database. For participants in the external validation set, weighted sums of genetic components corresponding to these scales were calculated. These sums represent the product of the genotype effect vector and the genotype vector. The accuracy of predictions was assessed using partial correlation. A similar procedure was performed for the linear regression models derived in this study.

### 2.9 Assessment of associations between polygenic scores and CVDs

The models were used to estimate genetically predetermined cholesterol levels in both the general population sample and cardiac patients who had dyslipidemia, ischemic heart disease, myocardial infarction, or atherosclerosis as the primary or secondary diagnosis. The comparison of polygenic scores between these two participant groups was conducted using the Mann–Whitney test and logistic regression analysis. The odds ratio is reported as the exponentiated logistic regression coefficient.

### 2.10 Individual polygenic profiling

An individual polygenic profile was based on polygenic scores calculated for an individual using different combinations of variable covariates, such as BMI and age, with genetic parameters remaining constant. BMI was set within the 15 kg/m^2^–39 kg/m^2^ range (with a 3 kg/cm^2^ interval) and age within the 30–80-year range (with a ten-year interval). Polygenic scores for every BMI-age combination were visualized as a set of curves. Individual health risk was assessed based on the results of individual profiling, along with the established genetic associations between polygenic scores and CVDs.

## 3 Results

### 3.1 Key characteristics of the research sample

#### 3.1.1 General population sample

The general population sample comprised 8,732 individuals who were not undergoing treatment with lipid-regulating agents and had no predisposition to FH. The median age of the participants was 52 years; 59.5% of them were women. Table 1 presents the key characteristics of the general population sample. Genetic data from these individuals were used in genome-wide association studies.

**Table 1:** Key characteristics of the research sample.

Feature	Entire sample	Men	Women
General population sample			
Number of participants	8,732	3,540	5,192
Age, years	52.0 [46.0; 58.0]	51.0 [45.0; 57.0]	53.0 [47.0; 58.0]
BMI, kg/m^2^	27.1 [24.2; 30.3]	26.8 [24.5; 29.4]	27.3 [23.9; 31.2]
Total cholesterol, mmol/L	5.7 [5.3; 6.3]	5.5 [5.2; 5.9]	5.9 [5.4; 6.49]
LDL-C, mmol/L	3.69 [3.2; 4.2]	3.6 [3.1; 4.0]	3.7 [3.3; 4.3]
HDL-C, mmol/L	1.4 [1.2; 1.7]	1.3 [1.1; 1.5]	1.5 [1.3; 1.8]
External validation set			
Number of participants	383	159	224
Age, years	48.0 [37.5; 55.0]	45.0 [36.0; 54.0]	49.5 [38.8; 56.0]
BMI, kg/m^2^	27.1 [23.9; 30.6]	27.5 [24.6; 30.4]	26.9 [23.2; 30.7]
Total cholesterol, mmol/L	5.8 [5.4; 6.4]	5.75 [5.41; 6.30]	5.9 [5.4; 6.4]
LDL-C, mmol/L	4.06 [3.61; 4.52]	4.15 [3.66; 4.53]	4.0 [3.57; 4.49]
HDL-C, mmol/L	1.46 [1.25; 1.75]	1.29 [1.16; 1.56]	1.61 [1.37; 1.82]
CVD parients			
Number of participants	3,954	2,234	1,720
Age, age	66.0 [58.0; 74.0]	64.0 [56.3; 72.0]	68.0 [60.0; 75.0]
BMI, kg/m^2^	28.4 [25.3; 32.01]	28.4 [25.5; 31.5]	28.4 [24.9; 32.7]
Total cholesterol, mmol/L	4.2 [3.49; 5.16]	4.04 [3.4; 4.96]	4.47 [3.7; 5.4]
LDL-C, mmol/L	2.31 [1.7; 3.1]	2.23 [1.7; 3.02]	2.45 [1.8; 3.3]
HDL-C, mmol/L	1.17 [0.98; 1.42]	1.08 [0.92; 1.28]	1.32 [1.11, 1.55]
Lipid regulating agents	2,480 (70.1%)	1,441 (73.3%)	1,039 (66.1%)
Atherosclerosis	3,000 (75.9%)	1,818 (81.4%)	1,182 (68.7%)
Ischemic heart disease	2,163 (54.7%)	1,442 (64.5%)	999 (58.1%)
Myocardial infarction	1,060 (26.8%)	806 (36.1%)	254 (14.8%)

### 3.2 External validation set

383 participants from the same population as general population sample were selected to perform independent validation of polygenic models. Key characteristics of the external validation set are presented in Table 1. They are not undergoing treatment with lipid-regulating agents and had no predisposition to FH. Their genetic data were not used for GWAS.

### 3.3 CVD patients

A sample of 3,954 patients specifically recruited from patients admitted to the National Medical Research Center of Cardiology named after Academician E.I. Chazov to assess the association between polygenic scores and CVDs. These individuals underwent a comprehensive cardiovascular examination and were diagnosed with CVDs (Table 1). This sample did not include participants with FH.

### 3.4 Genetic architecture of lipid metabolism in the Russian population

#### 3.4.1 Genome-wide association studies of lipid metabolism in the general population sample

To examine the genetic basis of lipid metabolism, three separate genome-wide association studies (GWASs) were conducted, focusing on total cholesterol, LDL-C, or HDL-C levels.

**Figure 1:**
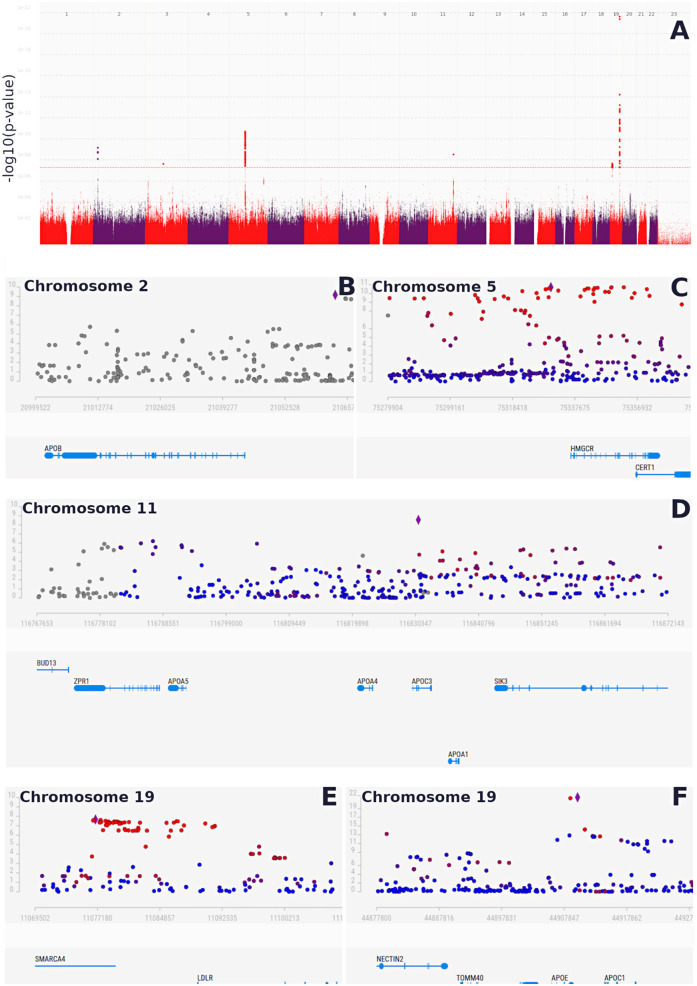
GWAS results for total cholesterol levels as a continuous variable. **(A)** Manhattan plot illustrating GWAS findings for total cholesterol levels in men. **(B–F)** LocusZoom of genomic regions containing genes with variants most significantly associated with total cholesterol levels.

There were 142 variants significant for total cholesterol in the *HMGCR, CERT1, POLK, ANKDD1B, APOC3, BCL3, CBLC, BCAM, NECTIN2, TOMM40, APOE, APOC1, APOC4,* and *SMARCA4* genes*,* as well as intergenic regions (Figure 1, Supplementary Table S1).

**Figure 2:**
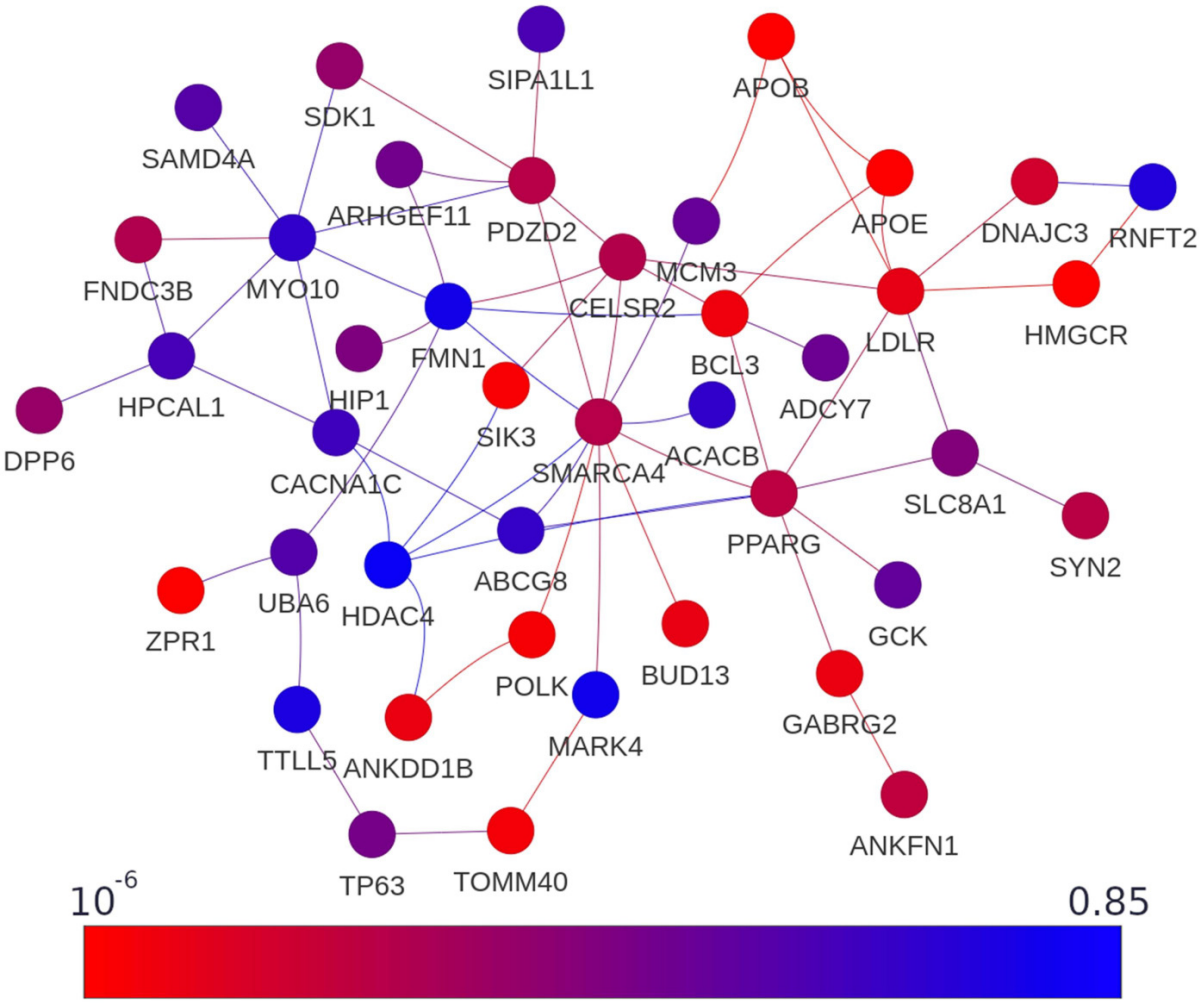
Network of genes significantly associated with total cholesterol levels. The colors reflect gene significance in the GWAS (*p*-values; see the color scale), calculated via the VEGAS2 algorithm.

Based on the cumulative significance of all variants per gene, the following genes demonstrated the strongest association with total cholesterol levels: *HMGCR* (*p*-value = 0.0008), *APOE* (*p*-value = 0.0008), *APOB* (*p*-value = 0.0008), *APOA1-AS* (*p*-value = 0.0008), *APOC1* (*p*-value = 0.0008), *APOA5* (*p*-value = 0.0008), *ZPR1* (*p*-value = 0.001), *POLK* (*p*-value = 0.002), *SIK3* (*p*-value = 0.003), *CERT1* (*p*-value = 0.003), *TOMM40* (*p*-value = 0.004), *BCL3* (*p*-value = 0.006), *GABRG2* (*p*-value = 0.006), *ANKDD1B* (*p*-value = 0.006), *NECTIN2* (*p*-value = 0.007), *SPDYE18* (*p*-value = 0.007), *LDLR* (*p*-value = 0.009), *PRKAA2* (*p*-value = 0.01), *APOC3* (*p*-value = 0.01), *APOA4* (*p*-value = 0.02), *APOA1* (*p*-value = 0.02). All *p*-values were adjusted for multiple testing using the Benjamini-Hochberg procedure (for false discovery rate). Figure 2 shows the protein-to-protein interaction network for the above genes.

**Figure 3:**
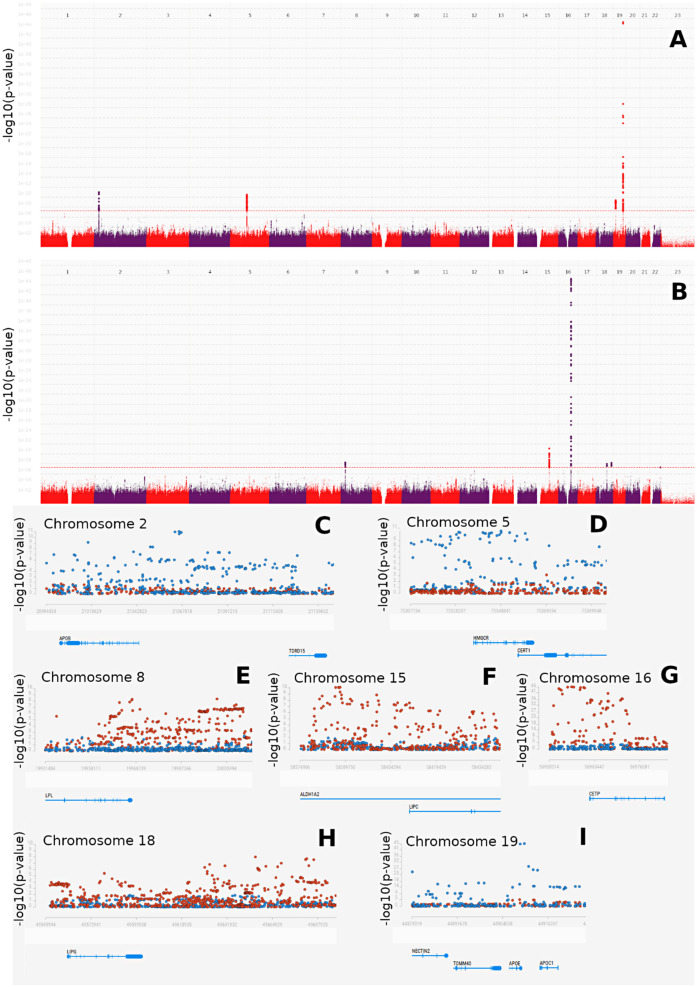
GWAS results for LDL-C and HDL-C levels. **(A)** Manhattan plot for GWAS results for LDL-C (blue dots). **(B)** Manhattan plot for GWAS results for HDL-C. **(C–I)** LocusZoom of genomic regions containing genes with variants most significantly associated with LDL-C (blue) and HDL-C (red).

There were 217 variants significant for LDL-C levels in the *APOB, ANKRD31, HMGCR, CERT1, POLK, ANKDD1B, CEACAM22D, BCL3, CBL3, BCAM, NECTIN2, TOMM40, APOE, APOC1, APOC1P1, APOC4, RELB, CLASRP, NKPD1, SMARCA4,* and *LDLR* genes*,* as well as intergenic regions (Figure 3A, Supplementary Table S2).

There were 104 variants significant for HDL-C levels in the *LPL, ALDH1A2, LIPC,* and *CETP* genes, as well as intergenic regions (Figure 3B, Supplementary Table S3).

The comparative analysis of the GWAS results revealed a substantial overlap between significant variants associated with total cholesterol and LDL-C levels. In contrast, variants significant for HDL-C levels were located in completely different regions of the genome, not associated with either total cholesterol or LDL-C levels (Figures 3С–I). Thus, the significance of variants for total cholesterol levels may be largely attributed to their significance for LDL-C levels. The *p*-values for most variants associated with total cholesterol and LDL-C were lower for total cholesterol. This may be accounted for by HDL-C representing 25% of total cholesterol within the sample, i.e., of the overall concentration of HDL-C, LDL-C, and VLDL-C.

### 3.5 Differences in total cholesterol predictors between men and women

Men were more predisposed to dyslipidemia and adverse outcomes associated with it. Our initial attempts to create polygenic scales for cholesterol levels revealed a significant influence of sex on the predictions, an effect that persisted in the final models. The notable difference in the risk of dyslipidemia between men and women suggests that the underlying molecular and genetic mechanisms of the examined phenotypes may also differ between the sexes. To explore this hypothesis, we conducted separate genome-wide association studies of total cholesterol levels for men and women.

**Figure 4:**
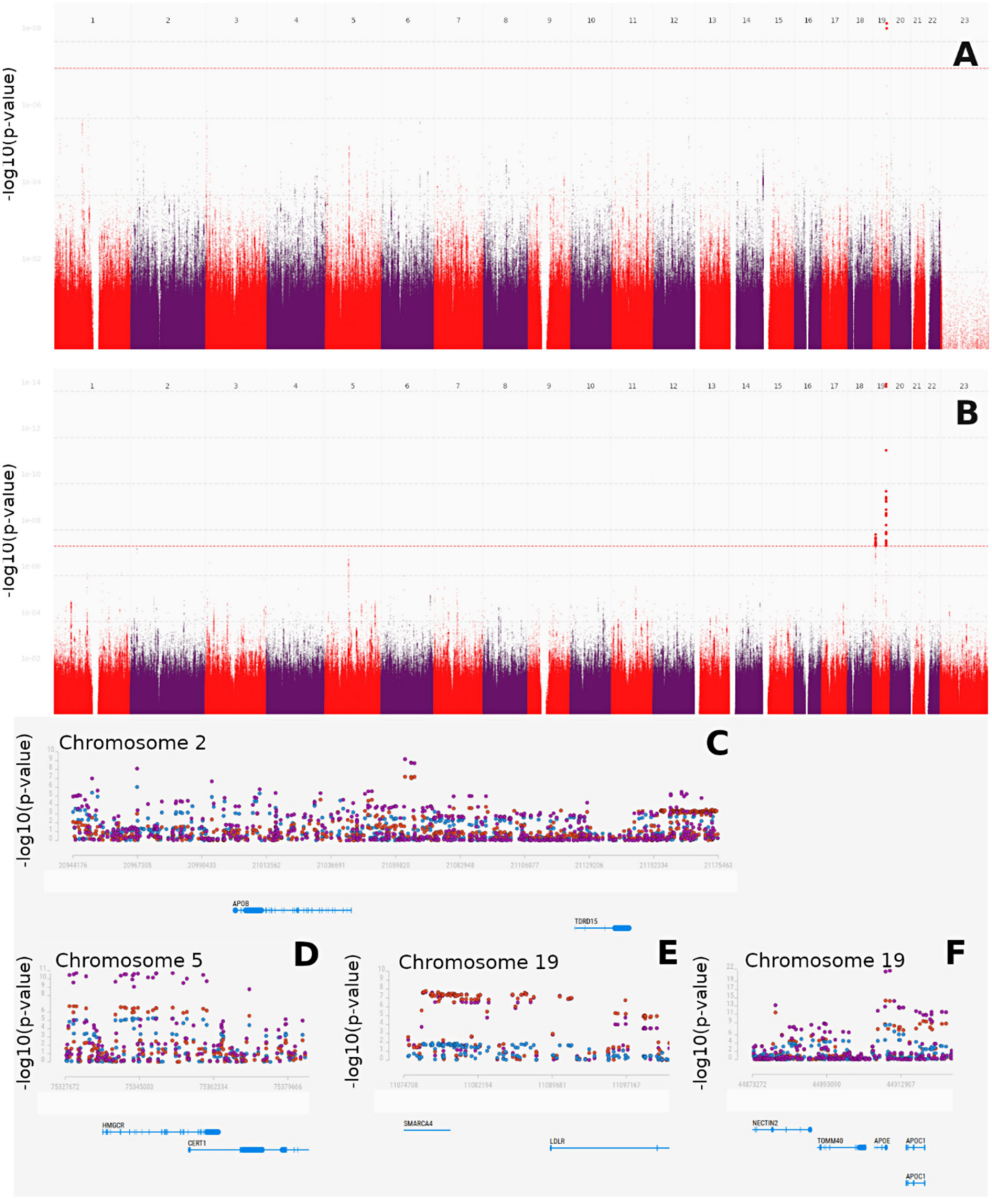
GWAS results for total cholesterol levels in men and women. **(A)** Manhattan plot for GWAS results for total cholesterol levels in men (blue dots). **(B)** Manhattan plot for GWAS results for total cholesterol levels in women (red dots). **(C–F)** LocusZoom of the genomic regions containing genes цшер variants most significantly associated with total cholesterol levels. The dots indicate GWAS results for total cholesterol levels in men (blue), women (red), and the general population sample (purple).

In men, only two variants on chromosome 19 met the genome-wide significance threshold: rs7412 (*APOE*; regression coefficient = −0.2369; *p*-value = 4.561 × 10^−9^) and rs1065853 (intergenic region; regression coefficient = −0.2384; *p*-value = 3.329 × 10^−9^) (Figure 4A). Both variants demonstrated protective effects, as indicated by their negative regression coefficients. In women, 51 significant variants were detected in *SMARCA4*, *BCAM*, *NECTIN2*, *APOE*, *APOC1*, and intergenic regions (Figure 4B, Supplementary Table S4). Most of these variants were also protective; however, several variants on chromosome 19, such as rs429358 and rs769449 in *APOE*, were associated with increased cholesterol levels.

All significant variants for both men and women were located on chromosome 19. However, both GWASs detected sub-significant variants on chromosome 2, near the *APOB* gene (Figure 4C), and on chromosome 5, near the *HMGCR* and *CERT1* genes (Figure 4D). These sub-significant variants were significant in the GWAS for the general population sample. However, their significance varied in descending order for the general population sample, women, and men (Figures 4C,D), which could be accounted for by the decreasing sample size of each subgroup. A similar observation was made for a site near the *APOE* gene on chromosome 19 (Figure 4F). However, at another site on chromosome 19 near the *SNARCA4* and *LDLR* genes, significant variants were detected only in the GWAS for the general population sample and the GWAS for women, whereas in the GWAS for men, this region did not contain variants with a *p*-value < 0.01, i.e., even sub-significant variants were not detected. All variants significant for the general population sample and women in this region have negative coefficients, i.e., are associated with lower levels of cholesterol.

**Figure 5:**
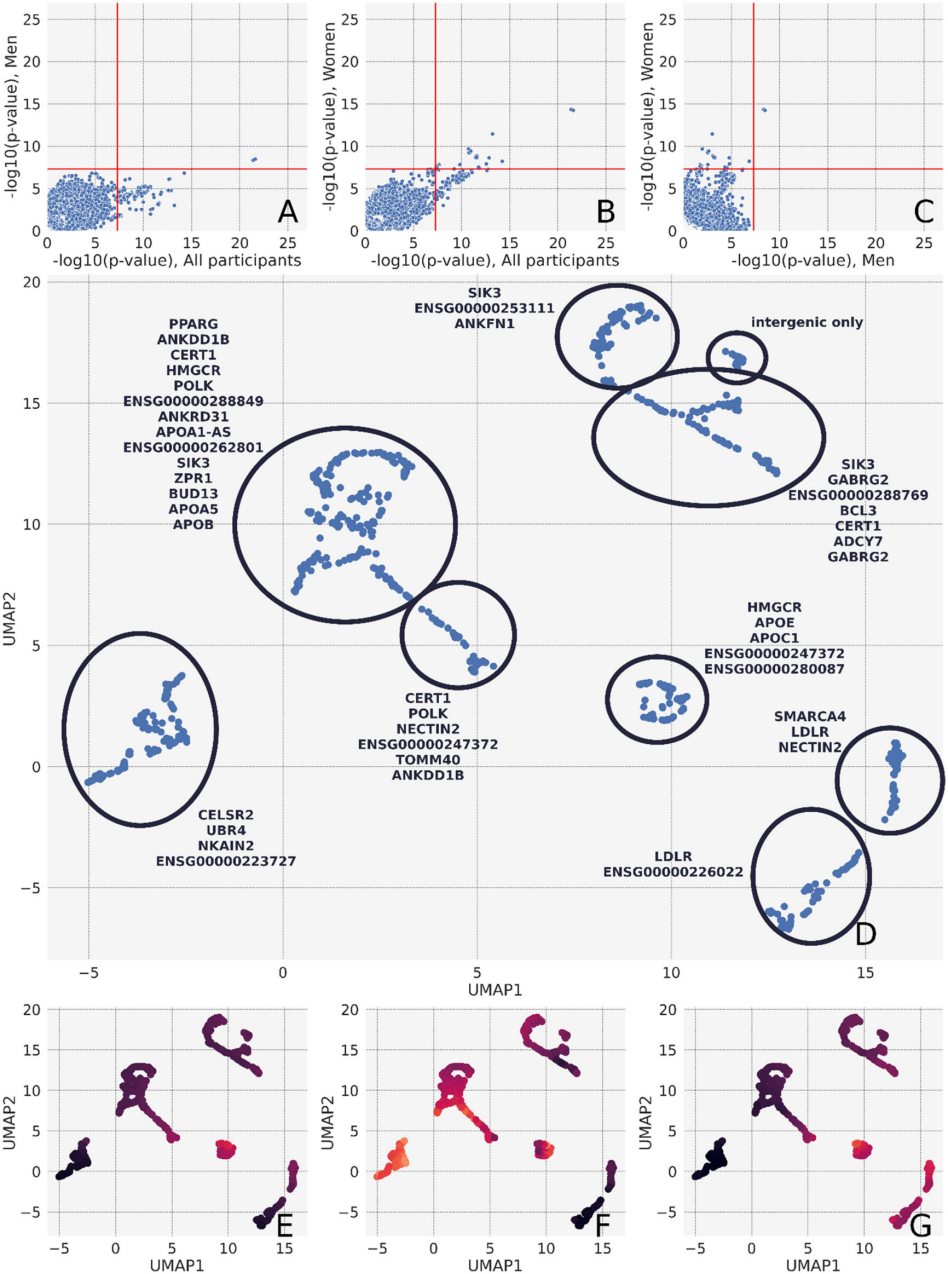
Meta-analysis of GWAS results for total cholesterol levels in men and women. **(A)** Comparison of GWAS results for total cholesterol levels in men and the general population sample. **(B)** Comparison of GWAS results for total cholesterol levels in men and women. **(C)** Comparison of GWAS results for total cholesterol levels in women and the general population sample. **(D)** Comparison of GWAS results for total cholesterol levels in men, women, and the general population sample in a UMAP space. **(E)** Comparison of GWAS results for total cholesterol levels in men, women, and the general population sample in a UMAP space. The color intensity indicates the significance [-log10(*p*-value)] of variants in the GWAS for total cholesterol in the general population sample, with lower significance indicated by darker colors and higher significance indicated by lighter colors. **(F)** Comparison of GWAS results for total cholesterol levels in men, women, and the general population sample in a UMAP space. The color intensity indicates the significance [-log10(*p*-value)] of variants in the GWAS for women, with lower significance indicated by darker colors and higher significance indicated by lighter colors. **(G)** Comparison of GWAS results for total cholesterol levels in men, women, and the general population sample in a UMAP space. The color intensity indicates the significance [-log10(*p*-value)] of variants in the GWAS for women, with lower significance indicated by darker colors and higher significance indicated by lighter colors.

We conducted a meta-analysis of the GWASs focusing on total cholesterol in the general population sample, in men, and in women (Figure 5). The results of all three GWASs were mostly correlated (Figures 5A–C). However, significant and sub-significant variants in the UMAP space formed distinct clusters (Figure 5D). *HMGCR, APOE*, and *APOC1* variants clustered together. They were equally significant for both the general population sample and for each of the sexes. The larger cluster located in the center of the space that included variants in the *PPARG, ANKKD1B, CERT1, POLK*, and other genes was divided into two clusters. The larger cluster contained variants in the *PPARG, ANKDD1B, CERT1, HMGCR*, *POLK, APOA5, APOB*, and other genes, which were more significant for men (Figure 5F), while the smaller cluster included variants in the *CERT1, POLK, NECTIN2, TOMM40, ANKDD2B*, and other genes, which were more significant for the general population sample and for women (Figures 5E–G). Another “men” cluster is located in the lower left corner and contains variants in the *CELSR2, UBR4*, and *NKAIN2* genes (Figure 5F). In contrast, the “women” cluster, consisting of variants in previously discussed *SMARCA4* and *LDLR*, is located in the lower right corner of the UMAP space (Figure 5G).

Thus, the sex-adjusted GWAS in the general population sample provided information on variants significantly associated with total cholesterol levels in at least one of the sexes. The sex-specific GWASs, however, demonstrated that the genetic architecture of cholesterol metabolism exhibits both similarities and differences between men and women. These findings underscore the importance of considering sex-specific factors in individual genetic profiling.

### 3.6 Personalized polygenic profiling

#### 3.6.1 Polygenic score models for lipid metabolism

The data obtained on the genetic architecture of lipid metabolism in the general population sample were used to create polygenic score models. Three predictive polygenic score models were developed for total cholesterol, LDL-C, and HDL-C, respectively. Furthermore, drawing on the findings from the corresponding GWAS, we constructed two polygenic score models predicting total cholesterol levels specifically in men and women. All models were created in two variants with different machine learning methods: linear regression and random forest regression. Table 2 provides the metrics used to evaluate the predictive power of the models along with other relevant parameters.

**Table 2:** Characteristics of predictive models for lipid metabolism.

Feature	Number of SNP included in model	Number of SNP with non-null weight	Covariates	Number of trees	Training set		Test set		External validation set	
					Coefficient of determination, *r*^2^	Mean absolute error (MAE)	Coefficient of determination, *r*^2^	Mean absolute error (MAE)	Coefficient of determination, *r*^2^	Mean absolute error (MAE)
Linear regression models										
Total cholesterol	156	16	Sex, age, BMI, first 10 principal components	–	0.141	0.643	0.117	0.649	0.081	0.662
Total cholesterol (men only)	82	47	Age, BMI, first 10 principal components	–	0.242	0.602	0.224	0.595	0.201	0.613
Total cholesterol (women only)	70	20	Age, BMI, first 10 principal components	–	0.132	0.648	0.090	0.628	0.064	0.633
LDL-C	77	39	Sex, age, BMI, first 10 principal components	–	0.179	0.261	0.197	0.260	0.162	0.314
HDL-C	161	18	Sex, age, BMI, first 10 principal components	–	0.123	0.594	0.137	0.578	0.097	0.606
Random forest regression models										
Total cholesterol	444	444	Sex, age, BMI, first 10 principal components	2,200	0.453	0.503	0.166	0.659	0.126	0.684
Total cholesterol (men only)	91	91	Age, BMI, first 10 principal components	1,600	0.678	0.382	0.183	0.623	0.169	0.654
Total cholesterol (women only)	191	191	Age, BMI, first 10 principal components	1,300	0.589	0.420	0.149	0.643	0.112	0.699
LDL-C	469	469	Sex, age, BMI, first 10 principal components	500	0.399	0.219	0.219	0.250	0.191	0.290
HDL-C	733	733	Sex, age, BMI, first 10 principal components	2,200	0.515	0.441	0.160	0.584	0.128	0.612

Coefficients for predictors in Linear regression models are presented in Supplementary Tables S5-S9, feature importances for Random forest regression models are presented in Supplementary Tables S10-S14.

#### 3.6.2 Polygenic score models validation and assessment

Model validation was conducted using a separate cohort that was not included in the GWAS (external validation set). The characteristics of this cohort are presented in Table 1. The validation results demonstrate that models built using random forest methods exhibit superior predictive performance even in this cohort (Table 2). These models will be used for further testing of associations with cardiovascular diseases (CVDs) and the construction of individual polygenic profiles. The only exception is the model predicting total cholesterol levels in men – for this model, the best metrics were achieved using linear regression.

However, random forest-based models do not allow for the separation of the effects of individual predictors. Therefore, for the comparison of our polygenic score models with the scales presented in the PGS Catalog database, linear models were used—specifically, the weighted sums of genetic components derived from these models (see the “Methods” section).

The results of the implementation of the PGS catalog scales (partial correlations with related lipid type level) are presented in Supplementary Tables S15–S17. Table 3 presents the comparison between the best of those scales and models created during this study.

**Table 3:** Polygenic score models comparison.

Feature	Partial correlation for the weighted sums of genetic components derived from linear regression models	Partial correlation for the weighted sums of genetic components derived from the best PGS catalog models
Total cholesterol	0.314283	0.297566 (PGS003833)
Total cholesterol (men only)	0.299360	0.260281 (PGS004675)
Total cholesterol (women only)	0.343234	0.348549 (PGS004843)
LDL-C	0.351112	0.266756 (PGS004791)
HDL-C	0.261776	0.087206 (PGS000060)

The obtained results demonstrate that nearly all models (with the exception of the total cholesterol model for women) outperform previously published scales when applied to the Russian cohort. The model predicting total cholesterol in women slightly underperforms compared to PGS004843, which showed the highest partial correlation value in this subsample. By demonstrating the high predictive power of linear regression models, we automatically validate the even greater predictive capability of random forest-based models, which previously exhibited superior performance.

#### 3.6.3 Association between polygenic scores and CVDs

The association between polygenic scores and CVDs was examined in a sample of 3,954 patients specifically recruited for this purpose (Table 1). Notably, 70.1% of these patients were prescribed lipid-regulating medications, primarily statins, which may have significantly affected their genetically determined lipid levels. Consequently, the developed models could not be validated on this sample due to the challenges associated with obtaining reliable accuracy metrics, such as the coefficient of determination and MAE.

**Figure 6:**
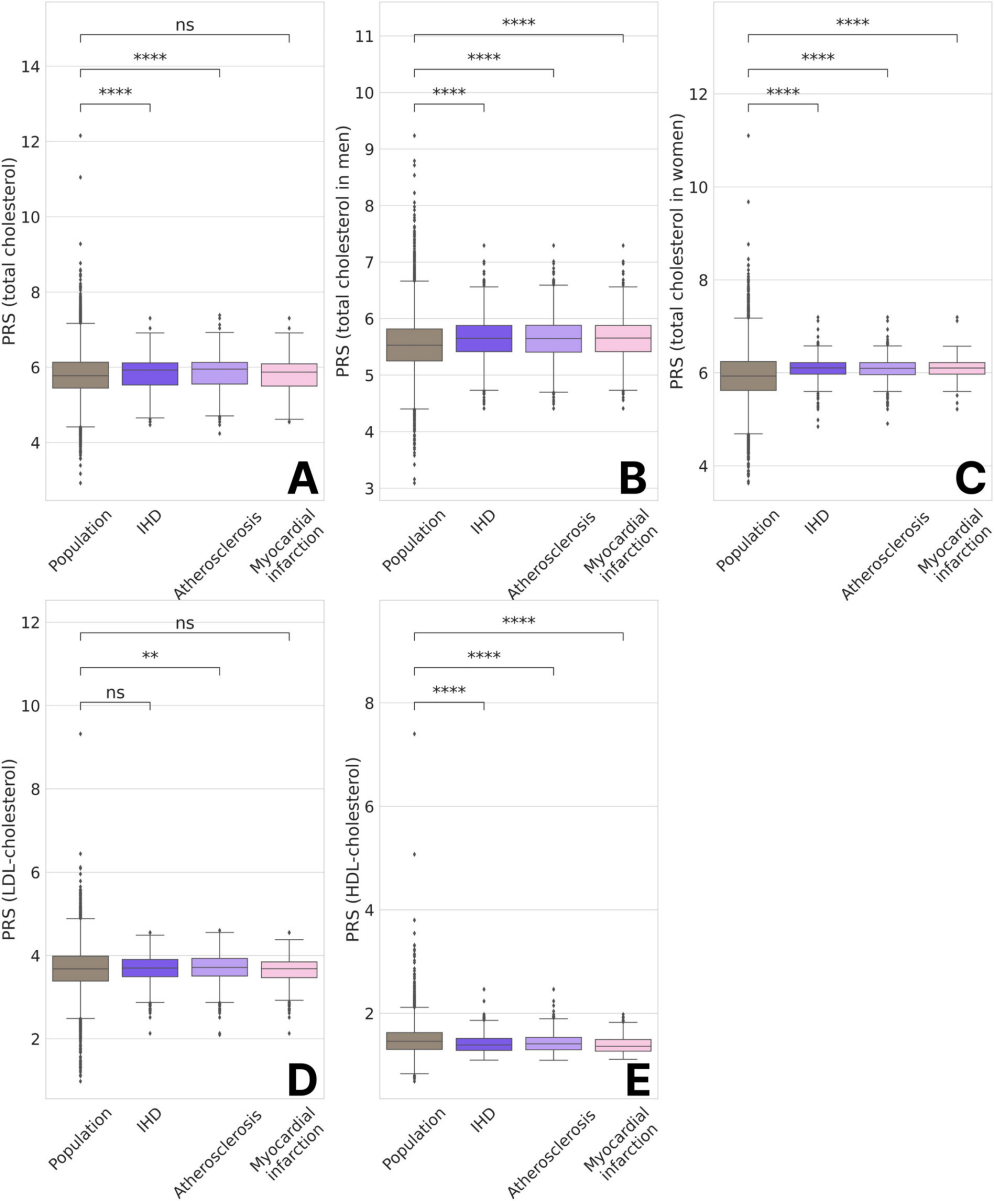
Comparison of polygenic scores calculated by five different models for the general population sample and patients with ischemic heart disease, atherosclerosis, and myocardial infarction. **(A)** Predictive model for total cholesterol levels in the general population sample. **(B)** Predictive model for total cholesterol levels in men. **(C)** Predictive model for total cholesterol levels in women. **(D)** Predictive model for LDL-C levels. **(E)** Predictive model for HDL-C levels. The asterisk denotes the significance of the differences between the groups (Mann–Whitney test): ns: 0.05 < *p* ≤ 1; **: 0.001 < *p* ≤ 0.01; ****: *p* ≤ 0.0001.

Four polygenic scores were calculated for each participant: for total cholesterol, total sex-specific cholesterol, LDL-C levels, and HDL-C levels, and the associations between the obtained polygenic scores and ischemic heart disease, atherosclerosis, and myocardial infarction were examined (Figure 6). As shown in Figure 6, the results indicated significant associations between the sex-specific polygenic scores for total cholesterol and HDL-C levels with all aforementioned cardiovascular diseases. No significant difference was found in polygenic scores for total cholesterol and LDL-C levels between the general population sample and patients with myocardial infarction; moreover, no association was found between LDL-C levels and atherosclerosis.

Table 4 outlines the relationship between the CVD risk and a one-point increase in polygenic scores expressed as the odds ratio. Notably, the sex-specific models for total cholesterol levels exhibited greater sensitivity in assessing the risk of CVDs compared with the predictive model for total cholesterol levels in the general sample: a one-score increase was associated with a higher risk of all four CVDs.

**Table 4:** Odds ratio of a one-point change in polygenic scores to the risk of CVDs; 95% CI.

Polygenic score model	Odds ratio for ischemic heart disease	Odds ratio for atherosclerosis	Odds ratio for myocardial infarction
Total cholesterol (the general population sample)	1.174 [1.081; 1.276]	1.276 [1.184; 1.376]	1.052 [0.943; 1.173]
Total cholesterol (men)	1.464 [1.304; 1.644]	1.480 [1.327; 1.651]	1.427 [1.245; 1.635]
Total cholesterol (women)	1.763 [1.526; 2.038]	1.830 [1.615; 2.073]	1.668 [1.346; 2.067]
LDL-C	1.024 [0.931; 1.125]	1.103 [1.013; 1.207]	0.921 [0.814; 1.042]
HDL-C	0.263 [0.212; 0.326]	0.332 [0.275; 0.401]	0.185 [0.138; 0.248]

#### 3.6.4 Application of polygenic score models for personalized profiling and CVD risk assessment

**Figure 7:**
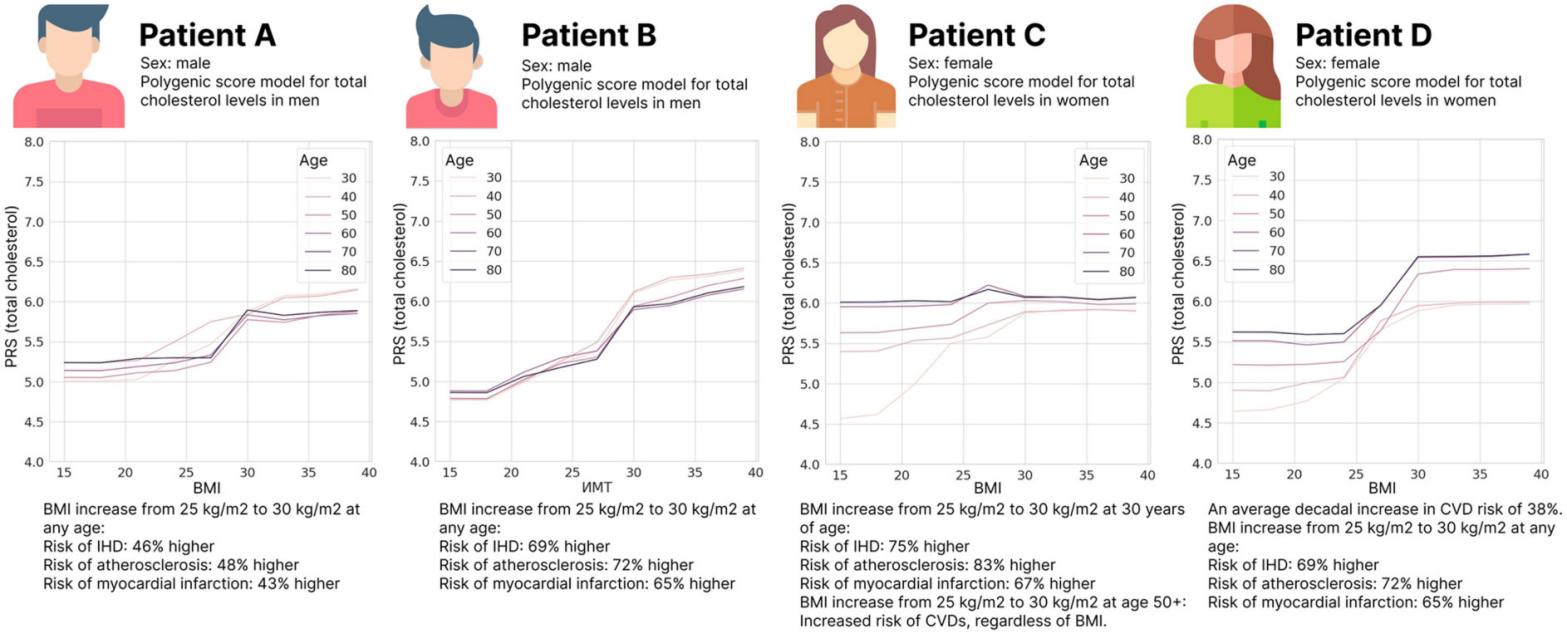
Personalized polygenic profiles based on predicted total cholesterol levels and the estimated risk of CVDs.

Sex-specific models for total cholesterol levels in men and women were used for personalized polygenic profiling of two men and two women. Predicted changes in cholesterol levels differed between the two men with different genotypes but identical variable features—age and BMI (Figures 7A,B). As shown in Figure 7B, when the BMI of both individuals increased comparably, Patient B experienced more dramatic changes in predicted cholesterol levels compared with Patient A, particularly within the younger age range of 30–40 years. Specifically, an increase in BMI from 25 kg/m^2^ to 30 kg/m^2^ resulted in a 1-point increase in the polygenic score for Patient A and a 1.5-point increase for Patient B. The associated increase in the risk of CVDs was evaluated using the odds ratios presented in Table 4.

A similar trend was observed in women (Figures 7C,D), who had the same variable features, such as age and BMI, yet showed different patterns of changes in predicted cholesterol levels. In Patient C, an increase in BMI led to increased cholesterol levels only at a younger age (Figure 7C), whereas her lipid levels remained consistently elevated in later years, irrespective of BMI. In contrast, Patient D's cholesterol levels were affected by her BMI throughout her life. However, unlike Patients A and B, who had relatively stable cholesterol levels at a constant BMI, Patient D experienced a consistent decadal increase in her predicted cholesterol levels.

## 4 Discussion

### 4.1 Genetic architecture of lipid metabolism

Many of the variants linked to lipid levels in this study are located in genes whose products are involved in the metabolism and transport of cholesterol (30) or in intergenic regions near these genes. For instance, apolipoproteins, such as *APOB, APOE, APOC1, APOC3,* and *APOC4*, which were associated with total cholesterol and/or LDL-C levels, bind to lipoproteins, facilitate their transport, and ensure lipoprotein-receptor interaction (31). The CERT1 gene is also responsible for lipid transport, but between the cell organelles (32). *HMGCR* encodes HMG-CoA reductase, which is the regulatory enzyme in cholesterol synthesis and one of the therapeutic targets for dyslipidemia (33).

Variants in the genes located on chromosome 19, specifically *TOMM40, NECTIN2, APOE,* and *APOC1*, have been associated with metabolic, cardiovascular, and neurological diseases (34). Our research group has previously established an association between this genetic locus and longevity in the Russian population, further supporting its involvement in the development of critical aging-associated diseases (35).

Sandhu et al. have also detected associations between LDL-C and variants in the *APOB, APOC1, BCAM, BCL3,* and *LDLR* genes in their genome-wide association study (36). The authors found the *CELSR2* gene, which encodes a cadherin protein, to be significant. In our study, however, this gene did not show genome-wide significance; it was sub-significant and was included in the meta-analysis. The association between LDL-C and the detected *CELSR2* variants was more common in men than in women. The *CELSR2 gene* is also part of the gene network (Figure 2) and directly interacts with the *SMARCA4* and *BCL3 genes.*

Interestingly, *SMARCA4* and *BCL3* are not directly involved in lipid metabolism. *SMARCA4* is a transcription activator. As shown in the gene network (Figure 2), it does not directly interact with the genes involved in lipid metabolism but interacts with them indirectly through *MCM3, PPARG*, and other genes. *BCL3* is a proto-oncogene (37), as are two other genes, *CBLC* (38) and *RELB,* which is a NF-kB subunit (39)*. POLK* encodes DNA polymerase, and *CLASRP* is a splicing regulator. Thus, the genes associated with lipid levels are involved in the fundamental cellular mechanisms, particularly the cell cycle. Is cholesterol accumulation another fundamental mechanism? Singh et al. showed that the inhibition of cholesterol in the cell membrane resulted in cell cycle arrest in the G1 phase (40). Singh et al. found a link between cholesterol and the proliferation of polyploid (41) and cancer cells (42). Not only cholesterol but also all other isoprenoids may be involved in cell cycle regulation (43).

Three other genes associated with both total cholesterol and LDL-C levels—*ANKDD1B, BCAM,* and *NECTIN2*—are involved in cell adhesion. This association, along with the association between the above-mentioned genes and cancer cell proliferation, suggests a potential relationship between cancer and lipid accumulation. This hypothesis has been explored in several studies, particularly in those focusing on the identification of therapeutic targets and the effect of cholesterol accumulation, as well as the genetic variants associated with it, on treatment outcomes in cancer (44). Consequently, effective cholesterol management may serve as a protective strategy against cancer, and the proposed personalized polygenic profiling could be utilized for individual risk assessment of both cardiovascular diseases and cancer.

Completely different genes were associated with HDL-C: *LPL, ALDH1A2, LIPC,* and *CETP*. The link between HDL-C levels and *LPL* (lipoprotein lipase), *LIPC* (lipase C), and *CETP* (cholesterol ester transferase) has already been shown earlier (45, 46); the link between HDL-C levels and *ALDH1A2* is a novel finding. *ALDH1A2* encodes a protein from the aldehyde dehydrogenase family and is involved in the retinoid synthesis from retinal (47), which is important for the removal of “old” molecules in the light-sensitive vision cycle and protection of the retina against photodamage (48). This gene has not been directly associated with HDL-C levels.

Intergenic and intronic variants, which constitute the majority of the findings in this study, are particularly noteworthy. The identification of these variants was facilitated by the application of whole genome sequencing, as opposed to the more commonly employed whole exome sequencing in previous studies. Based on the localization of these variants, it is reasonable to suggest that cholesterol levels may be influenced not only by mechanisms that disrupt the functionality of protein molecules but also by mechanisms that regulate transcription in the genes encoding these proteins. Furthermore, the significant number of regulatory genes identified in this study reinforces the notion that the genetic influence on lipid levels is regulatory in nature. This hypothesis requires additional transcriptomic data for validation and for the facilitation of future research.

Another noteworthy finding is the differences in lipid metabolism between men and women revealed by the comparison of the GWAS results for the general population sample and sex-specific GWASs for men and women. Using sex as a covariate in a GWAS is believed to help factor in the differences in phenotype formation between men and women. In our study, however, we observed that men and women differed not only in the risk levels for dyslipidemia, which are often affected by hormonal differences, but also in the genetic predictors of lipid levels. The genetic architecture of complex phenotypes is known to differ between men and women (49). These differences have also been observed in twin studies (50) and in studies on model organisms (51). This finding should be taken into consideration in both research and the development of diagnostic and therapeutic strategies.

### 4.2 Personalized polygenic profiling

Given the multifactorial nature of the examined phenotypes and the differences in genetic predictors observed between men and women, we chose to implement random forest algorithms for constructing polygenic score models. This approach, in contrast to the conventional linear or logistic regression methods, effectively captures the nonlinear interactions among predictors and informs whether a predictor should be taken into consideration. Elgart et al. have shown the advantages of using nonlinear machine learning models in constructing polygenic models over more traditionally used regression models (52). Although the authors used gradient boosting, a different nonlinear model type, they suggest that all nonlinear models generally offer more benefits for building polygenic models.

The constructed predictive models assess the cumulative contribution of genetics to the formation of the examined phenotypes. The contribution of the detected genetic variants was significantly lower than that of the covariates in all models. However, using personalized polygenic profiling, we demonstrated changes in predicted lipid levels resulting from an individual's genetic profile. Indeed, increased BMI is associated with increased lipid levels and higher risk of CVDs; however, the impact of these covariates varies between individuals. Personalized polygenetic profiling and mapping, similar to that presented in Figure 7, will enable personalized lifestyle recommendations for people at different levels of genetic risk of hypercholesterolemia and to identify people who are most susceptible to the disease at an early age and to take preventive measures.

The models’ coefficient of determination, which was used as the assessment metric, was not very high. However, the PGS catalog database contains numerous predictive models for cholesterol and individual types of lipids, with coefficients of determination not exceeding 0.3. Given the variability of lipid levels influenced by diet, lifestyle, and other non-genetic factors, developing more precise models based solely on genetic data may not be feasible. Nevertheless, this limitation does not undermine the potential utility of the models developed. Importantly, polygenic scores differed between the participants with CVDs and the general population sample, suggesting that the genetic variants associated with lipid levels could also inform about the risk of certain other diseases. Previous studies have established a connection between predicted LDL-C levels and ischemic heart disease (53), which contrasts with our finding. In our study, the models estimating the level of total and HDL-C were better at predicting the risk of ischemic heart disease. Additionally, it is noteworthy that the models for the general population sample outperformed the sex-specific models on the test set; however, the sex-specific models were better at predicting associations with CVDs, highlighting their relevance in clinical practice. They exhibited greater sensitivity regarding CVD risk assessment, further underscoring their advantages for personalized polygenic profiling.

Moreover, we successfully demonstrated the superior predictive performance of our models compared to previously published ones. Thus, we once again confirm the relevance of developing population-specific scales tailored to national cohorts, as opposed to the direct transfer of previously developed predictive tools.

## 5 Conclusion

The genome-wide association studies of lipid metabolism in over 8,000 Russian adults revealed novel associations. These findings highlight the necessity for further genetic research focused on ethnically diverse population groups, as well as the development of new genetic scales with enhanced ethnicity sensitivity. Moreover, the sex-specific findings provide better insight into the mechanisms underlying the phenotype formation in men and women. The polygenic score models could facilitate personalized polygenic profiling with a subsequent assessment of CVD risks. Implementing these models in clinical practice will enable personalized recommendations and assist in the identification of individuals at risk of serious health conditions, allowing for timely preventive measures.

## Data Availability

The data that support the findings of this study are not openly available due to reasons of sensitivity and are available from the corresponding author upon reasonable request. Data are located in controlled access data storage at Federal State Budgetary Institution «Centre for Strategic Planning and Management of Biomedical Health Risks» of the Federal medical and biological agency.
