# Supplementary material for: Personalized polygenic profiling based on the genetic architecture of lipid metabolism in the Russian population
Source: Front Cardiovasc Med. 2026 Jun 2;13:1707598. doi: 10.3389/fcvm.2026.1707598 (PMC13269230; doi:10.3389/fcvm.2026.1707598)
Supplement: Supplementary file 1 [file Datasheet1.docx]

***Supplementary materials***

**Supplementary table S1.** Variants significantly associated with total cholesterol levels in the general population

| **Chromosome** | **Position** | **Reference allele** | **Alterative allele** | **Allele frequency** | **Regression p-value** | **Regression coefficient** | **DBSNPID** | **Gene** |
| --- | --- | --- | --- | --- | --- | --- | --- | --- |
| 2 | 20967152 | C | T | 0.04 | 7.987E-09 | -0.1948 | rs7604788 |  |
| 2 | 21063185 | T | C | 0.81 | 6.851E-10 | 0.1056 | rs515135 |  |
| 2 | 21065354 | G | A | 0.81 | 1.644E-09 | 0.1044 | rs563290 |  |
| 2 | 21065449 | A | G | 0.81 | 1.822E-09 | 0.1043 | rs562338 |  |
| 2 | 21066560 | G | A | 0.81 | 1.966E-09 | 0.1038 | rs581411 |  |
| 3 | 82771751 | T | G | 0.02 | 2.196E-08 | -0.2926 | rs1703894187 |  |
| 5 | 75266739 | T | C | 0.47 | 4.944E-10 | 0.0842 | rs2112653 |  |
| 5 | 75272410 | G | A | 0.46 | 7.092E-10 | 0.0836 | rs1544755 |  |
| 5 | 75274031 | G | T | 0.46 | 4.471E-10 | 0.0846 | rs4704200 |  |
| 5 | 75278499 | A | C | 0.47 | 7.488E-11 | 0.0884 | rs4704202 |  |
| 5 | 75279159 | A | G | 0.46 | 2.167E-10 | 0.0862 | rs2126736 |  |
| 5 | 75279976 | A | G | 0.34 | 3.151E-08 | 0.0788 | rs1115091 |  |
| 5 | 75280460 | G | A | 0.46 | 3.331E-10 | 0.0853 | rs7722186 |  |
| 5 | 75288207 | T | C | 0.46 | 3.729E-10 | 0.0851 | rs10059435 |  |
| 5 | 75291041 | CT | C | 0.46 | 3.477E-10 | 0.0852 | rs59409412 |  |
| 5 | 75292115 | C | A | 0.35 | 3.494E-08 | 0.0784 | rs7730344 |  |
| 5 | 75292432 | G | A | 0.32 | 1.583E-08 | 0.0817 | rs12655005 |  |
| 5 | 75299369 | A | C | 0.50 | 2.044E-08 | 0.0760 | rs10942734 |  |
| 5 | 75306874 | C | A | 0.45 | 1.519E-10 | 0.0871 | rs1423527 |  |
| 5 | 75307654 | G | A | 0.47 | 3.497E-10 | 0.0852 | rs2335418 |  |
| 5 | 75308130 | C | T | 0.45 | 8.089E-10 | 0.0835 | rs10070119 |  |
| 5 | 75309395 | G | A | 0.37 | 1.162E-08 | 0.0793 | rs10056811 |  |
| 5 | 75312907 | C | T | 0.46 | 4.258E-10 | 0.0847 | rs11957260 |  |
| 5 | 75315749 | G | T | 0.46 | 2.178E-10 | 0.0861 | rs6892023 |  |
| 5 | 75317068 | G | A | 0.46 | 3.675E-10 | 0.0850 | rs7702895 |  |
| 5 | 75319196 | C | T | 0.37 | 7.516E-09 | 0.0800 | rs17671591 |  |
| 5 | 75321018 | T | C | 0.37 | 8.210E-09 | 0.0798 | rs10474433 |  |
| 5 | 75321437 | G | A | 0.44 | 5.412E-11 | 0.0893 | rs2878417 |  |
| 5 | 75322120 | G | A | 0.37 | 1.645E-08 | 0.0781 | rs79856035 |  |
| 5 | 75322361 | G | A | 0.37 | 9.037E-09 | 0.0796 | rs13358429 |  |
| 5 | 75323814 | C | T | 0.37 | 3.463E-08 | 0.0763 | rs79225634 |  |
| 5 | 75325846 | A | G | 0.44 | 9.027E-11 | 0.0882 | rs4704208 |  |
| 5 | 75326296 | TTA | T | 0.46 | 3.289E-10 | 0.0855 | rs147592913 |  |
| 5 | 75328657 | T | C | 0.43 | 3.341E-11 | 0.0909 | rs3843480 |  |
| 5 | 75329576 | C | CT | 0.41 | 2.683E-10 | 0.0868 | rs11405709 |  |
| 5 | 75329662 | C | A | 0.43 | 2.720E-11 | 0.0912 | rs7703051 |  |
| 5 | 75330257 | T | C | 0.43 | 1.928E-11 | 0.0919 | rs11749783 |  |
| 5 | 75332550 | A | T | 0.43 | 4.733E-11 | 0.0900 | rs3843481 |  |
| 5 | 75339400 | G | C | 0.42 | 2.152E-10 | 0.0862 | rs4704210 | HMGCR |
| 5 | 75340659 | C | A | 0.43 | 3.935E-11 | 0.0904 | rs10045497 | HMGCR |
| 5 | 75341527 | G | GCTTA | 0.43 | 2.694E-11 | 0.0912 | rs17238379 | HMGCR |
| 5 | 75341886 | A | T | 0.43 | 2.715E-11 | 0.0912 | rs10038095 | HMGCR |
| 5 | 75343353 | A | G | 0.45 | 1.138E-10 | 0.0878 | rs3846661 | HMGCR |
| 5 | 75343434 | T | G | 0.43 | 3.804E-11 | 0.0905 | rs3843482 | HMGCR |
| 5 | 75343719 | CTTGTA | C | 0.41 | 8.703E-10 | 0.0836 | rs3064191 | HMGCR |
| 5 | 75343751 | C | CAA | 0.43 | 2.452E-11 | 0.0914 | rs3064877 | HMGCR |
| 5 | 75344665 | C | T | 0.43 | 2.237E-11 | 0.0916 | rs2878419 | HMGCR |
| 5 | 75347023 | A | T | 0.43 | 6.404E-11 | 0.0895 | rs17244834 | HMGCR |
| 5 | 75348173 | A | ATTC | 0.45 | 6.822E-11 | 0.0889 | rs10683134 | HMGCR |
| 5 | 75348881 | T | G | 0.43 | 2.199E-11 | 0.0916 | rs6453131 | HMGCR |
| 5 | 75348960 | T | TA | 0.43 | 2.022E-11 | 0.0918 | rs11443896 | HMGCR |
| 5 | 75352778 | A | T | 0.43 | 1.969E-11 | 0.0918 | rs12654264 | HMGCR |
| 5 | 75355259 | A | G | 0.48 | 3.093E-10 | 0.0852 | rs3846662 | HMGCR |
| 5 | 75356084 | G | A | 0.45 | 9.126E-11 | 0.0883 | rs6882842 | HMGCR |
| 5 | 75359901 | C | T | 0.43 | 3.231E-11 | 0.0908 | rs3846663 | CERT1 |
| 5 | 75360714 | T | C | 0.46 | 1.853E-10 | 0.0866 | rs12916 | CERT1 |
| 5 | 75370667 | C | T | 0.43 | 1.735E-09 | 0.0822 | rs7733436 | CERT1 |
| 5 | 75393424 | T | C | 0.43 | 2.924E-09 | 0.0810 | rs6878990 | CERT1 |
| 5 | 75440775 | TA | T | 0.42 | 6.044E-09 | 0.0796 | rs11285492 | CERT1 |
| 5 | 75452680 | C | T | 0.43 | 1.948E-09 | 0.0819 | rs4704219 | CERT1 |
| 5 | 75461731 | G | A | 0.43 | 1.728E-09 | 0.0822 | rs4704220 | CERT1 |
| 5 | 75463358 | T | A | 0.43 | 2.804E-09 | 0.0811 | rs4704221 | CERT1 |
| 5 | 75472522 | C | A | 0.43 | 1.725E-09 | 0.0822 | rs6873472 | CERT1 |
| 5 | 75477595 | T | C | 0.43 | 2.286E-09 | 0.0816 | rs4704223 | CERT1 |
| 5 | 75491485 | T | C | 0.43 | 1.658E-09 | 0.0823 | rs6896136 | CERT1 |
| 5 | 75512412 | G | T | 0.43 | 7.694E-09 | 0.0788 | rs4549504 | POLK |
| 5 | 75513055 | C | T | 0.43 | 1.766E-09 | 0.0822 | rs4604177 | POLK |
| 5 | 75521119 | C | G | 0.43 | 2.506E-09 | 0.0813 | rs5744552 | POLK |
| 5 | 75524614 | G | GA | 0.53 | 9.080E-09 | -0.0770 | rs35953663 | POLK |
| 5 | 75530910 | G | A | 0.43 | 5.009E-09 | 0.0798 | rs4704227 | POLK |
| 5 | 75581978 | T | C | 0.43 | 3.602E-09 | 0.0804 | rs5744672 | POLK |
| 5 | 75584065 | G | A | 0.43 | 1.769E-09 | 0.0821 | rs5744680 | POLK |
| 5 | 75611138 | A | C | 0.43 | 1.187E-08 | 0.0776 | rs7703282 |  |
| 5 | 75614147 | C | T | 0.43 | 3.784E-09 | 0.0803 | rs11748027 | ANKDD1B |
| 5 | 75615045 | A | G | 0.43 | 4.280E-09 | 0.0801 | rs984976 | ANKDD1B |
| 11 | 116830819 | T | C | 0.68 | 2.775E-09 | -0.0849 | rs4520 | APOC3 |
| 19 | 44748549 | G | T | 0.02 | 1.093E-10 | -0.3068 | rs531660643 | BCL3 |
| 19 | 44799247 | G | A | 0.02 | 9.313E-12 | -0.3338 | rs148933445 | CBLC |
| 19 | 44799692 | GAA | G | 0.02 | 8.117E-12 | -0.3353 | rs201837204 | CBLC |
| 19 | 44816374 | G | A | 0.03 | 1.916E-11 | -0.2703 | rs118147862 | BCAM |
| 19 | 44879418 | G | A | 0.03 | 6.101E-14 | -0.2822 | rs41290120 | NECTIN2 |
| 19 | 44883210 | G | GTAA | 0.20 | 1.235E-08 | 0.0968 | rs142042446 | NECTIN2 |
| 19 | 44884202 | C | G | 0.20 | 1.278E-08 | 0.0967 | rs12972156 | NECTIN2 |
| 19 | 44884339 | G | A | 0.20 | 1.100E-08 | 0.0972 | rs12972970 | NECTIN2 |
| 19 | 44884873 | G | A | 0.20 | 4.571E-08 | 0.0920 | rs34342646 | NECTIN2 |
| 19 | 44886339 | G | A | 0.04 | 4.821E-08 | -0.1874 | rs7254892 | NECTIN2 |
| 19 | 44888997 | C | T | 0.22 | 3.608E-09 | 0.0965 | rs6857 | NECTIN2 |
| 19 | 44891079 | T | C | 0.20 | 2.172E-08 | 0.0942 | rs71352238 | TOMM40 |
| 19 | 44892362 | A | G | 0.20 | 1.689E-09 | 0.1026 | rs2075650 | TOMM40 |
| 19 | 44892652 | C | G | 0.20 | 1.925E-09 | 0.1023 | rs34404554 | TOMM40 |
| 19 | 44892887 | C | T | 0.20 | 2.569E-09 | 0.1015 | rs11556505 | TOMM40 |
| 19 | 44906745 | G | A | 0.10 | 1.408E-12 | 0.1583 | rs769449 | APOE |
| 19 | 44908684 | T | C | 0.13 | 1.360E-13 | 0.1498 | rs429358 | APOE |
| 19 | 44908822 | C | T | 0.07 | 4.048E-22 | -0.2516 | rs7412 | APOE |
| 19 | 44909976 | G | T | 0.07 | 2.496E-22 | -0.2523 | rs1065853 |  |
| 19 | 44911142 | C | A | 0.09 | 6.057E-15 | -0.1776 | rs72654473 |  |
| 19 | 44912383 | G | A | 0.10 | 1.817E-13 | -0.1660 | rs445925 |  |
| 19 | 44912456 | G | A | 0.10 | 2.864E-13 | 0.1620 | rs10414043 |  |
| 19 | 44912678 | G | T | 0.10 | 2.636E-13 | 0.1626 | rs7256200 |  |
| 19 | 44913574 | T | G | 0.10 | 2.253E-13 | -0.1651 | rs390082 |  |
| 19 | 44916968 | T | TA | 0.02 | 1.698E-12 | -0.3324 |  | APOC1 |
| 19 | 44917997 | G | A | 0.14 | 3.817E-12 | 0.1354 | rs12721046 | APOC1 |
| 19 | 44918903 | C | G | 0.17 | 1.459E-11 | 0.1220 | rs12721051 | APOC1 |
| 19 | 44919589 | G | A | 0.17 | 1.321E-11 | 0.1223 | rs56131196 |  |
| 19 | 44919689 | A | G | 0.17 | 1.731E-11 | 0.1215 | rs4420638 |  |
| 19 | 44920730 | C | CA | 0.14 | 1.546E-10 | 0.1232 |  |  |
| 19 | 44921094 | A | T | 0.17 | 1.096E-11 | 0.1227 | rs814573 |  |
| 19 | 44921095 | A | T | 0.14 | 2.710E-12 | 0.1350 | rs56369833 |  |
| 19 | 44921257 | A | C | 0.16 | 5.900E-10 | 0.1137 | rs157592 |  |
| 19 | 44923868 | T | A | 0.14 | 2.495E-12 | 0.1349 | rs111789331 |  |
| 19 | 44924977 | G | A | 0.14 | 3.094E-12 | 0.1344 | rs66626994 |  |
| 19 | 44943964 | G | A | 0.02 | 2.518E-09 | -0.2940 | rs12721109 | APOC4 |
| 19 | 11076648 | C | G | 0.08 | 2.786E-08 | -0.1388 | rs143020224 | SMARCA4 |
| 19 | 11076682 | T | G | 0.08 | 2.542E-08 | -0.1392 | rs144826254 | SMARCA4 |
| 19 | 11076746 | T | C | 0.08 | 2.549E-08 | -0.1391 | rs112736558 | SMARCA4 |
| 19 | 11076935 | A | G | 0.08 | 2.169E-08 | -0.1397 | rs111989435 | SMARCA4 |
| 19 | 11077441 | C | T | 0.08 | 4.341E-08 | -0.1374 | rs55997232 | SMARCA4 |
| 19 | 11077477 | A | C | 0.08 | 4.669E-08 | -0.1370 | rs55791371 | SMARCA4 |
| 19 | 11077488 | T | C | 0.08 | 3.880E-08 | -0.1379 | rs56125973 | SMARCA4 |
| 19 | 11077571 | G | A | 0.08 | 2.450E-08 | -0.1399 | rs56289821 | SMARCA4 |
| 19 | 11077637 | C | CTTTA | 0.08 | 4.032E-08 | -0.1378 | rs55990223 | SMARCA4 |
| 19 | 11078174 | T | C | 0.08 | 3.056E-08 | -0.1389 | rs112898275 | SMARCA4 |
| 19 | 11078298 | AC | A | 0.08 | 4.768E-08 | -0.1373 | rs374982520 | SMARCA4 |
| 19 | 11078529 | C | G | 0.08 | 3.681E-08 | -0.1382 | rs148898583 | SMARCA4 |
| 19 | 11078596 | T | C | 0.08 | 4.875E-08 | -0.1369 | rs113722226 | SMARCA4 |
| 19 | 11078690 | C | CAG | 0.08 | 4.514E-08 | -0.1373 | rs201408139 | SMARCA4 |
| 19 | 11079088 | T | C | 0.08 | 4.900E-08 | -0.1368 | rs73015011 | SMARCA4 |
| 19 | 11079261 | T | A | 0.08 | 3.446E-08 | -0.1384 | rs114821903 | SMARCA4 |
| 19 | 11079304 | C | A | 0.08 | 4.116E-08 | -0.1376 | rs138175288 | SMARCA4 |
| 19 | 11079398 | G | A | 0.08 | 3.874E-08 | -0.1379 | rs112107114 | SMARCA4 |
| 19 | 11079434 | A | G | 0.08 | 4.116E-08 | -0.1376 | rs115594766 |  |
| 19 | 11079616 | T | C | 0.08 | 3.675E-08 | -0.1382 | rs112032422 |  |
| 19 | 11079805 | G | T | 0.08 | 4.456E-08 | -0.1374 | rs77265569 |  |
| 19 | 11079858 | G | A | 0.08 | 4.433E-08 | -0.1372 | rs142158911 |  |
| 19 | 11080521 | G | A | 0.08 | 4.433E-08 | -0.1372 | rs114846969 |  |
| 19 | 11081053 | C | T | 0.08 | 4.125E-08 | -0.1376 | rs138294113 |  |
| 19 | 11082415 | T | G | 0.08 | 4.810E-08 | -0.1369 | rs112552009 |  |
| 19 | 11085680 | AC | A | 0.08 | 4.927E-08 | -0.1368 | rs139306531 |  |
| 19 | 11086585 | G | A | 0.08 | 4.693E-08 | -0.1370 | rs12151108 |  |
| 19 | 11086922 | G | T | 0.08 | 3.317E-08 | -0.1386 | rs73015024 |  |
| 19 | 11087074 | G | GA | 0.08 | 3.424E-08 | -0.1387 |  |  |

**Supplementary table S2.** Variants significantly associated with LDL-C levels in the general population sample

| **Chromosome** | **Position** | **Reference allele** | **Alterative allele** | **Allele frequency** | **Regression p-value** | **Regression coefficient** | **DBSNPID** | **Gene** |
| --- | --- | --- | --- | --- | --- | --- | --- | --- |
| 2 | 20951027 | T | C | 0.60 | 2.287E-11 | -0.0826 | rs4075673 |  |
| 2 | 20993943 | C | A | 0.33 | 1.730E-10 | 0.0823 | rs62122481 |  |
| 2 | 21016795 | T | C | 0.35 | 7.233E-10 | 0.0785 | rs12720816 | APOB |
| 2 | 21041028 | G | A | 0.30 | 3.376E-08 | 0.0733 | rs1367117 | APOB |
| 2 | 21050024 | G | A | 0.14 | 4.565E-09 | -0.1008 | rs35913552 |  |
| 2 | 21051295 | A | T | 0.14 | 4.817E-09 | -0.1006 | rs13011615 |  |
| 2 | 21063185 | T | C | 0.81 | 9.965E-12 | 0.1047 | rs515135 |  |
| 2 | 21065354 | G | A | 0.81 | 8.202E-12 | 0.1063 | rs563290 |  |
| 2 | 21065449 | A | G | 0.81 | 2.238E-11 | 0.1042 | rs562338 |  |
| 2 | 21066560 | G | A | 0.81 | 1.223E-11 | 0.1053 | rs581411 |  |
| 2 | 21080598 | T | C | 0.12 | 4.740E-08 | -0.1016 | rs7567217 |  |
| 2 | 21086250 | C | T | 0.12 | 4.230E-08 | -0.1022 | rs28562532 |  |
| 2 | 21086984 | G | A | 0.12 | 4.399E-08 | -0.1021 | rs6756284 |  |
| 2 | 21092201 | AT | A | 0.12 | 4.625E-08 | -0.1021 | rs35789251 |  |
| 2 | 21194704 | C | T | 0.18 | 2.007E-08 | -0.0880 | rs4596008 |  |
| 2 | 21195211 | G | A | 0.18 | 3.274E-08 | -0.0867 | rs67599264 |  |
| 2 | 21195847 | C | A | 0.18 | 2.171E-08 | -0.0877 | rs36047821 |  |
| 2 | 21195884 | A | G | 0.18 | 2.037E-08 | -0.0879 | rs34125138 |  |
| 2 | 21196448 | C | G | 0.18 | 2.161E-08 | -0.0877 | rs11897480 |  |
| 2 | 21196778 | G | A | 0.23 | 1.553E-08 | -0.0822 | rs10221742 |  |
| 2 | 21196937 | A | T | 0.18 | 1.909E-08 | -0.0881 | rs10221876 |  |
| 2 | 21197486 | C | A | 0.18 | 2.056E-08 | -0.0879 | rs1878512 |  |
| 2 | 21197825 | A | G | 0.18 | 2.016E-08 | -0.0879 | rs17041988 |  |
| 2 | 21198910 | C | A | 0.18 | 2.011E-08 | -0.0879 | rs12712923 |  |
| 2 | 21199531 | G | A | 0.18 | 1.683E-08 | -0.0884 | rs7578527 |  |
| 2 | 21200056 | C | T | 0.18 | 3.367E-08 | -0.0868 | rs17042000 |  |
| 2 | 21201117 | T | C | 0.18 | 2.860E-08 | -0.0871 | rs10169543 |  |
| 2 | 21202451 | A | G | 0.18 | 3.126E-08 | -0.0868 | rs34002646 |  |
| 2 | 21203871 | C | A | 0.18 | 2.646E-08 | -0.0873 | rs12712940 |  |
| 2 | 21248731 | T | C | 0.79 | 9.092E-09 | 0.0851 | rs1848922 |  |
| 5 | 75065315 | T | C | 0.18 | 4.394E-08 | 0.0857 | rs4703645 |  |
| 5 | 75099216 | T | C | 0.20 | 4.894E-08 | 0.0831 | rs9765094 | ANKRD31 |
| 5 | 75117888 | T | C | 0.20 | 3.199E-08 | 0.0842 | rs2035191 | ANKRD31 |
| 5 | 75220642 | CA | C | 0.36 | 1.147E-08 | 0.0723 | rs113439471 | ANKRD31 |
| 5 | 75232565 | GTT | G | 0.36 | 1.351E-08 | 0.0711 |  | ANKRD31 |
| 5 | 75266739 | T | C | 0.47 | 1.188E-09 | 0.0740 | rs2112653 |  |
| 5 | 75272410 | G | A | 0.46 | 1.047E-09 | 0.0743 | rs1544755 |  |
| 5 | 75274031 | G | T | 0.46 | 8.823E-10 | 0.0747 | rs4704200 |  |
| 5 | 75278499 | A | C | 0.47 | 1.237E-10 | 0.0785 | rs4704202 |  |
| 5 | 75279159 | A | G | 0.46 | 4.193E-10 | 0.0761 | rs2126736 |  |
| 5 | 75280460 | G | A | 0.46 | 6.061E-10 | 0.0755 | rs7722186 |  |
| 5 | 75288207 | T | C | 0.46 | 5.008E-10 | 0.0758 | rs10059435 |  |
| 5 | 75291041 | CT | C | 0.46 | 4.934E-10 | 0.0759 | rs59409412 |  |
| 5 | 75292432 | G | A | 0.32 | 3.273E-08 | 0.0718 | rs12655005 |  |
| 5 | 75306874 | C | A | 0.45 | 8.209E-10 | 0.0750 | rs1423527 |  |
| 5 | 75307654 | G | A | 0.47 | 1.985E-09 | 0.0731 | rs2335418 |  |
| 5 | 75308130 | C | T | 0.45 | 3.248E-09 | 0.0722 | rs10070119 |  |
| 5 | 75309395 | G | A | 0.37 | 5.211E-09 | 0.0729 | rs10056811 |  |
| 5 | 75312907 | C | T | 0.46 | 1.861E-09 | 0.0732 | rs11957260 |  |
| 5 | 75315749 | G | T | 0.46 | 8.850E-10 | 0.0747 | rs6892023 |  |
| 5 | 75317068 | G | A | 0.46 | 1.268E-09 | 0.0740 | rs7702895 |  |
| 5 | 75319196 | C | T | 0.37 | 5.350E-09 | 0.0725 | rs17671591 |  |
| 5 | 75321018 | T | C | 0.37 | 6.233E-09 | 0.0722 | rs10474433 |  |
| 5 | 75321437 | G | A | 0.44 | 2.261E-10 | 0.0775 | rs2878417 |  |
| 5 | 75322120 | G | A | 0.37 | 9.966E-09 | 0.0712 | rs79856035 |  |
| 5 | 75322361 | G | A | 0.37 | 6.833E-09 | 0.0720 | rs13358429 |  |
| 5 | 75323814 | C | T | 0.37 | 1.974E-08 | 0.0698 | rs79225634 |  |
| 5 | 75325749 | A | AT | 0.41 | 4.182E-09 | 0.0727 | rs34966499 |  |
| 5 | 75325846 | A | G | 0.44 | 2.634E-10 | 0.0772 | rs4704208 |  |
| 5 | 75326296 | TTA | T | 0.46 | 9.267E-10 | 0.0748 | rs147592913 |  |
| 5 | 75328657 | T | C | 0.43 | 1.137E-10 | 0.0793 | rs3843480 |  |
| 5 | 75329576 | C | CT | 0.41 | 4.264E-10 | 0.0770 | rs11405709 |  |
| 5 | 75329662 | C | A | 0.43 | 4.679E-11 | 0.0809 | rs7703051 |  |
| 5 | 75330257 | T | C | 0.43 | 5.480E-11 | 0.0806 | rs11749783 |  |
| 5 | 75332550 | A | T | 0.43 | 1.399E-10 | 0.0788 | rs3843481 |  |
| 5 | 75339400 | G | C | 0.42 | 6.543E-10 | 0.0753 | rs4704210 | HMGCR |
| 5 | 75340659 | C | A | 0.43 | 5.426E-11 | 0.0806 | rs10045497 | HMGCR |
| 5 | 75341527 | G | GCTTA | 0.43 | 7.581E-11 | 0.0800 | rs17238379 | HMGCR |
| 5 | 75341886 | A | T | 0.43 | 7.431E-11 | 0.0800 | rs10038095 | HMGCR |
| 5 | 75343353 | A | G | 0.45 | 2.695E-10 | 0.0772 | rs3846661 | HMGCR |
| 5 | 75343434 | T | G | 0.43 | 1.090E-10 | 0.0793 | rs3843482 | HMGCR |
| 5 | 75343719 | CTTGTA | C | 0.41 | 2.557E-09 | 0.0730 | rs3064191 | HMGCR |
| 5 | 75343751 | C | CAA | 0.43 | 6.771E-11 | 0.0803 | rs3064877 | HMGCR |
| 5 | 75344665 | C | T | 0.43 | 5.970E-11 | 0.0804 | rs2878419 | HMGCR |
| 5 | 75347023 | A | T | 0.43 | 1.767E-10 | 0.0785 | rs17244834 | HMGCR |
| 5 | 75348173 | A | ATTC | 0.45 | 1.239E-10 | 0.0787 | rs10683134 | HMGCR |
| 5 | 75348881 | T | G | 0.43 | 2.971E-11 | 0.0817 | rs6453131 | HMGCR |
| 5 | 75348960 | T | TA | 0.43 | 5.410E-11 | 0.0806 | rs11443896 | HMGCR |
| 5 | 75352778 | A | T | 0.43 | 5.515E-11 | 0.0806 | rs12654264 | HMGCR |
| 5 | 75355259 | A | G | 0.48 | 1.330E-09 | 0.0737 | rs3846662 | HMGCR |
| 5 | 75356084 | G | A | 0.45 | 2.081E-10 | 0.0778 | rs6882842 | HMGCR |
| 5 | 75359901 | C | T | 0.43 | 7.659E-11 | 0.0800 | rs3846663 | CERT1 |
| 5 | 75360714 | T | C | 0.46 | 8.264E-10 | 0.0749 | rs12916 | CERT1 |
| 5 | 75370667 | C | T | 0.43 | 9.020E-09 | 0.0705 | rs7733436 | CERT1 |
| 5 | 75393424 | T | C | 0.43 | 1.318E-08 | 0.0697 | rs6878990 | CERT1 |
| 5 | 75440775 | TA | T | 0.42 | 1.283E-08 | 0.0699 | rs11285492 | CERT1 |
| 5 | 75452680 | C | T | 0.43 | 8.291E-09 | 0.0706 | rs4704219 | CERT1 |
| 5 | 75461731 | G | A | 0.43 | 7.977E-09 | 0.0707 | rs4704220 | CERT1 |
| 5 | 75463358 | T | A | 0.43 | 1.023E-08 | 0.0702 | rs4704221 | CERT1 |
| 5 | 75472522 | C | A | 0.43 | 8.679E-09 | 0.0706 | rs6873472 | CERT1 |
| 5 | 75477595 | T | C | 0.43 | 7.863E-09 | 0.0708 | rs4704223 | CERT1 |
| 5 | 75491485 | T | C | 0.43 | 8.229E-09 | 0.0707 | rs6896136 | CERT1 |
| 5 | 75512412 | G | T | 0.43 | 2.717E-08 | 0.0681 | rs4549504 | POLK |
| 5 | 75513055 | C | T | 0.43 | 1.101E-08 | 0.0701 | rs4604177 | POLK |
| 5 | 75521119 | C | G | 0.43 | 8.574E-09 | 0.0705 | rs5744552 | POLK |
| 5 | 75530910 | G | A | 0.43 | 1.717E-08 | 0.0691 | rs4704227 | POLK |
| 5 | 75581978 | T | C | 0.43 | 1.325E-08 | 0.0696 | rs5744672 | POLK |
| 5 | 75584065 | G | A | 0.43 | 6.354E-09 | 0.0712 | rs5744680 | POLK |
| 5 | 75614147 | C | T | 0.43 | 1.790E-08 | 0.0689 | rs11748027 | ANKDD1B |
| 5 | 75615045 | A | G | 0.43 | 1.867E-08 | 0.0688 | rs984976 | ANKDD1B |
| 19 | 44537330 | C | T | 0.02 | 4.925E-08 | -0.2256 | rs144439590 | CEACAM22P |
| 19 | 44728895 | G | A | 0.03 | 1.022E-13 | -0.2576 | rs62117160 |  |
| 19 | 44738916 | G | A | 0.05 | 2.006E-09 | -0.1629 | rs1531517 |  |
| 19 | 44739710 | C | T | 0.05 | 1.551E-09 | -0.1640 | rs62117204 |  |
| 19 | 44740135 | A | AAT | 0.03 | 1.170E-12 | -0.2474 | rs201359001 |  |
| 19 | 44744370 | A | G | 0.05 | 2.134E-09 | -0.1686 | rs4803750 |  |
| 19 | 44748549 | G | T | 0.02 | 7.767E-13 | -0.3060 | rs531660643 | BCL3 |
| 19 | 44750234 | C | T | 0.11 | 1.041E-08 | -0.1080 | rs10401176 | BCL3 |
| 19 | 44750354 | ATTGGC | A | 0.11 | 1.748E-08 | -0.1071 | rs66586168 | BCL3 |
| 19 | 44752009 | T | C | 0.05 | 5.044E-09 | -0.1662 | rs62117205 | BCL3 |
| 19 | 44752422 | G | C | 0.05 | 2.709E-09 | -0.1677 | rs62117206 | BCL3 |
| 19 | 44799247 | G | A | 0.02 | 1.624E-14 | -0.3377 | rs148933445 | CBLC |
| 19 | 44799692 | GAA | G | 0.02 | 3.565E-15 | -0.3466 | rs201837204 | CBLC |
| 19 | 44816374 | G | A | 0.03 | 7.568E-19 | -0.3203 | rs118147862 | BCAM |
| 19 | 44827846 | A | G | 0.64 | 1.764E-09 | 0.0758 | rs7343130 |  |
| 19 | 44862466 | TA | T | 0.10 | 2.639E-08 | -0.1100 | rs60608124 | NECTIN2 |
| 19 | 44865946 | G | A | 0.03 | 6.849E-09 | -0.2055 | rs112422902 | NECTIN2 |
| 19 | 44879418 | G | A | 0.03 | 1.277E-25 | -0.3532 | rs41290120 | NECTIN2 |
| 19 | 44881148 | T | C | 0.12 | 4.617E-08 | -0.1029 | rs73052307 | NECTIN2 |
| 19 | 44883210 | G | GTAA | 0.20 | 2.366E-09 | 0.0913 | rs142042446 | NECTIN2 |
| 19 | 44883777 | A | C | 0.05 | 1.284E-11 | -0.1812 | rs283808 | NECTIN2 |
| 19 | 44883800 | A | G | 0.05 | 1.232E-11 | -0.1814 | rs283809 | NECTIN2 |
| 19 | 44884202 | C | G | 0.20 | 2.444E-09 | 0.0912 | rs12972156 | NECTIN2 |
| 19 | 44884339 | G | A | 0.20 | 2.364E-09 | 0.0913 | rs12972970 | NECTIN2 |
| 19 | 44884873 | G | A | 0.20 | 2.286E-09 | 0.0904 | rs34342646 | NECTIN2 |
| 19 | 44886339 | G | A | 0.04 | 8.598E-17 | -0.2565 | rs7254892 | NECTIN2 |
| 19 | 44888997 | C | T | 0.22 | 2.740E-10 | 0.0928 | rs6857 | NECTIN2 |
| 19 | 44889415 | AT | A | 0.04 | 1.918E-15 | -0.2333 | rs111300402 |  |
| 19 | 44891079 | T | C | 0.20 | 5.735E-10 | 0.0938 | rs71352238 | TOMM40 |
| 19 | 44892362 | A | G | 0.20 | 4.440E-10 | 0.0955 | rs2075650 | TOMM40 |
| 19 | 44892652 | C | G | 0.20 | 5.142E-10 | 0.0952 | rs34404554 | TOMM40 |
| 19 | 44892887 | C | T | 0.20 | 4.691E-10 | 0.0954 | rs11556505 | TOMM40 |
| 19 | 44893972 | G | A | 0.04 | 4.137E-17 | -0.2715 | rs1160983 | TOMM40 |
| 19 | 44897490 | T | A | 0.04 | 1.335E-17 | -0.2748 | rs61679753 | TOMM40 |
| 19 | 44899005 | T | G | 0.04 | 1.580E-17 | -0.2740 | rs111784051 | TOMM40 |
| 19 | 44905371 | T | C | 0.07 | 3.063E-08 | -0.1272 | rs769446 |  |
| 19 | 44906745 | G | A | 0.10 | 1.891E-14 | 0.1536 | rs769449 | APOE |
| 19 | 44908684 | T | C | 0.13 | 1.178E-16 | 0.1506 | rs429358 | APOE |
| 19 | 44908822 | C | T | 0.07 | 9.803E-46 | -0.3309 | rs7412 | APOE |
| 19 | 44909976 | G | T | 0.07 | 4.922E-46 | -0.3312 | rs1065853 |  |
| 19 | 44911142 | C | A | 0.09 | 1.471E-29 | -0.2306 | rs72654473 |  |
| 19 | 44912383 | G | A | 0.10 | 3.574E-27 | -0.2184 | rs445925 |  |
| 19 | 44912456 | G | A | 0.10 | 2.923E-15 | 0.1573 | rs10414043 |  |
| 19 | 44912678 | G | T | 0.10 | 2.940E-15 | 0.1576 | rs7256200 |  |
| 19 | 44913574 | T | G | 0.10 | 8.234E-27 | -0.2166 | rs390082 |  |
| 19 | 44916968 | T | TA | 0.02 | 6.326E-15 | -0.3302 |  | APOC1 |
| 19 | 44917997 | G | A | 0.14 | 1.484E-14 | 0.1347 | rs12721046 | APOC1 |
| 19 | 44918903 | C | G | 0.17 | 8.417E-15 | 0.1259 | rs12721051 | APOC1 |
| 19 | 44919589 | G | A | 0.17 | 1.597E-14 | 0.1246 | rs56131196 |  |
| 19 | 44919689 | A | G | 0.17 | 2.056E-14 | 0.1240 | rs4420638 |  |
| 19 | 44920730 | C | CA | 0.14 | 3.363E-13 | 0.1258 |  |  |
| 19 | 44921094 | A | T | 0.17 | 6.576E-14 | 0.1216 | rs814573 |  |
| 19 | 44921095 | A | T | 0.14 | 1.999E-14 | 0.1327 | rs56369833 |  |
| 19 | 44921257 | A | C | 0.16 | 5.130E-14 | 0.1241 | rs157592 |  |
| 19 | 44923868 | T | A | 0.14 | 4.760E-15 | 0.1355 | rs111789331 |  |
| 19 | 44924977 | G | A | 0.14 | 5.449E-15 | 0.1353 | rs66626994 |  |
| 19 | 44930070 | T | TA | 0.08 | 4.377E-08 | -0.1208 | rs34193823 | APOC1P1 |
| 19 | 44943964 | G | A | 0.02 | 8.602E-12 | -0.3029 | rs12721109 | APOC4 |
| 19 | 45028360 | GT | G | 0.02 | 3.073E-08 | -0.2246 | rs201897835 | RELB |
| 19 | 45038524 | G | A | 0.02 | 3.746E-08 | -0.2269 | rs34827707 |  |
| 19 | 45041227 | C | CA | 0.03 | 2.340E-09 | -0.2266 | rs397945551 | CLASRP |
| 19 | 45150625 | TAGTC | T | 0.02 | 1.465E-08 | -0.2478 | rs201531065 | NKPD1 |
| 19 | 11076648 | C | G | 0.08 | 5.479E-10 | -0.1395 | rs143020224 | SMARCA4 |
| 19 | 11076682 | T | G | 0.08 | 4.043E-10 | -0.1406 | rs144826254 | SMARCA4 |
| 19 | 11076746 | T | C | 0.08 | 4.847E-10 | -0.1399 | rs112736558 | SMARCA4 |
| 19 | 11076935 | A | G | 0.08 | 4.027E-10 | -0.1405 | rs111989435 | SMARCA4 |
| 19 | 11077441 | C | T | 0.08 | 1.059E-09 | -0.1377 | rs55997232 | SMARCA4 |
| 19 | 11077477 | A | C | 0.08 | 8.964E-10 | -0.1383 | rs55791371 | SMARCA4 |
| 19 | 11077488 | T | C | 0.08 | 1.121E-09 | -0.1375 | rs56125973 | SMARCA4 |
| 19 | 11077571 | G | A | 0.08 | 1.859E-09 | -0.1357 | rs56289821 | SMARCA4 |
| 19 | 11077637 | C | CTTTA | 0.08 | 7.006E-10 | -0.1393 | rs55990223 | SMARCA4 |
| 19 | 11077868 | CT | C | 0.08 | 4.237E-09 | -0.1350 | rs57223420 | SMARCA4 |
| 19 | 11078174 | T | C | 0.08 | 6.995E-10 | -0.1392 | rs112898275 | SMARCA4 |
| 19 | 11078223 | C | T | 0.08 | 1.052E-09 | -0.1393 | rs112374545 | SMARCA4 |
| 19 | 11078298 | AC | A | 0.08 | 8.180E-10 | -0.1390 | rs374982520 | SMARCA4 |
| 19 | 11078371 | TA | T | 0.08 | 1.315E-09 | -0.1376 | rs201579954 | SMARCA4 |
| 19 | 11078529 | C | G | 0.08 | 7.028E-10 | -0.1393 | rs148898583 | SMARCA4 |
| 19 | 11078596 | T | C | 0.08 | 1.056E-09 | -0.1378 | rs113722226 | SMARCA4 |
| 19 | 11078622 | CG | C | 0.08 | 1.107E-09 | -0.1376 | rs200495339 | SMARCA4 |
| 19 | 11078690 | C | CAG | 0.08 | 6.920E-10 | -0.1393 | rs201408139 | SMARCA4 |
| 19 | 11079088 | T | C | 0.08 | 9.590E-10 | -0.1381 | rs73015011 | SMARCA4 |
| 19 | 11079261 | T | A | 0.08 | 7.206E-10 | -0.1391 | rs114821903 | SMARCA4 |
| 19 | 11079304 | C | A | 0.08 | 8.697E-10 | -0.1384 | rs138175288 | SMARCA4 |
| 19 | 11079398 | G | A | 0.08 | 6.999E-10 | -0.1393 | rs112107114 | SMARCA4 |
| 19 | 11079434 | A | G | 0.08 | 8.697E-10 | -0.1384 | rs115594766 |  |
| 19 | 11079616 | T | C | 0.08 | 7.708E-10 | -0.1389 | rs112032422 |  |
| 19 | 11079781 | GAAAA | G | 0.07 | 1.450E-08 | -0.1327 | rs368404285 |  |
| 19 | 11079805 | G | T | 0.08 | 8.696E-10 | -0.1386 | rs77265569 |  |
| 19 | 11079858 | G | A | 0.08 | 9.373E-10 | -0.1381 | rs142158911 |  |
| 19 | 11079868 | C | T | 0.08 | 1.028E-09 | -0.1378 | rs118068660 |  |
| 19 | 11079873 | G | A | 0.08 | 1.305E-09 | -0.1369 | rs145960625 |  |
| 19 | 11079880 | T | C | 0.08 | 1.215E-09 | -0.1372 | rs139853365 |  |
| 19 | 11079976 | G | A | 0.08 | 1.258E-09 | -0.1371 | rs142130958 |  |
| 19 | 11080197 | C | T | 0.08 | 1.153E-09 | -0.1374 | rs73015013 |  |
| 19 | 11080521 | G | A | 0.08 | 9.373E-10 | -0.1381 | rs114846969 |  |
| 19 | 11080525 | A | AG | 0.08 | 1.013E-08 | -0.1272 | rs151113958 |  |
| 19 | 11080624 | G | A | 0.08 | 1.498E-08 | -0.1258 | rs73015016 |  |
| 19 | 11081001 | T | A | 0.08 | 1.579E-08 | -0.1255 | rs10402112 |  |
| 19 | 11081053 | C | T | 0.08 | 8.160E-10 | -0.1386 | rs138294113 |  |
| 19 | 11081517 | A | T | 0.08 | 1.386E-08 | -0.1260 | rs61194703 |  |
| 19 | 11081874 | G | A | 0.08 | 1.360E-08 | -0.1261 | rs73015020 |  |
| 19 | 11082155 | A | G | 0.08 | 1.351E-08 | -0.1261 | rs77140532 |  |
| 19 | 11082200 | C | CT | 0.08 | 6.775E-09 | -0.1285 |  |  |
| 19 | 11082239 | A | G | 0.08 | 9.716E-09 | -0.1275 | rs73015021 |  |
| 19 | 11082415 | T | G | 0.08 | 8.038E-10 | -0.1387 | rs112552009 |  |
| 19 | 11083273 | A | G | 0.08 | 1.692E-08 | -0.1252 | rs10412048 |  |
| 19 | 11085680 | AC | A | 0.08 | 9.232E-10 | -0.1382 | rs139306531 |  |
| 19 | 11086210 | T | C | 0.08 | 1.310E-08 | -0.1265 | rs8106503 |  |
| 19 | 11086585 | G | A | 0.08 | 8.043E-10 | -0.1387 | rs12151108 |  |
| 19 | 11086922 | G | T | 0.08 | 6.551E-10 | -0.1395 | rs73015024 |  |
| 19 | 11087074 | G | GA | 0.08 | 4.422E-10 | -0.1411 |  |  |
| 19 | 11087511 | C | T | 0.08 | 1.820E-08 | -0.1253 | rs17248720 |  |
| 19 | 11087826 | T | C | 0.08 | 2.072E-09 | -0.1349 | rs17248727 |  |
| 19 | 11090448 | T | C | 0.08 | 1.142E-09 | -0.1373 | rs57217136 | LDLR |
| 19 | 11091312 | GT | G | 0.08 | 2.430E-09 | -0.1358 | rs60173709 | LDLR |
| 19 | 11091518 | GC | G | 0.08 | 2.089E-09 | -0.1364 | rs141787760 | LDLR |
| 19 | 11091630 | G | T | 0.08 | 1.912E-09 | -0.1366 | rs6511720 | LDLR |

**Supplementary table S3.** Variants significantly associated with HDL-C levels in the general population sample

| **Chromosome** | **Position** | **Reference allele** | **Alterative allele** | **Allele frequency** | **Regression p-value** | **Regression coefficient** | **DBSNPID** | **Gene** |
| --- | --- | --- | --- | --- | --- | --- | --- | --- |
| 8 | 19961928 | A | G | 0.29 | 1.738E-08 | 0.0356 | rs326 | LPL |
| 8 | 19966137 | A | T | 0.28 | 1.450E-08 | 0.0359 | rs3208305 | LPL |
| 8 | 19966981 | T | C | 0.28 | 6.833E-09 | 0.0367 | rs13702 | LPL |
| 8 | 19967156 | C | T | 0.28 | 5.301E-09 | 0.0370 | rs15285 | LPL |
| 8 | 20002028 | G | A | 0.29 | 4.659E-09 | 0.0370 | rs2119690 |  |
| 8 | 20009934 | C | G | 0.29 | 3.870E-08 | 0.0345 | rs11986942 |  |
| 15 | 58381250 | GA | G | 0.34 | 6.164E-09 | 0.0351 |  | ALDH1A2 |
| 15 | 58382109 | G | A | 0.34 | 2.019E-09 | 0.0362 | rs2043082 | ALDH1A2 |
| 15 | 58383833 | A | T | 0.31 | 2.776E-08 | 0.0344 | rs80123226 | ALDH1A2 |
| 15 | 58384622 | G | A | 0.31 | 4.608E-08 | 0.0338 | rs11858759 | ALDH1A2 |
| 15 | 58386313 | C | T | 0.29 | 2.301E-09 | 0.0375 | rs10468017 | ALDH1A2 |
| 15 | 58386521 | T | C | 0.67 | 8.474E-11 | -0.0396 | rs261290 | ALDH1A2 |
| 15 | 58387469 | G | A | 0.33 | 7.785E-11 | 0.0394 | rs7350789 | ALDH1A2 |
| 15 | 58387608 | CAGA | C | 0.33 | 9.472E-11 | 0.0392 | rs58719229 | ALDH1A2 |
| 15 | 58387979 | T | C | 0.34 | 4.572E-10 | 0.0376 | rs261291 | ALDH1A2 |
| 15 | 58387985 | C | T | 0.33 | 7.758E-11 | 0.0394 | rs7177289 | ALDH1A2 |
| 15 | 58388439 | T | C | 0.36 | 5.572E-09 | 0.0346 | rs2414577 | ALDH1A2 |
| 15 | 58388440 | T | C | 0.36 | 5.428E-09 | 0.0346 | rs2414578 | ALDH1A2 |
| 15 | 58388444 | G | T | 0.34 | 1.312E-09 | 0.0367 | rs35853021 | ALDH1A2 |
| 15 | 58388755 | T | C | 0.64 | 2.568E-08 | -0.0331 | rs2043085 | ALDH1A2 |
| 15 | 58390297 | G | C | 0.17 | 3.981E-08 | 0.0415 | rs2043084 | ALDH1A2 |
| 15 | 58391167 | A | G | 0.64 | 4.022E-08 | -0.0327 | rs1532085 | ALDH1A2 |
| 15 | 58391343 | G | C | 0.16 | 6.204E-09 | 0.0454 | rs12905732 | ALDH1A2 |
| 15 | 58391858 | A | G | 0.17 | 1.255E-08 | 0.0428 | rs11853674 | ALDH1A2 |
| 15 | 58396988 | T | C | 0.16 | 2.042E-08 | 0.0439 | rs11855284 | ALDH1A2 |
| 15 | 58399949 | G | T | 0.16 | 1.346E-09 | 0.0472 | rs35128881 | ALDH1A2 |
| 15 | 58400516 | C | T | 0.17 | 1.176E-08 | 0.0431 | rs12906722 | ALDH1A2 |
| 15 | 58405635 | C | G | 0.15 | 4.814E-08 | 0.0430 | rs12440032 | ALDH1A2 |
| 15 | 58406811 | C | T | 0.15 | 2.533E-08 | 0.0439 | rs11856159 | ALDH1A2 |
| 15 | 58409732 | A | G | 0.15 | 4.905E-08 | 0.0430 | rs150364420 | ALDH1A2 |
| 15 | 58410665 | T | C | 0.18 | 2.670E-08 | 0.0410 | rs17821310 | ALDH1A2 |
| 15 | 58563549 | C | T | 0.01 | 6.913E-12 | 0.1716 | rs113298164 | LIPC |
| 15 | 58567196 | G | A | 0.01 | 1.920E-10 | 0.1669 | rs111285504 | LIPC |
| 18 | 71032991 | C | T | 0.03 | 6.095E-09 | 0.1034 | rs117174082 |  |
| 18 | 71056827 | C | T | 0.02 | 1.455E-08 | 0.1042 | rs146635818 |  |
| 18 | 71062516 | G | A | 0.02 | 1.135E-08 | 0.1052 | rs117047314 |  |
| 18 | 71099062 | T | C | 0.02 | 2.683E-08 | 0.1027 | rs117749892 |  |
| 18 | 49656294 | A | G | 0.77 | 9.986E-09 | 0.0391 | rs1540037 |  |
| 18 | 49681721 | A | G | 0.66 | 2.633E-08 | 0.0336 | rs8084599 |  |
| 18 | 49682332 | A | G | 0.66 | 1.863E-08 | 0.0341 | rs9946453 |  |
| 18 | 49685882 | A | G | 0.67 | 2.729E-08 | 0.0337 | rs8088929 |  |
| 16 | 56951227 | A | G | 0.64 | 3.104E-13 | 0.0433 | rs9989419 |  |
| 16 | 56951244 | A | G | 0.68 | 1.136E-11 | 0.0413 | rs193695 |  |
| 16 | 56951602 | G | A | 0.33 | 2.370E-32 | 0.0715 | rs72786786 |  |
| 16 | 56951643 | A | G | 0.80 | 1.808E-11 | 0.0476 | rs12448528 |  |
| 16 | 56952850 | G | T | 0.43 | 5.638E-19 | -0.0512 | rs7203286 |  |
| 16 | 56953103 | C | T | 0.34 | 9.927E-45 | 0.0837 | rs12446515 |  |
| 16 | 56953457 | T | C | 0.34 | 3.993E-44 | 0.0829 | rs56156922 |  |
| 16 | 56953853 | C | T | 0.33 | 1.512E-43 | 0.0835 | rs56228609 |  |
| 16 | 56954132 | C | T | 0.35 | 9.934E-41 | 0.0791 | rs173539 |  |
| 16 | 56955678 | C | T | 0.34 | 9.147E-46 | 0.0848 | rs247616 |  |
| 16 | 56955918 | G | A | 0.43 | 5.810E-20 | -0.0524 | rs12923459 |  |
| 16 | 56956804 | C | A | 0.34 | 5.717E-46 | 0.0850 | rs247617 |  |
| 16 | 56957451 | C | T | 0.34 | 8.390E-46 | 0.0848 | rs183130 |  |
| 16 | 56957712 | G | A | 0.17 | 4.228E-14 | -0.0577 | rs28888131 |  |
| 16 | 56957829 | C | T | 0.16 | 6.553E-14 | -0.0573 | rs12934632 |  |
| 16 | 56959113 | G | T | 0.27 | 3.356E-10 | -0.0401 | rs12920974 |  |
| 16 | 56959249 | G | A | 0.33 | 7.519E-44 | 0.0838 | rs12149545 |  |
| 16 | 56959299 | T | C | 0.17 | 7.616E-14 | -0.0571 | rs12708967 |  |
| 16 | 56959412 | C | A | 0.34 | 1.910E-45 | 0.0844 | rs3764261 |  |
| 16 | 56959997 | A | C | 0.55 | 3.654E-22 | 0.0547 | rs711751 |  |
| 16 | 56960300 | T | C | 0.27 | 1.915E-10 | -0.0406 | rs12720918 |  |
| 16 | 56960332 | T | TA | 0.34 | 1.570E-45 | 0.0844 | rs36229491 |  |
| 16 | 56960616 | C | T | 0.34 | 4.188E-45 | 0.0840 | rs17231506 |  |
| 16 | 56960982 | G | A | 0.50 | 2.721E-14 | 0.0432 | rs4783961 |  |
| 16 | 56961324 | C | A | 0.51 | 4.393E-34 | 0.0687 | rs1800775 |  |
| 16 | 56962246 | T | C | 0.51 | 1.272E-34 | 0.0692 | rs3816117 | CETP |
| 16 | 56962299 | G | A | 0.45 | 2.089E-36 | 0.0718 | rs711752 | CETP |
| 16 | 56962376 | G | A | 0.45 | 1.658E-36 | 0.0719 | rs708272 | CETP |
| 16 | 56962733 | GCC | G | 0.45 | 2.241E-36 | 0.0718 |  | CETP |
| 16 | 56962737 | C | A | 0.45 | 4.260E-36 | 0.0715 | rs34620476 | CETP |
| 16 | 56963321 | G | A | 0.25 | 2.004E-25 | -0.0684 | rs1864163 | CETP |
| 16 | 56963437 | C | CA | 0.25 | 6.399E-26 | -0.0691 | rs5817082 | CETP |
| 16 | 56964660 | G | C | 0.26 | 1.415E-16 | -0.0536 | rs9929488 | CETP |
| 16 | 56965006 | A | G | 0.45 | 9.718E-34 | 0.0691 | rs12720926 | CETP |
| 16 | 56965346 | A | C | 0.20 | 6.635E-27 | -0.0762 | rs7203984 | CETP |
| 16 | 56965416 | C | T | 0.44 | 2.180E-35 | 0.0709 | rs11508026 | CETP |
| 16 | 56965866 | CG | C | 0.18 | 1.986E-30 | -0.0850 | rs17231569 | CETP |
| 16 | 56966768 | CT | C | 0.19 | 4.988E-19 | -0.0651 | rs35404403 | CETP |
| 16 | 56966784 | T | A | 0.19 | 1.432E-29 | -0.0815 | rs8045855 | CETP |
| 16 | 56966973 | G | A | 0.18 | 9.484E-31 | -0.0855 | rs12720922 | CETP |
| 16 | 56967026 | G | A | 0.10 | 2.360E-17 | -0.0797 | rs118146573 | CETP |
| 16 | 56967304 | C | T | 0.44 | 1.540E-35 | 0.0710 | rs4784741 | CETP |
| 16 | 56967342 | T | TCACA | 0.18 | 8.559E-31 | -0.0855 | rs12720908 | CETP |
| 16 | 56967362 | AC | A | 0.32 | 3.398E-41 | 0.0821 | rs200751500 | CETP |
| 16 | 56967526 | G | A | 0.44 | 1.438E-35 | 0.0710 | rs12444012 | CETP |
| 16 | 56968751 | C | G | 0.71 | 8.419E-21 | 0.0586 | rs9926440 | CETP |
| 16 | 56968820 | T | G | 0.80 | 1.674E-32 | 0.0844 | rs9939224 | CETP |
| 16 | 56969234 | T | C | 0.09 | 2.834E-12 | -0.0684 | rs11076174 | CETP |
| 16 | 56970977 | G | A | 0.45 | 3.325E-36 | 0.0714 | rs7205804 | CETP |
| 16 | 56971389 | C | T | 0.45 | 2.303E-37 | 0.0726 | rs1532625 | CETP |
| 16 | 56971567 | C | A | 0.45 | 2.649E-37 | 0.0726 | rs1532624 | CETP |
| 16 | 56972466 | A | G | 0.17 | 8.154E-29 | -0.0830 | rs11076175 | CETP |
| 16 | 56972678 | C | T | 0.18 | 1.518E-28 | -0.0818 | rs7499892 | CETP |
| 16 | 56972917 | T | A | 0.80 | 3.362E-26 | 0.0753 | rs289713 | CETP |
| 16 | 56973534 | T | G | 0.17 | 2.997E-19 | -0.0685 | rs11076176 | CETP |
| 16 | 56975476 | G | A | 0.34 | 1.235E-08 | -0.0340 | rs289717 | CETP |
| 16 | 56976320 | C | T | 0.10 | 1.013E-08 | 0.0547 | rs56208677 | CETP |
| 16 | 56976574 | C | T | 0.03 | 2.181E-08 | -0.0902 | rs117427818 | CETP |
| 16 | 56977273 | G | A | 0.35 | 9.146E-09 | -0.0341 | rs4784744 | CETP |
| 16 | 56977540 | G | A | 0.34 | 1.533E-08 | -0.0338 | rs291044 | CETP |
| 16 | 56978787 | A | G | 0.33 | 1.788E-08 | -0.0339 | rs291043 | CETP |
| 16 | 56980963 | A | G | 0.33 | 2.119E-09 | -0.0359 | rs4784745 | CETP |
| 22 | 47938294 | G | GA | 0.01 | 4.985E-08 | 0.1489 |  | EPIC1 |

**Supplementary table S4.** Variants significantly associated with total cholesterol levels in women

| **Chromosome** | **Position** | **Reference allele** | **Alterative allele** | **Allele frequency** | **Regression p-value** | **Regression coefficient** | **DBSNPID** | **Gene** |
| --- | --- | --- | --- | --- | --- | --- | --- | --- |
| 19 | 11076648 | C | G | 0.08 | 2.412E-08 | -0.1817 | rs143020224 | SMARCA4 |
| 19 | 11076682 | T | G | 0.08 | 2.383E-08 | -0.1817 | rs144826254 | SMARCA4 |
| 19 | 11076746 | T | C | 0.08 | 2.095E-08 | -0.1824 | rs112736558 | SMARCA4 |
| 19 | 11076935 | A | G | 0.08 | 1.589E-08 | -0.1838 | rs111989435 | SMARCA4 |
| 19 | 11077441 | C | T | 0.08 | 4.560E-08 | -0.1788 | rs55997232 | SMARCA4 |
| 19 | 11077477 | A | C | 0.08 | 4.049E-08 | -0.1794 | rs55791371 | SMARCA4 |
| 19 | 11077488 | T | C | 0.08 | 3.443E-08 | -0.1805 | rs56125973 | SMARCA4 |
| 19 | 11077637 | C | CTTTA | 0.08 | 3.758E-08 | -0.1800 | rs55990223 | SMARCA4 |
| 19 | 11078174 | T | C | 0.08 | 2.241E-08 | -0.1828 | rs112898275 | SMARCA4 |
| 19 | 11078298 | AC | A | 0.08 | 3.698E-08 | -0.1802 | rs374982520 | SMARCA4 |
| 19 | 11078529 | C | G | 0.08 | 3.557E-08 | -0.1803 | rs148898583 | SMARCA4 |
| 19 | 11078596 | T | C | 0.08 | 4.695E-08 | -0.1787 | rs113722226 | SMARCA4 |
| 19 | 11078690 | C | CAG | 0.08 | 4.040E-08 | -0.1796 | rs201408139 | SMARCA4 |
| 19 | 11079088 | T | C | 0.08 | 4.615E-08 | -0.1788 | rs73015011 | SMARCA4 |
| 19 | 11079261 | T | A | 0.08 | 3.204E-08 | -0.1809 | rs114821903 | SMARCA4 |
| 19 | 11079304 | C | A | 0.08 | 4.049E-08 | -0.1794 | rs138175288 | SMARCA4 |
| 19 | 11079398 | G | A | 0.08 | 3.880E-08 | -0.1798 | rs112107114 | SMARCA4 |
| 19 | 11079616 | T | C | 0.08 | 3.204E-08 | -0.1809 | rs112032422 |  |
| 19 | 11079805 | G | T | 0.07 | 3.360E-08 | -0.1809 | rs77265569 |  |
| 19 | 11079858 | G | A | 0.08 | 4.049E-08 | -0.1794 | rs142158911 |  |
| 19 | 11079868 | C | T | 0.08 | 4.711E-08 | -0.1786 | rs118068660 |  |
| 19 | 11079873 | G | A | 0.08 | 4.460E-08 | -0.1789 | rs145960625 |  |
| 19 | 11080197 | C | T | 0.08 | 4.788E-08 | -0.1785 | rs73015013 |  |
| 19 | 11080521 | G | A | 0.08 | 4.049E-08 | -0.1794 | rs114846969 |  |
| 19 | 11081053 | C | T | 0.08 | 4.671E-08 | -0.1787 | rs138294113 |  |
| 19 | 11082415 | T | G | 0.08 | 4.443E-08 | -0.1789 | rs112552009 |  |
| 19 | 11085680 | AC | A | 0.08 | 4.102E-08 | -0.1794 | rs139306531 |  |
| 19 | 11086585 | G | A | 0.08 | 4.264E-08 | -0.1792 | rs12151108 |  |
| 19 | 11086922 | G | T | 0.08 | 2.521E-08 | -0.1821 | rs73015024 |  |
| 19 | 11087074 | G | GA | 0.07 | 4.128E-08 | -0.1798 |  |  |
| 19 | 44816374 | G | A | 0.03 | 2.109E-10 | -0.3321 | rs118147862 | BCAM |
| 19 | 44879418 | G | A | 0.03 | 3.556E-12 | -0.3388 | rs41290120 | NECTIN2 |
| 19 | 44906745 | G | A | 0.11 | 3.882E-08 | 0.1524 | rs769449 | APOE |
| 19 | 44908684 | T | C | 0.14 | 2.359E-09 | 0.1496 | rs429358 | APOE |
| 19 | 44908822 | C | T | 0.07 | 4.625E-15 | -0.2610 | rs7412 | APOE |
| 19 | 44909521 | CT | C | 0.06 | 3.373E-08 | 0.1972 | rs747519137 |  |
| 19 | 44909976 | G | T | 0.07 | 5.917E-15 | -0.2596 | rs1065853 |  |
| 19 | 44911142 | C | A | 0.09 | 6.053E-09 | -0.1686 | rs72654473 |  |
| 19 | 44912456 | G | A | 0.11 | 1.386E-08 | 0.1568 | rs10414043 |  |
| 19 | 44912678 | G | T | 0.11 | 1.228E-08 | 0.1575 | rs7256200 |  |
| 19 | 44916968 | T | TA | 0.02 | 2.846E-08 | -0.3420 |  | APOC1 |
| 19 | 44917997 | G | A | 0.14 | 2.122E-09 | 0.1459 | rs12721046 | APOC1 |
| 19 | 44918903 | C | G | 0.18 | 4.429E-10 | 0.1405 | rs12721051 | APOC1 |
| 19 | 44919589 | G | A | 0.18 | 5.763E-10 | 0.1396 | rs56131196 |  |
| 19 | 44919689 | A | G | 0.18 | 5.938E-10 | 0.1394 | rs4420638 |  |
| 19 | 44920730 | C | CA | 0.15 | 1.514E-08 | 0.1359 |  |  |
| 19 | 44921094 | A | T | 0.18 | 3.865E-10 | 0.1410 | rs814573 |  |
| 19 | 44921095 | A | T | 0.15 | 1.298E-09 | 0.1463 | rs56369833 |  |
| 19 | 44921257 | A | C | 0.16 | 4.706E-08 | 0.1252 | rs157592 |  |
| 19 | 44923868 | T | A | 0.15 | 2.151E-09 | 0.1440 | rs111789331 |  |
| 19 | 44924977 | G | A | 0.15 | 1.819E-09 | 0.1447 | rs66626994 |  |

**Supplementary table S5.** Feature coefficients in the Linear regresssion polygenic score model for total cholesterol levels in the general population sample

| **Predictor** | **Coefficient** |
| --- | --- |
| CHR19_44909976_G_T | -0.07058 |
| CHR19_44908822_C_T | 0 |
| CHR19_44911142_C_A | -0.00976 |
| CHR19_44879418_G_A | 0 |
| CHR19_44908684_T_C | 0.042941 |
| CHR19_44912383_G_A | 0 |
| CHR19_44913574_T_G | 0 |
| CHR19_44912678_G_T | 0 |
| CHR19_44912456_G_A | 0 |
| CHR19_44906745_G_A | 0 |
| CHR19_44916968_T_TA | 0 |
| CHR19_44923868_T_A | 0 |
| CHR19_44921095_A_T | 0 |
| CHR19_44924977_G_A | 0.009358 |
| CHR19_44917997_G_A | 0 |
| CHR19_44799692_GAA_G | 0 |
| CHR19_44799247_G_A | 0 |
| CHR19_44921094_A_T | 0 |
| CHR19_44919589_G_A | 0 |
| CHR19_44918903_C_G | 0 |
| CHR19_44919689_A_G | 0 |
| CHR19_44816374_G_A | 0 |
| CHR5_75330257_T_C | 0 |
| CHR5_75352778_A_T | 0 |
| CHR5_75348960_T_TA | 0 |
| CHR5_75348881_T_G | 0 |
| CHR5_75344665_C_T | 0 |
| CHR5_75343751_C_CAA | 0 |
| CHR5_75341527_G_GCTTA | 0 |
| CHR5_75341886_A_T | 0.00328 |
| CHR5_75329662_C_A | 0 |
| CHR5_75359901_C_T | 0 |
| CHR5_75328657_T_C | 0 |
| CHR5_75343434_T_G | 0 |
| CHR5_75340659_C_A | 0 |
| CHR5_75332550_A_T | 0 |
| CHR5_75321437_G_A | 0 |
| CHR5_75347023_A_T | 0 |
| CHR5_75348173_A_ATTC | 0 |
| CHR5_75278499_A_C | 0.005773 |
| CHR5_75325846_A_G | 0 |
| CHR5_75356084_G_A | 0 |
| CHR19_44748549_G_T | 0 |
| CHR5_75343353_A_G | 0 |
| CHR5_75306874_C_A | 0 |
| CHR19_44920730_C_CA | 0 |
| CHR5_75360714_T_C | 0.026684 |
| CHR5_75339400_G_C | 0 |
| CHR5_75279159_A_G | 0 |
| CHR5_75315749_G_T | 0 |
| CHR5_75329576_C_CT | 0 |
| CHR5_75355259_A_G | 0.00539 |
| CHR5_75326296_TTA_T | 0 |
| CHR5_75280460_G_A | 0 |
| CHR5_75291041_CT_C | 0 |
| CHR5_75307654_G_A | 0 |
| CHR5_75317068_G_A | 0 |
| CHR5_75288207_T_C | 0 |
| CHR5_75312907_C_T | 0 |
| CHR5_75274031_G_T | 0 |
| CHR5_75266739_T_C | 0 |
| CHR19_44921257_A_C | 0 |
| CHR2_21063185_T_C | 0.040338 |
| CHR5_75272410_G_A | 0 |
| CHR5_75308130_C_T | 0 |
| CHR5_75343719_CTTGTA_C | 0 |
| CHR2_21065354_G_A | 0 |
| CHR5_75491485_T_C | 0 |
| CHR19_44892362_A_G | 0 |
| CHR5_75472522_C_A | 0 |
| CHR5_75461731_G_A | 0 |
| CHR5_75370667_C_T | 0 |
| CHR5_75513055_C_T | 0 |
| CHR5_75584065_G_A | 0 |
| CHR2_21065449_A_G | 0.0083 |
| CHR19_44892652_C_G | 0.010381 |
| CHR5_75452680_C_T | 0 |
| CHR2_21066560_G_A | 0 |
| CHR5_75477595_T_C | 0 |
| CHR5_75521119_C_G | 0 |
| CHR19_44943964_G_A | 0 |
| CHR19_44892887_C_T | 0 |
| CHR11_116830819_T_C | -0.05055 |
| CHR5_75463358_T_A | 0 |
| CHR5_75393424_T_C | 0 |
| CHR5_75581978_T_C | 0 |
| CHR19_44888997_C_T | 0 |
| CHR5_75614147_C_T | 0 |
| CHR5_75615045_A_G | 0 |
| CHR5_75530910_G_A | 0 |
| CHR5_75440775_TA_T | 0 |
| CHR5_75319196_C_T | 0 |
| CHR5_75512412_G_T | 0 |
| CHR2_20967152_C_T | 0 |
| CHR5_75321018_T_C | 0 |
| CHR5_75322361_G_A | 0 |
| CHR5_75524614_G_GA | 0 |
| CHR19_44884339_G_A | 0 |
| CHR5_75309395_G_A | 0 |
| CHR5_75611138_A_C | 0 |
| CHR19_44883210_G_GTAA | 0 |
| CHR19_44884202_C_G | 0 |
| CHR6_61022872_G_C | 0.032696 |
| CHR5_75292432_G_A | 0 |
| CHR5_75322120_G_A | 0 |
| CHR5_75299369_A_C | 0 |
| CHR19_11076935_A_G | 0 |
| CHR19_44891079_T_C | 0 |
| CHR3_82771751_T_G | -0.06864 |
| CHR19_11077571_G_A | 0 |
| CHR19_11076682_T_G | 0 |
| CHR19_11076746_T_C | 0 |
| CHR19_11076648_C_G | 0 |
| CHR19_11078174_T_C | 0 |
| CHR5_75279976_A_G | 0 |
| CHR19_11086922_G_T | 0 |
| CHR19_11087074_G_GA | 0 |
| CHR19_11079261_T_A | 0 |
| CHR5_75323814_C_T | 0 |
| CHR5_75292115_C_A | 0.013067 |
| CHR19_11079616_T_C | 0 |
| CHR19_11078529_C_G | 0 |
| CHR19_11079398_G_A | 0 |
| CHR19_11077488_T_C | 0 |
| CHR19_11077637_C_CTTTA | 0 |
| CHR19_11079434_A_G | 0 |
| CHR19_11079304_C_A | 0 |
| CHR19_11081053_C_T | 0 |
| CHR19_11077441_C_T | 0 |
| CHR19_11080521_G_A | 0 |
| CHR19_11079858_G_A | 0 |
| CHR19_11079805_G_T | 0 |
| CHR19_11078690_C_CAG | 0 |
| CHR19_44884873_G_A | 0 |
| CHR19_11077477_A_C | 0 |
| CHR19_11086585_G_A | 0 |
| CHR19_11078298_AC_A | 0 |
| CHR19_11082415_T_G | 0 |
| CHR19_44886339_G_A | 0 |
| CHR19_11078596_T_C | 0 |
| CHR19_11079088_T_C | 0 |
| CHR19_11085680_AC_A | 0 |
| CHR19_11080197_C_T | 0 |
| CHR19_11079868_C_T | 0 |
| CHR19_11090448_T_C | 0 |
| CHR19_11079873_G_A | 0 |
| CHR19_11078622_CG_C | 0 |
| CHR19_11079976_G_A | 0 |
| CHR19_11079880_T_C | 0 |
| CHR5_75308917_G_A | 0 |
| CHR5_75638184_A_T | 0 |
| CHR5_75276089_G_A | 0.008734 |
| CHR19_11078223_C_T | 0 |
| CHR19_44728895_G_A | 0 |
| CHR19_11078371_TA_T | 0 |
| CHR19_11087826_T_C | 0 |
| Sex_from_meta_manual | -0.15194 |
| age | 0.110934 |
| bmi | 0.182363 |
| pc0 | 0 |
| pc1 | 0.038897 |
| pc2 | 0 |
| pc3 | 0 |
| pc4 | -0.00033 |
| pc5 | 0.034454 |
| pc6 | 0 |
| pc7 | 0 |
| pc8 | -0.00755 |
| pc9 | -0.01006 |

**Supplementary table S6.** Feature coefficients in the Linear regresssion the polygenic score model for total cholesterol levels in men

| **Predictor** | **Coefficient** |
| --- | --- |
| CHR19_44909976_G_T | -0.07391 |
| CHR19_44908822_C_T | 0 |
| CHR19_44911142_C_A | 0 |
| CHR11_54748602_A_G | 0.025336 |
| CHR5_16841935_T_G | 0.026447 |
| CHR12_109256961_T_C | 0.039694 |
| CHR5_6473277_AC_A | 0.052603 |
| CHR12_109255543_G_A | 0.001905 |
| CHR11_48813072_C_G | 0 |
| CHR3_3380082_A_G | 0.06038 |
| CHR11_116830819_T_C | -0.06107 |
| CHR19_44912383_G_A | 0 |
| CHR19_44913574_T_G | 0 |
| CHR1_109273124_A_G | -0.05169 |
| CHR11_54748607_T_G | 0 |
| CHR2_20967152_C_T | -0.03979 |
| CHR1_90670307_A_C | -0.02852 |
| CHR1_90670938_A_G | -0.00058 |
| CHR1_109272589_C_A | -0.00132 |
| CHR6_124075586_T_G | -0.0386 |
| CHR6_61022872_G_C | 0.000591 |
| CHR6_124074142_C_T | 0 |
| CHR6_124075032_T_G | 0 |
| CHR1_90696169_T_C | 0 |
| CHR12_40627202_TA_T | 0 |
| CHR1_90667647_C_T | 0 |
| CHR11_485086_C_CTG | 0.052119 |
| CHR1_90696212_G_A | 0 |
| CHR1_90694029_T_C | 0 |
| CHR1_90678498_T_C | 0 |
| CHR3_3382734_T_G | 0 |
| CHR1_90696496_T_C | 0 |
| CHR1_90664497_T_C | 0 |
| CHR1_109272746_T_C | -0.00021 |
| CHR14_63590232_G_T | 0.034556 |
| CHR1_90670660_T_C | -0.00437 |
| CHR3_3377075_G_T | 0 |
| CHR1_90677429_T_C | 0 |
| CHR3_3382548_A_G | 0 |
| CHR1_90662056_C_CA | -0.01569 |
| CHR1_90698570_G_T | 0 |
| CHR6_124074155_T_C | -0.01442 |
| CHR1_90701982_CT_C | 0 |
| CHR2_204522432_C_A | 0.062245 |
| CHR1_90662481_G_A | 0 |
| CHR1_90658106_T_G | 0 |
| CHR9_92552190_TAC_T | 0.035353 |
| CHR5_75315408_G_T | 0.062103 |
| CHR2_91933274_C_T | 0 |
| CHR1_19076679_C_T | -0.05291 |
| CHR1_90686913_G_T | 0 |
| CHR19_7308862_T_A | 0.065059 |
| CHR3_3380195_G_T | 0 |
| CHR2_214940439_T_G | 0.033108 |
| CHR3_3382648_C_A | -0.02294 |
| CHR12_114023417_A_C | 0.047653 |
| CHR20_11081373_A_C | 0.042565 |
| CHR6_72325416_T_C | 0.021435 |
| CHR9_81715347_CA_C | 0.05265 |
| CHR3_3380423_C_T | 0 |
| CHR1_109272511_G_A | 0 |
| CHR12_114024939_G_C | 0.001096 |
| CHR16_12736607_C_T | 0.051478 |
| CHR5_163429761_G_A | 0.029104 |
| CHR12_114023754_G_GT | 2.65E-05 |
| CHR1_19154483_G_A | 0 |
| CHR6_52264411_T_G | 0.045973 |
| CHR6_30986961_G_A | -0.01554 |
| CHR11_116770056_C_CAA | 0.022429 |
| CHR2_20951027_T_C | -0.04337 |
| CHR1_90685900_G_A | 0 |
| CHR16_12739559_C_G | 0.0016 |
| CHR2_7976831_G_A | 0.058667 |
| CHR12_114021567_A_G | 0 |
| CHR10_130623353_C_A | -0.05383 |
| CHR1_19273912_C_T | 0 |
| CHR3_3382270_C_T | 0 |
| CHR4_77969558_TA_T | 0.032036 |
| CHR5_163350156_T_A | 0.02661 |
| CHR8_127459937_A_C | 0.047205 |
| CHR2_123570834_G_T | 0.024555 |
| CHR2_21011100_T_C | 0 |
| age | -0.0181 |
| bmi | 0.239466 |
| pc0 | 0.02885 |
| pc1 | 0.059011 |
| pc2 | 0 |
| pc3 | -0.01928 |
| pc4 | 0 |
| pc5 | 0.074825 |
| pc6 | 0 |
| pc7 | 0.002953 |
| pc8 | 0 |
| pc9 | -0.01011 |

**Supplementary table S7.** Feature coefficients in the Linear regresssion polygenic score model for total cholesterol levels in women

| **Predictor** | **Coefficient** |
| --- | --- |
| CHR19_44908822_C_T | -0,03287 |
| CHR19_44909976_G_T | -0,02235 |
| CHR19_44879418_G_A | -0,03678 |
| CHR19_44816374_G_A | 0 |
| CHR19_44921094_A_T | 0,027961 |
| CHR19_44918903_C_G | 0,003731 |
| CHR19_44919589_G_A | 0,006401 |
| CHR19_44919689_A_G | 0,007416 |
| CHR19_44921095_A_T | 0 |
| CHR19_44924977_G_A | 0 |
| CHR19_44917997_G_A | 0 |
| CHR19_44923868_T_A | 0 |
| CHR19_44908684_T_C | 0 |
| CHR19_44911142_C_A | 0 |
| CHR19_44912678_G_T | 0 |
| CHR19_44912456_G_A | 0 |
| CHR19_44920730_C_CA | 0 |
| CHR19_11076935_A_G | -0,02798 |
| CHR19_11076746_T_C | -0,00504 |
| CHR19_11078174_T_C | -0,01116 |
| CHR19_11076682_T_G | -0,00599 |
| CHR19_11076648_C_G | -0,00593 |
| CHR19_11086922_G_T | 0 |
| CHR19_44916968_T_TA | 0 |
| CHR19_11079261_T_A | 0 |
| CHR19_11079616_T_C | 0 |
| CHR19_11079805_G_T | 0 |
| CHR19_44909521_CT_C | 0,027283 |
| CHR19_11077488_T_C | 0 |
| CHR19_11078529_C_G | 0 |
| CHR19_11078298_AC_A | 0 |
| CHR19_11077637_C_CTTTA | 0 |
| CHR19_11079398_G_A | 0 |
| CHR19_44906745_G_A | 0 |
| CHR19_11078690_C_CAG | 0 |
| CHR19_11080521_G_A | 0 |
| CHR19_11079304_C_A | 0 |
| CHR19_11079858_G_A | 0 |
| CHR19_11077477_A_C | 0 |
| CHR19_11085680_AC_A | 0 |
| CHR19_11087074_G_GA | 0 |
| CHR19_11086585_G_A | 0 |
| CHR19_11082415_T_G | 0 |
| CHR19_11079873_G_A | 0 |
| CHR19_11077441_C_T | 0 |
| CHR19_11079088_T_C | 0 |
| CHR19_11081053_C_T | 0 |
| CHR19_11078596_T_C | 0 |
| CHR19_44921257_A_C | 0 |
| CHR19_11079868_C_T | 0 |
| CHR19_11080197_C_T | 0 |
| CHR19_11078622_CG_C | 0 |
| CHR19_44912383_G_A | 0 |
| CHR19_11082200_C_CT | 0 |
| CHR19_11079880_T_C | 0 |
| CHR19_11079434_A_G | 0 |
| CHR19_11077868_CT_C | 0 |
| CHR19_11079976_G_A | 0 |
| CHR19_11077571_G_A | 0 |
| CHR2_21063185_T_C | 0,023009 |
| CHR2_21066560_G_A | 0,019625 |
| CHR19_44913574_T_G | 0 |
| CHR2_21065354_G_A | 0,019708 |
| CHR19_11090448_T_C | 0 |
| CHR19_44799247_G_A | -0,00813 |
| CHR5_75278499_A_C | 0,053117 |
| CHR19_11087826_T_C | 0 |
| CHR21_30725132_T_TAC | 0,057156 |
| CHR2_21065449_A_G | 0,007949 |
| CHR19_11091630_G_T | 0 |
| age | 0,200949 |
| bmi | 0,132492 |
| pc0 | -0,00871 |
| pc1 | 0,027623 |
| pc2 | -0,00508 |
| pc3 | 0,001969 |
| pc4 | -0,00106 |
| pc5 | 0,01014 |
| pc6 | -0,00803 |
| pc7 | 0,024793 |
| pc8 | -0,01859 |
| pc9 | -0,02488 |

**Supplementary table S8.** Feature coefficients in the Linear regresssion polygenic score model for HDL-cholesterol levels in the general population sample

| **Predictor** | **Coefficient** |
| --- | --- |
| CHR16_56956804_C_A | 0 |
| CHR16_56957451_C_T | 0.044032 |
| CHR16_56955678_C_T | 0.006654 |
| CHR16_56960332_T_TA | 0.000362 |
| CHR16_56959412_C_A | 0 |
| CHR16_56960616_C_T | 0 |
| CHR16_56953103_C_T | 0 |
| CHR16_56953457_T_C | 0.035255 |
| CHR16_56959249_G_A | 0 |
| CHR16_56953853_C_T | 0 |
| CHR16_56967362_AC_A | 0.018112 |
| CHR16_56954132_C_T | -0.04176 |
| CHR16_56971389_C_T | 0.013622 |
| CHR16_56971567_C_A | 7.09E-05 |
| CHR16_56962376_G_A | 0.003851 |
| CHR16_56962299_G_A | 0.028635 |
| CHR16_56962733_GCC_G | 0 |
| CHR16_56970977_G_A | 0 |
| CHR16_56962737_C_A | 0 |
| CHR16_56967526_G_A | 0 |
| CHR16_56967304_C_T | 0 |
| CHR16_56965416_C_T | 0 |
| CHR16_56962246_T_C | -0.00886 |
| CHR16_56961324_C_A | -0.00385 |
| CHR16_56965006_A_G | -0.02683 |
| CHR16_56968820_T_G | 0.044709 |
| CHR16_56951602_G_A | -0.02192 |
| CHR16_56967342_T_TCACA | 0 |
| CHR16_56966973_G_A | 0 |
| CHR16_56965866_CG_C | 0 |
| CHR16_56966784_T_A | -0.03609 |
| CHR16_56972466_A_G | 0.02465 |
| CHR16_56972678_C_T | 0.004674 |
| CHR16_56965346_A_C | 0 |
| CHR16_56972917_T_A | 0 |
| CHR16_56963437_C_CA | -0.00111 |
| CHR16_56963321_G_A | 0 |
| CHR16_56959997_A_C | 0.00593 |
| CHR16_56968751_C_G | -0.03191 |
| CHR16_56955918_G_A | 0 |
| CHR16_56973534_T_G | -0.00928 |
| CHR16_56966768_CT_C | 0.003301 |
| CHR16_56952850_G_T | -0.00582 |
| CHR16_56967026_G_A | 0 |
| CHR16_56964660_G_C | 0 |
| CHR16_56960982_G_A | -0.00782 |
| CHR16_56957712_G_A | 0 |
| CHR16_56957829_C_T | 0 |
| CHR16_56959299_T_C | 0 |
| CHR16_56951227_A_G | 0 |
| CHR16_56969234_T_C | 0 |
| CHR15_58563549_C_T | 0.001119 |
| CHR16_56951244_A_G | 0 |
| CHR16_56951643_A_G | 0 |
| CHR15_58387985_C_T | 0 |
| CHR15_58387469_G_A | 0 |
| CHR15_58386521_T_C | -0.00359 |
| CHR15_58387608_CAGA_C | 0.009976 |
| CHR16_56960300_T_C | 0 |
| CHR15_58567196_G_A | 0.024248 |
| CHR16_56959113_G_T | 0 |
| CHR15_58387979_T_C | 0 |
| CHR15_58388444_G_T | 0 |
| CHR15_58399949_G_T | 0.013916 |
| CHR15_58382109_G_A | 0 |
| CHR16_56980963_A_G | -0.01216 |
| CHR15_58386313_C_T | 0 |
| CHR8_20002028_G_A | 0.000748 |
| CHR8_19967156_C_T | 0.020561 |
| CHR15_58388440_T_C | 0.005297 |
| CHR15_58388439_T_C | 2.91E-05 |
| CHR18_71032991_C_T | 0.026848 |
| CHR15_58381250_GA_G | -0.00396 |
| CHR15_58391343_G_C | 0 |
| CHR8_19966981_T_C | 0 |
| CHR16_56977273_G_A | 0.004318 |
| CHR18_49656294_A_G | 0.022005 |
| Sex_from_meta_manual | -0.12565 |
| age | 0 |
| bmi | -0.08246 |
| pc0 | -0.00495 |
| pc1 | 0 |
| pc2 | 0 |
| pc3 | 0.000892 |
| pc4 | -0.00421 |
| pc5 | 0 |
| pc6 | 0 |
| pc7 | 0 |
| pc8 | 0.00497 |
| pc9 | 0.000875 |

**Supplementary table S9.** Feature coefficients in the Linear regresssion polygenic score model for LDL-cholesterol levels in the general population sample

| **Predictor** | **Coefficient** |
| --- | --- |
| CHR19_44909976_G_T | -0.09639 |
| CHR19_44908822_C_T | 0 |
| CHR19_44911142_C_A | 0 |
| CHR19_44912383_G_A | 0 |
| CHR19_44913574_T_G | 0 |
| CHR19_44879418_G_A | -0.00557 |
| CHR19_44816374_G_A | 0 |
| CHR19_44897490_T_A | 0 |
| CHR19_44899005_T_G | 0 |
| CHR19_44893972_G_A | 0 |
| CHR19_44886339_G_A | 0 |
| CHR19_44908684_T_C | 0.040297 |
| CHR19_44889415_AT_A | 0 |
| CHR19_44912456_G_A | 0.005241 |
| CHR19_44912678_G_T | 0 |
| CHR19_44799692_GAA_G | 0 |
| CHR19_44923868_T_A | 0 |
| CHR19_44924977_G_A | 0 |
| CHR19_44916968_T_TA | 0 |
| CHR19_44918903_C_G | 0 |
| CHR19_44917997_G_A | 0 |
| CHR19_44919589_G_A | 0 |
| CHR19_44799247_G_A | 0 |
| CHR19_44906745_G_A | 0 |
| CHR19_44921095_A_T | 0 |
| CHR19_44919689_A_G | 0 |
| CHR19_44921257_A_C | 0 |
| CHR19_44921094_A_T | 0 |
| CHR19_44728895_G_A | -0.00538 |
| CHR19_44920730_C_CA | 0.010619 |
| CHR19_44748549_G_T | 0 |
| CHR19_44740135_A_AAT | 0 |
| CHR2_21065354_G_A | 0.014583 |
| CHR19_44943964_G_A | 0 |
| CHR2_21063185_T_C | 0.024919 |
| CHR2_21066560_G_A | 0 |
| CHR19_44883800_A_G | 0 |
| CHR19_44883777_A_C | 0 |
| CHR2_21065449_A_G | 0 |
| CHR2_20951027_T_C | -0.04707 |
| CHR5_75348881_T_G | 0.029272 |
| CHR5_75329662_C_A | 0 |
| CHR5_75348960_T_TA | 0 |
| CHR5_75340659_C_A | 0 |
| CHR5_75330257_T_C | 0 |
| CHR5_75352778_A_T | 0 |
| CHR5_75344665_C_T | 0 |
| CHR5_75343751_C_CAA | 0 |
| CHR5_75341886_A_T | 0 |
| CHR5_75341527_G_GCTTA | 0 |
| CHR5_75359901_C_T | 0 |
| CHR5_75343434_T_G | 0 |
| CHR5_75328657_T_C | 0 |
| CHR5_75278499_A_C | 0 |
| CHR5_75348173_A_ATTC | 0 |
| CHR5_75332550_A_T | 0 |
| CHR2_20993943_C_A | 0 |
| CHR5_75347023_A_T | 0 |
| CHR5_75356084_G_A | 0 |
| CHR5_75321437_G_A | 0 |
| CHR5_75325846_A_G | 0 |
| CHR5_75343353_A_G | 0 |
| CHR19_44888997_C_T | 0 |
| CHR19_11076935_A_G | 0 |
| CHR19_11076682_T_G | 0 |
| CHR5_75279159_A_G | 0 |
| CHR5_75329576_C_CT | 0 |
| CHR19_11087074_G_GA | 0 |
| CHR19_44892362_A_G | 0 |
| CHR19_44892887_C_T | 0 |
| CHR19_11076746_T_C | 0 |
| CHR5_75291041_CT_C | 0 |
| CHR5_75288207_T_C | 0 |
| CHR19_44892652_C_G | 0 |
| CHR19_11076648_C_G | 0 |
| CHR19_44891079_T_C | 0.004881 |
| CHR5_75280460_G_A | 0 |
| CHR5_75339400_G_C | 0 |
| CHR19_11086922_G_T | 0 |
| CHR19_11078690_C_CAG | 0 |
| CHR19_11078174_T_C | 0 |
| CHR19_11079398_G_A | 0 |
| CHR19_11077637_C_CTTTA | 0 |
| CHR19_11078529_C_G | 0 |
| CHR19_11079261_T_A | 0 |
| CHR2_21016795_T_C | 0.000694 |
| CHR19_11079616_T_C | 0 |
| CHR19_11082415_T_G | 0 |
| CHR19_11086585_G_A | 0 |
| CHR19_11081053_C_T | 0 |
| CHR19_11078298_AC_A | 0 |
| CHR5_75306874_C_A | 0 |
| CHR5_75360714_T_C | 0 |
| CHR19_11079805_G_T | 0 |
| CHR19_11079434_A_G | 0 |
| CHR19_11079304_C_A | 0 |
| CHR5_75274031_G_T | 0 |
| CHR5_75315749_G_T | 0 |
| CHR19_11077477_A_C | 0 |
| CHR19_11085680_AC_A | 0 |
| CHR5_75326296_TTA_T | 0 |
| CHR19_11079858_G_A | 0 |
| CHR19_11080521_G_A | 0 |
| CHR19_11079088_T_C | 0 |
| CHR19_11079868_C_T | 0 |
| CHR5_75272410_G_A | 0 |
| CHR19_11078223_C_T | 0 |
| CHR19_11078596_T_C | 0 |
| CHR19_11077441_C_T | 0 |
| CHR19_11078622_CG_C | 0 |
| CHR19_11077488_T_C | 0 |
| CHR19_11090448_T_C | 0 |
| CHR19_11080197_C_T | 0 |
| CHR5_75266739_T_C | 0 |
| CHR19_11079880_T_C | 0 |
| CHR19_11079976_G_A | 0 |
| CHR5_75317068_G_A | 0 |
| CHR19_11079873_G_A | 0 |
| CHR19_11078371_TA_T | 0 |
| CHR5_75355259_A_G | 0 |
| CHR19_44739710_C_T | 0 |
| CHR19_44827846_A_G | 0.008779 |
| CHR19_11077571_G_A | 0 |
| CHR5_75312907_C_T | 0 |
| CHR19_11091630_G_T | 0 |
| CHR5_75307654_G_A | 0 |
| CHR19_44738916_G_A | -0.01143 |
| CHR19_11087826_T_C | 0 |
| CHR19_11091518_GC_G | 0 |
| CHR19_44744370_A_G | 0 |
| CHR19_44884873_G_A | 0 |
| CHR19_45041227_C_CA | 0 |
| CHR19_44884339_G_A | 0 |
| CHR19_44883210_G_GTAA | 0 |
| CHR19_11091312_CHRG | 0 |
| CHR19_44884202_C_G | 0 |
| CHR5_75343719_CTTGTA_C | 0 |
| CHR19_44752422_G_C | 0 |
| CHR5_75308130_C_T | 0 |
| CHR5_75325749_A_AT | 0.003348 |
| CHR19_11077868_CT_C | -0.04566 |
| CHR2_21050024_G_A | 0 |
| CHR2_21051295_A_T | 0 |
| CHR19_44752009_T_C | 0 |
| CHR5_75309395_G_A | 0 |
| CHR5_75319196_C_T | 0 |
| CHR5_75321018_T_C | 0 |
| CHR5_75584065_G_A | 0 |
| CHR19_11082200_C_CT | 0 |
| CHR5_75322361_G_A | 0 |
| CHR19_44865946_G_A | 0 |
| CHR5_75477595_T_C | 0 |
| CHR5_75461731_G_A | 0 |
| CHR5_75491485_T_C | 0 |
| CHR5_75452680_C_T | 0 |
| CHR5_75521119_C_G | 0 |
| CHR5_75472522_C_A | 0 |
| CHR5_75370667_C_T | 0 |
| CHR2_21248731_T_C | 0.020431 |
| CHR19_11082239_A_G | 0 |
| CHR5_75322120_G_A | 0.022004 |
| Sex_from_meta_manual | -0.06198 |
| age | 0.124662 |
| bmi | 0.143427 |
| pc0 | -0.0043 |
| pc1 | 0.023247 |
| pc2 | 0 |
| pc3 | 0 |
| pc4 | 0 |
| pc5 | 0.004646 |
| pc6 | 0 |
| pc7 | 0.007034 |
| pc8 | -0.0157 |
| pc9 | -0.00332 |

**Supplementary table S10.** Feature importance in the polygenic score model for total cholesterol levels in the general population sample

| **Feature** | **Importance** |
| --- | --- |
| CHR19_44909976_G_T | 0.009084 |
| CHR19_44908822_C_T | 0.00771 |
| CHR19_44911142_C_A | 0.001812 |
| CHR19_44879418_G_A | 0.001826 |
| CHR19_44908684_T_C | 0.002942 |
| CHR19_44912383_G_A | 0.001213 |
| CHR19_44913574_T_G | 0.001259 |
| CHR19_44912678_G_T | 0.001672 |
| CHR19_44912456_G_A | 0.001671 |
| CHR19_44906745_G_A | 0.001485 |
| CHR19_44916968_T_TA | 0.000906 |
| CHR19_44923868_T_A | 0.000864 |
| CHR19_44921095_A_T | 0.001013 |
| CHR19_44924977_G_A | 0.000825 |
| CHR19_44917997_G_A | 0.000934 |
| CHR19_44799692_GAA_G | 0.001323 |
| CHR19_44799247_G_A | 0.001081 |
| CHR19_44921094_A_T | 0.002113 |
| CHR19_44919589_G_A | 0.001813 |
| CHR19_44918903_C_G | 0.001793 |
| CHR19_44919689_A_G | 0.00154 |
| CHR19_44816374_G_A | 0.001018 |
| CHR5_75330257_T_C | 0.000126 |
| CHR5_75352778_A_T | 9.5E-05 |
| CHR5_75348960_T_TA | 0.000136 |
| CHR5_75348881_T_G | 0.0002 |
| CHR5_75344665_C_T | 8.69E-05 |
| CHR5_75343751_C_CAA | 0.00012 |
| CHR5_75341527_G_GCTTA | 0.000109 |
| CHR5_75341886_A_T | 0.000104 |
| CHR5_75329662_C_A | 0.0001 |
| CHR5_75359901_C_T | 0.000297 |
| CHR5_75328657_T_C | 0.000153 |
| CHR5_75343434_T_G | 7.65E-05 |
| CHR5_75340659_C_A | 0.00026 |
| CHR5_75332550_A_T | 0.000129 |
| CHR5_75321437_G_A | 0.000358 |
| CHR5_75347023_A_T | 0.000307 |
| CHR5_75348173_A_ATTC | 0.000224 |
| CHR5_75278499_A_C | 0.000687 |
| CHR5_75325846_A_G | 0.000335 |
| CHR5_75356084_G_A | 0.00024 |
| CHR19_44748549_G_T | 0.001196 |
| CHR5_75343353_A_G | 0.000215 |
| CHR5_75306874_C_A | 0.000762 |
| CHR19_44920730_C_CA | 0.001172 |
| CHR5_75360714_T_C | 0.001786 |
| CHR5_75339400_G_C | 0.00022 |
| CHR5_75279159_A_G | 0.000349 |
| CHR5_75315749_G_T | 0.000221 |
| CHR5_75329576_C_CT | 0.00068 |
| CHR5_75355259_A_G | 0.0019 |
| CHR5_75326296_TTA_T | 0.000299 |
| CHR5_75280460_G_A | 0.000326 |
| CHR5_75291041_CT_C | 0.000229 |
| CHR5_75307654_G_A | 0.000416 |
| CHR5_75317068_G_A | 0.000231 |
| CHR5_75288207_T_C | 0.000327 |
| CHR5_75312907_C_T | 0.000195 |
| CHR5_75274031_G_T | 0.000284 |
| CHR5_75266739_T_C | 0.001881 |
| CHR19_44921257_A_C | 0.000997 |
| CHR2_21063185_T_C | 0.001696 |
| CHR5_75272410_G_A | 0.000305 |
| CHR5_75308130_C_T | 0.000932 |
| CHR5_75343719_CTTGTA_C | 0.001146 |
| CHR2_21065354_G_A | 0.001325 |
| CHR5_75491485_T_C | 0.000126 |
| CHR19_44892362_A_G | 0.000626 |
| CHR5_75472522_C_A | 0.000162 |
| CHR5_75461731_G_A | 0.000127 |
| CHR5_75370667_C_T | 0.000274 |
| CHR5_75513055_C_T | 0.000221 |
| CHR5_75584065_G_A | 0.000314 |
| CHR2_21065449_A_G | 0.001817 |
| CHR19_44892652_C_G | 0.000608 |
| CHR5_75452680_C_T | 0.000123 |
| CHR2_21066560_G_A | 0.00131 |
| CHR5_75477595_T_C | 0.000204 |
| CHR5_75521119_C_G | 8.84E-05 |
| CHR19_44943964_G_A | 0.00038 |
| CHR19_44892887_C_T | 0.0007 |
| CHR11_116830819_T_C | 0.005446 |
| CHR5_75463358_T_A | 7.78E-05 |
| CHR5_75393424_T_C | 0.000146 |
| CHR5_75581978_T_C | 0.000198 |
| CHR19_44888997_C_T | 0.001749 |
| CHR5_75614147_C_T | 0.000336 |
| CHR5_75615045_A_G | 0.000147 |
| CHR5_75530910_G_A | 8.06E-05 |
| CHR5_75440775_TA_T | 0.000993 |
| CHR5_75319196_C_T | 0.000641 |
| CHR5_75512412_G_T | 8.46E-05 |
| CHR2_20967152_C_T | 0.001466 |
| CHR5_75321018_T_C | 0.000607 |
| CHR5_75322361_G_A | 0.000669 |
| CHR5_75524614_G_GA | 0.000289 |
| CHR19_44884339_G_A | 0.000529 |
| CHR5_75309395_G_A | 0.000787 |
| CHR5_75611138_A_C | 0.000798 |
| CHR19_44883210_G_GTAA | 0.000656 |
| CHR19_44884202_C_G | 0.000501 |
| CHR6_61022872_G_C | 0.000465 |
| CHR5_75292432_G_A | 0.001209 |
| CHR5_75322120_G_A | 0.00065 |
| CHR5_75299369_A_C | 0.00145 |
| CHR19_11076935_A_G | 0.000304 |
| CHR19_44891079_T_C | 0.000955 |
| CHR3_82771751_T_G | 0.015208 |
| CHR19_11077571_G_A | 4.88E-05 |
| CHR19_11076682_T_G | 0.000208 |
| CHR19_11076746_T_C | 0.000178 |
| CHR19_11076648_C_G | 0.000188 |
| CHR19_11078174_T_C | 0.000288 |
| CHR5_75279976_A_G | 0.001207 |
| CHR19_11086922_G_T | 0.000148 |
| CHR19_11087074_G_GA | 0.000357 |
| CHR19_11079261_T_A | 5.1E-05 |
| CHR5_75323814_C_T | 0.000615 |
| CHR5_75292115_C_A | 0.001116 |
| CHR19_11079616_T_C | 6.33E-05 |
| CHR19_11078529_C_G | 6.25E-05 |
| CHR19_11079398_G_A | 5.93E-05 |
| CHR19_11077488_T_C | 5.19E-05 |
| CHR19_11077637_C_CTTTA | 4.73E-05 |
| CHR19_11079304_C_A | 0.00017 |
| CHR19_11079434_A_G | 5.59E-05 |
| CHR19_11081053_C_T | 7.49E-05 |
| CHR19_11077441_C_T | 5.1E-05 |
| CHR19_11080521_G_A | 5.94E-05 |
| CHR19_11079858_G_A | 6.84E-05 |
| CHR19_11079805_G_T | 5.23E-05 |
| CHR19_11078690_C_CAG | 6.02E-05 |
| CHR19_44884873_G_A | 0.000791 |
| CHR19_11077477_A_C | 4.8E-05 |
| CHR19_11086585_G_A | 0.00013 |
| CHR19_11078298_AC_A | 8.04E-05 |
| CHR19_11082415_T_G | 6.72E-05 |
| CHR19_44886339_G_A | 0.00073 |
| CHR19_11078596_T_C | 6.84E-05 |
| CHR19_11079088_T_C | 5.96E-05 |
| CHR19_11085680_AC_A | 0.000132 |
| CHR19_11080197_C_T | 6.21E-05 |
| CHR19_11079868_C_T | 5.84E-05 |
| CHR19_11090448_T_C | 0.000126 |
| CHR19_11079873_G_A | 8.03E-05 |
| CHR19_11078622_CG_C | 4.92E-05 |
| CHR19_11079976_G_A | 6.84E-05 |
| CHR19_11079880_T_C | 4.54E-05 |
| CHR5_75308917_G_A | 0.00188 |
| CHR5_75638184_A_T | 0.002175 |
| CHR5_75276089_G_A | 0.000489 |
| CHR19_11078223_C_T | 6.43E-05 |
| CHR19_44728895_G_A | 0.000495 |
| CHR19_11078371_TA_T | 6.11E-05 |
| CHR19_11087826_T_C | 0.000171 |
| CHR2_20951027_T_C | 0.004636 |
| CHR19_11091630_G_T | 0.000198 |
| CHR5_75274706_G_A | 0.000512 |
| CHR5_75273532_C_T | 0.000463 |
| CHR19_11091518_GC_G | 0.000248 |
| CHR22_16056713_G_T | 0 |
| CHR19_11091312_CHRG | 0.000266 |
| CHR5_75268365_A_G | 0.000483 |
| CHR19_11082200_C_CT | 0.00023 |
| CHR19_44897490_T_A | 0.000477 |
| CHR5_75269328_C_T | 0.000462 |
| CHR19_44893972_G_A | 0.000464 |
| CHR19_44892587_G_A | 0.001561 |
| CHR19_44537330_C_T | 0.000987 |
| CHR2_20993943_C_A | 0.005217 |
| CHR19_11080525_A_AG | 0.000103 |
| CHR19_11082239_A_G | 9.27E-05 |
| CHR19_44899005_T_G | 0.000451 |
| CHR19_11080624_G_A | 0.000109 |
| CHR19_11077868_CT_C | 0.00061 |
| CHR19_11087511_C_T | 0.000253 |
| CHR19_11081001_T_A | 9.02E-05 |
| CHR5_75323307_C_T | 0.001195 |
| CHR19_11079781_GAAAA_G | 0.000184 |
| CHR19_11082155_A_G | 8.19E-05 |
| CHR19_11086210_T_C | 0.000327 |
| CHR19_11081874_G_A | 7.9E-05 |
| CHR19_11083273_A_G | 8.59E-05 |
| CHR19_11081517_A_T | 6.6E-05 |
| CHR3_12369449_G_A | 0.001411 |
| CHR11_116962939_T_G | 0.000196 |
| CHR5_75293560_C_T | 0.000946 |
| CHR5_75325749_A_AT | 0.001298 |
| CHR5_162184790_TTTGA_T | 0.000139 |
| CHR5_162193934_G_T | 0.000538 |
| CHR19_44740135_A_AAT | 0.000404 |
| CHR5_162183783_G_A | 0.000432 |
| CHR22_16057055_G_A | 0 |
| CHR2_21199531_G_A | 0.000157 |
| CHR5_162119815_T_C | 0.0017 |
| CHR11_117124277_CA_C | 0.000707 |
| CHR7_76939283_T_C | 0.001134 |
| CHR11_54748602_A_G | 0.000992 |
| CHR11_116786874_C_CA | 0.000259 |
| CHR2_21203871_C_A | 0.000229 |
| CHR17_56343341_G_T | 0.001772 |
| CHR17_56341365_A_G | 0.001673 |
| CHR2_21201117_T_C | 0.000207 |
| CHR2_21198910_C_A | 0.000208 |
| CHR5_162203665_G_A | 0.000241 |
| CHR7_77057349_T_A | 0.001862 |
| CHR2_21200056_C_T | 0.000257 |
| CHR5_162188534_T_C | 8.65E-05 |
| CHR2_21202451_A_G | 0.000205 |
| CHR5_162193048_CACT_C | 7.89E-05 |
| CHR11_116982292_C_CT | 0.001584 |
| CHR7_76949154_T_C | 0.001159 |
| CHR7_76940458_A_G | 0.00104 |
| CHR5_162167796_A_G | 0.000205 |
| CHR2_21196937_A_T | 0.00017 |
| CHR2_21197825_A_G | 0.000157 |
| CHR2_21195884_A_G | 0.000173 |
| CHR2_21196448_C_G | 0.000166 |
| CHR2_21197486_C_A | 0.000173 |
| CHR2_21195847_C_A | 0.000193 |
| CHR5_75272020_C_A | 0.000442 |
| CHR5_162187628_G_A | 0.000115 |
| CHR2_21194704_C_T | 0.000165 |
| CHR5_162144725_G_T | 0.00018 |
| CHR12_130148997_G_A | 0.002678 |
| CHR5_162184251_T_A | 0.000142 |
| CHR19_44889415_AT_A | 0.00112 |
| CHR11_117115968_C_A | 0.000388 |
| CHR5_162128192_C_T | 0.000161 |
| CHR5_162138704_G_A | 0.000156 |
| CHR5_162133126_C_G | 0.000274 |
| CHR17_56332110_A_G | 0.001514 |
| CHR5_162134330_ATACT_A | 0.000138 |
| CHR5_162164541_A_T | 0.000234 |
| CHR5_162123232_G_C | 0.00015 |
| CHR3_12281910_C_T | 0.000173 |
| CHR11_116951202_T_G | 7.92E-05 |
| CHR5_162178804_T_G | 0.000205 |
| CHR2_21195211_G_A | 0.000125 |
| CHR11_116939229_C_A | 4.34E-05 |
| CHR11_117116626_G_C | 0.000398 |
| CHR3_12304609_A_T | 0.000223 |
| CHR9_43185585_G_T | 0 |
| CHR5_162203133_C_T | 0.000149 |
| CHR5_162128617_C_G | 0.000125 |
| CHR5_162169920_C_T | 0.000119 |
| CHR11_116931095_C_T | 6.38E-05 |
| CHR11_116926139_T_A | 5.71E-05 |
| CHR11_117011519_AT_A | 0.000183 |
| CHR5_162141284_T_G | 0.001433 |
| CHR5_162202969_T_C | 0.000116 |
| CHR11_116964071_C_T | 0.000132 |
| CHR11_116784884_G_A | 0.000413 |
| CHR17_56344133_A_G | 0.001532 |
| CHR5_162146486_C_G | 0.000148 |
| CHR22_16057202_G_A | 7.79E-05 |
| CHR11_116999579_G_GTTTTC | 0.00017 |
| CHR5_162172999_GA_G | 0.000184 |
| CHR11_116945914_C_G | 8.17E-05 |
| CHR5_75200911_C_CAA | 0.002373 |
| CHR11_116804102_A_G | 0.005485 |
| CHR11_116931865_T_A | 4.39E-05 |
| CHR3_12301362_G_T | 0.000166 |
| CHR5_162175442_C_G | 0.000153 |
| CHR3_12130131_A_AGT | 0.000748 |
| CHR11_116921641_T_G | 0.000572 |
| CHR5_162123420_A_G | 7.82E-05 |
| CHR20_29729529_C_G | 0.000264 |
| CHR11_116968819_T_C | 3.55E-05 |
| CHR11_116778822_A_G | 0.000316 |
| CHR5_162175044_A_G | 9.06E-05 |
| CHR11_116964069_C_T | 3.77E-05 |
| CHR22_16057195_T_A | 8.36E-05 |
| CHR11_116949116_G_GA | 8.96E-05 |
| CHR3_12302050_C_A | 0.000149 |
| CHR9_61729097_T_TA | 0.002283 |
| CHR11_116965315_G_A | 4.12E-05 |
| CHR5_162178602_C_T | 0.000237 |
| CHR11_116962339_G_A | 5.63E-05 |
| CHR5_75117888_T_C | 0.000496 |
| CHR5_162144694_A_G | 0.000106 |
| CHR11_117109878_C_T | 0.000343 |
| CHR11_116965493_A_C | 4.48E-05 |
| CHR11_117112212_G_A | 0.000379 |
| CHR5_162167510_G_A | 0.000172 |
| CHR3_12288912_G_A | 0.00058 |
| CHR5_162175318_T_A | 0.000121 |
| CHR11_116948525_C_T | 8.17E-05 |
| CHR5_162145612_T_G | 9.19E-05 |
| CHR3_12300202_CAG_C | 0.0002 |
| CHR5_162161438_T_G | 8.79E-05 |
| CHR19_44881148_T_C | 0.001852 |
| CHR19_11085975_AT_A | 0.001092 |
| CHR5_162163333_G_A | 9.87E-05 |
| CHR11_116970730_A_T | 3.23E-05 |
| CHR5_75232565_GTT_G | 0.001943 |
| CHR11_116953280_A_T | 4.37E-05 |
| CHR3_12363981_G_A | 0.000274 |
| CHR5_162160747_T_G | 6.83E-05 |
| CHR5_162125405_A_T | 0.000106 |
| CHR11_116951318_C_T | 4.87E-05 |
| CHR5_162130471_A_G | 7.46E-05 |
| CHR9_43186235_A_T | 0.000201 |
| CHR5_162130947_A_C | 0.000114 |
| CHR11_117001547_A_G | 7.51E-05 |
| CHR11_117117597_A_G | 0.00045 |
| CHR11_117014597_TC_T | 5.41E-05 |
| CHR3_12368473_C_T | 0.000322 |
| CHR2_21011100_T_C | 0.000845 |
| CHR11_117000507_A_G | 9.39E-05 |
| CHR11_116961196_C_T | 0.000116 |
| CHR11_116791615_G_T | 0.000393 |
| CHR5_162159721_C_T | 0.000108 |
| CHR5_162159663_C_T | 8.62E-05 |
| CHR11_116767187_CA_C | 0.000322 |
| CHR5_162161379_G_A | 8.66E-05 |
| CHR11_117006637_T_C | 6.61E-05 |
| CHR11_116949355_T_A | 0.00013 |
| CHR19_44747899_A_C | 0.003219 |
| CHR3_12309724_A_G | 0.000287 |
| CHR5_162142083_A_G | 8.27E-05 |
| CHR11_117012680_T_C | 3.69E-05 |
| CHR5_162127399_T_C | 8.65E-05 |
| CHR11_7174277_C_T | 0.005735 |
| CHR5_162158954_A_C | 0.000112 |
| CHR11_117013061_G_T | 5.3E-05 |
| CHR5_162159668_G_C | 9.21E-05 |
| CHR9_43186377_G_A | 9.64E-07 |
| CHR5_75099216_T_C | 0.00045 |
| CHR3_12299426_T_C | 0.000194 |
| CHR3_12332833_C_G | 0.000152 |
| CHR11_117013060_A_T | 4.86E-05 |
| CHR3_127456657_G_A | 0.001697 |
| CHR11_117109906_G_A | 0.000438 |
| CHR11_116997938_C_A | 3.88E-05 |
| CHR11_117009987_A_G | 4.49E-05 |
| CHR3_12333144_A_AAAAC | 0.000253 |
| CHR11_117110070_G_C | 0.000425 |
| CHR5_75065315_T_C | 0.000483 |
| CHR16_50244934_TG_T | 0.002206 |
| CHR3_12397178_T_A | 0.000238 |
| CHR7_76975262_TGCCC_T | 0.000903 |
| CHR2_21248731_T_C | 0.00244 |
| CHR10_110619065_C_T | 0.005085 |
| CHR11_116947068_C_T | 0.000419 |
| CHR10_110614544_C_T | 0.004352 |
| CHR11_116991428_T_C | 5.43E-05 |
| CHR3_12408294_C_A | 0.000221 |
| CHR3_12415190_C_T | 0.000329 |
| CHR5_75097057_C_T | 0.000379 |
| CHR19_44693694_G_A | 0.000644 |
| CHR5_162159208_A_G | 7.66E-05 |
| CHR2_20952995_G_A | 0.000638 |
| CHR11_116766430_C_T | 0.000331 |
| CHR5_75104691_G_C | 0.000364 |
| CHR16_50245142_T_C | 0.002088 |
| CHR5_75076316_T_C | 0.000374 |
| CHR5_75112166_C_T | 0.000407 |
| CHR2_21196630_T_TAC | 0.000574 |
| CHR3_12396637_G_A | 0.000343 |
| CHR11_116993117_C_T | 5.88E-05 |
| CHR8_125473095_A_G | 0.001025 |
| CHR20_35752656_C_CA | 0.003623 |
| CHR5_75080833_G_C | 0.00041 |
| CHR19_45041227_C_CA | 0.000758 |
| CHR19_44862466_TA_T | 0.001936 |
| CHR11_116779402_C_T | 0.000154 |
| CHR8_125466108_A_C | 0.000932 |
| CHR11_116787315_T_G | 0.000253 |
| CHR3_12389797_T_C | 0.000342 |
| CHR3_12334928_A_G | 0.000153 |
| CHR11_116791691_G_C | 0.000562 |
| CHR7_77069554_T_C | 0.001633 |
| CHR2_21051295_A_T | 0.001408 |
| CHR5_75087344_T_G | 0.000468 |
| CHR19_44743791_C_T | 0.003275 |
| CHR8_125473052_T_A | 0.001042 |
| CHR11_116870837_G_A | 0.000713 |
| CHR11_116781491_C_T | 0.000161 |
| CHR3_12393402_C_T | 0.000262 |
| CHR2_21050024_G_A | 0.001407 |
| CHR9_43187431_C_T | 0 |
| CHR5_75128542_C_T | 0.001513 |
| CHR8_125475993_C_T | 0.000838 |
| CHR3_12407495_T_C | 0.000268 |
| CHR8_125467073_G_A | 0.000828 |
| CHR8_125467846_G_A | 0.000883 |
| CHR11_117097552_C_T | 0.000462 |
| CHR5_75085132_A_G | 0.000563 |
| CHR8_125466503_A_G | 0.000869 |
| CHR5_75085134_A_G | 0.000876 |
| CHR11_116734354_C_T | 0.000452 |
| CHR8_42796887_A_C | 0.004885 |
| CHR16_13363582_G_A | 0.001335 |
| CHR3_12334081_C_T | 0.00016 |
| CHR11_116781635_A_G | 0.000174 |
| CHR3_127455318_T_C | 0.001792 |
| CHR2_21162277_T_C | 0.000657 |
| CHR5_75155913_CT_C | 0.00161 |
| CHR11_116733733_AGGCTGG_A | 0.000382 |
| CHR8_125474167_A_G | 0.001152 |
| CHR8_125468458_A_G | 0.000883 |
| CHR8_125469505_A_G | 0.000812 |
| CHR16_13363366_A_G | 0.001339 |
| CHR13_106199807_C_T | 0.003344 |
| CHR5_75220642_CA_C | 0.00199 |
| CHR2_21190866_G_A | 0.000467 |
| CHR5_75396951_A_G | 0.002561 |
| CHR7_77066199_T_G | 0.001408 |
| CHR3_12271009_C_T | 0.000247 |
| CHR11_117120044_G_GAAAC | 0.00362 |
| CHR2_10332238_A_T | 0.001764 |
| CHR16_13363112_A_G | 0.001326 |
| CHR4_79163346_C_G | 0.003887 |
| CHR11_116855332_C_A | 0.000862 |
| CHR11_116778419_G_A | 0.000161 |
| CHR3_12402159_G_T | 0.000281 |
| CHR3_12245673_C_T | 0.000223 |
| CHR3_127460304_A_G | 0.001877 |
| CHR16_13363288_G_A | 0.001375 |
| CHR22_16057053_T_G | 0 |
| CHR8_125476008_C_T | 0.000839 |
| CHR11_117120135_A_G | 0.003368 |
| CHR5_75434186_G_GT | 0.002331 |
| CHR11_116733316_T_C | 0.000372 |
| CHR3_127457046_T_C | 0.00164 |
| CHR5_31757682_C_T | 0.002118 |
| CHR2_21016795_T_C | 0.003888 |
| CHR2_184283555_G_A | 0.00061 |
| CHR13_58145235_A_C | 0.004346 |
| CHR11_116856749_C_T | 0.000602 |
| CHR8_125470379_C_A | 0.000872 |
| CHR3_127455086_A_G | 0.001737 |
| CHR13_106201597_G_A | 0.00324 |
| CHR2_21032892_C_T | 0.000954 |
| CHR5_75083850_T_A | 0.001445 |
| CHR7_77068214_T_C | 0.001486 |
| CHR3_12245546_T_G | 0.000232 |
| CHR11_116734360_G_A | 0.00036 |
| CHR11_116848213_G_T | 0.000774 |
| CHR5_75548537_G_A | 0.002659 |
| CHR16_50247760_G_A | 0.002923 |
| CHR12_130153145_G_A | 0.001739 |
| Sex_from_meta_manual | 0.085614 |
| age | 0.090348 |
| bmi | 0.187599 |
| pc0 | 0.035753 |
| pc1 | 0.030774 |
| pc2 | 0.024086 |
| pc3 | 0.025923 |
| pc4 | 0.026815 |
| pc5 | 0.02848 |
| pc6 | 0.026851 |
| pc7 | 0.028229 |
| pc8 | 0.028588 |
| pc9 | 0.030164 |

**Supplementary table S11.** Feature importance in the polygenic score model for total cholesterol levels in men

| **Feature** | **Importance** |
| --- | --- |
| CHR19_44909976_G_T | 0.004391 |
| CHR19_44908822_C_T | 0.00386 |
| CHR11_42702551_T_C | 0.003343 |
| CHR19_44911142_C_A | 0.005905 |
| CHR11_54748602_A_G | 0.00403 |
| CHR11_42744311_G_GA | 0.001185 |
| CHR5_16841935_T_G | 0.009953 |
| CHR12_109256961_T_C | 0.005785 |
| CHR11_42744240_G_A | 0.001004 |
| CHR5_6473277_AC_A | 0.01028 |
| CHR11_42744218_A_G | 0.001053 |
| CHR12_109255543_G_A | 0.005904 |
| CHR11_48813072_C_G | 0.0026 |
| CHR11_42744121_G_T | 0.001114 |
| CHR11_42744633_A_G | 0.001136 |
| CHR11_42745962_A_G | 0.000996 |
| CHR11_42744391_G_A | 0.001113 |
| CHR3_3380082_A_G | 0.00361 |
| CHR11_116830819_T_C | 0.010775 |
| CHR19_44912383_G_A | 0.004397 |
| CHR19_44913574_T_G | 0.004467 |
| CHR1_109273124_A_G | 0.002522 |
| CHR11_54748607_T_G | 0.003769 |
| CHR11_42744307_T_TTC | 0.001071 |
| CHR2_20967152_C_T | 0.003281 |
| CHR1_90670307_A_C | 0.003652 |
| CHR1_90670938_A_G | 0.003693 |
| CHR1_109272589_C_A | 0.002672 |
| CHR6_124075586_T_G | 0.002423 |
| CHR6_61022872_G_C | 0.00239 |
| CHR6_124074142_C_T | 0.002514 |
| CHR6_124075032_T_G | 0.002432 |
| CHR1_90696169_T_C | 0.001159 |
| CHR12_40627202_TA_T | 0.003572 |
| CHR1_90667647_C_T | 0.001146 |
| CHR11_485086_C_CTG | 0.007858 |
| CHR1_90696212_G_A | 0.001184 |
| CHR1_90694029_T_C | 0.001065 |
| CHR1_90678498_T_C | 0.001254 |
| CHR3_3382734_T_G | 0.003421 |
| CHR1_90696496_T_C | 0.00119 |
| CHR1_90664497_T_C | 0.001067 |
| CHR1_109272746_T_C | 0.002665 |
| CHR14_63590232_G_T | 0.007895 |
| CHR1_90670660_T_C | 0.003562 |
| CHR3_3377075_G_T | 0.005102 |
| CHR1_90677429_T_C | 0.001014 |
| CHR3_3382548_A_G | 0.003218 |
| CHR1_90662056_C_CA | 0.003166 |
| CHR1_90698570_G_T | 0.001057 |
| CHR6_124074155_T_C | 0.002492 |
| CHR1_90701982_CT_C | 0.001209 |
| CHR2_204522432_C_A | 0.007182 |
| CHR1_90662481_G_A | 0.001099 |
| CHR1_90658106_T_G | 0.002773 |
| CHR9_92552190_TAC_T | 0.00409 |
| CHR5_75315408_G_T | 0.01059 |
| CHR2_91933274_C_T | 0.002613 |
| CHR1_19076679_C_T | 0.004446 |
| CHR1_90686913_G_T | 0.00109 |
| CHR19_7308862_T_A | 0.007733 |
| CHR3_3380195_G_T | 0.002835 |
| CHR2_214940439_T_G | 0.007066 |
| CHR3_3382648_C_A | 0.004387 |
| CHR12_114023417_A_C | 0.003459 |
| CHR20_11081373_A_C | 0.006918 |
| CHR6_72325416_T_C | 0.003056 |
| CHR9_81715347_CA_C | 0.010308 |
| CHR3_3380423_C_T | 0.003166 |
| CHR1_109272511_G_A | 0.002838 |
| CHR12_114024939_G_C | 0.003527 |
| CHR16_12736607_C_T | 0.003463 |
| CHR5_163429761_G_A | 0.005036 |
| CHR12_114023754_G_GT | 0.003595 |
| CHR1_19154483_G_A | 0.003368 |
| CHR6_52264411_T_G | 0.006649 |
| CHR6_30986961_G_A | 0.008617 |
| CHR11_116770056_C_CAA | 0.008773 |
| CHR2_20951027_T_C | 0.008744 |
| CHR1_90685900_G_A | 0.001066 |
| CHR16_12739559_C_G | 0.003549 |
| CHR2_7976831_G_A | 0.0067 |
| CHR12_114021567_A_G | 0.003337 |
| CHR10_130623353_C_A | 0.003141 |
| CHR1_19273912_C_T | 0.003434 |
| CHR3_3382270_C_T | 0.0043 |
| CHR4_77969558_TA_T | 0.005986 |
| CHR5_163350156_T_A | 0.006573 |
| CHR8_127459937_A_C | 0.009133 |
| CHR2_123570834_G_T | 0.002953 |
| CHR2_21011100_T_C | 0.0029 |
| age | 0.047127 |
| bmi | 0.161635 |
| pc0 | 0.057187 |
| pc1 | 0.042996 |
| pc2 | 0.036918 |
| pc3 | 0.044875 |
| pc4 | 0.041212 |
| pc5 | 0.04624 |
| pc6 | 0.037331 |
| pc7 | 0.040248 |
| pc8 | 0.04452 |
| pc9 | 0.037604 |

**Supplementary table S12.** Feature importance in the polygenic score model for total cholesterol levels in women

| **Predictor** | **Coefficient** |
| --- | --- |
| CHR19_44908822_C_T | 0.006686 |
| CHR19_44909976_G_T | 0.005635 |
| CHR19_44879418_G_A | 0.003393 |
| CHR19_44816374_G_A | 0.001626 |
| CHR19_44921094_A_T | 0.002906 |
| CHR19_44918903_C_G | 0.002703 |
| CHR19_44919589_G_A | 0.002749 |
| CHR19_44919689_A_G | 0.00276 |
| CHR19_44921095_A_T | 0.002117 |
| CHR19_44924977_G_A | 0.001856 |
| CHR19_44917997_G_A | 0.002281 |
| CHR19_44923868_T_A | 0.001988 |
| CHR19_44908684_T_C | 0.003623 |
| CHR19_44911142_C_A | 0.001684 |
| CHR19_44912678_G_T | 0.002234 |
| CHR19_44912456_G_A | 0.002214 |
| CHR19_44920730_C_CA | 0.002501 |
| CHR19_11076935_A_G | 0.000544 |
| CHR19_11076746_T_C | 0.000321 |
| CHR19_11078174_T_C | 7.83E-05 |
| CHR19_11076682_T_G | 0.000414 |
| CHR19_11076648_C_G | 0.000326 |
| CHR19_11086922_G_T | 0.000314 |
| CHR19_44916968_T_TA | 0.000863 |
| CHR19_11079616_T_C | 9.46E-05 |
| CHR19_11079261_T_A | 6.84E-05 |
| CHR19_11079805_G_T | 7.34E-05 |
| CHR19_44909521_CT_C | 0.00302 |
| CHR19_11077488_T_C | 7.61E-05 |
| CHR19_11078529_C_G | 0.000107 |
| CHR19_11078298_AC_A | 0.000119 |
| CHR19_11077637_C_CTTTA | 9.37E-05 |
| CHR19_11079398_G_A | 0.0001 |
| CHR19_44906745_G_A | 0.002489 |
| CHR19_11078690_C_CAG | 0.000111 |
| CHR19_11077477_A_C | 7.11E-05 |
| CHR19_11079858_G_A | 8.33E-05 |
| CHR19_11080521_G_A | 8.09E-05 |
| CHR19_11079304_C_A | 7.95E-05 |
| CHR19_11085680_AC_A | 0.000249 |
| CHR19_11087074_G_GA | 0.000266 |
| CHR19_11086585_G_A | 0.000144 |
| CHR19_11082415_T_G | 0.000111 |
| CHR19_11079873_G_A | 0.000116 |
| CHR19_11077441_C_T | 8.46E-05 |
| CHR19_11079088_T_C | 0.00011 |
| CHR19_11081053_C_T | 9.49E-05 |
| CHR19_11078596_T_C | 9.01E-05 |
| CHR19_44921257_A_C | 0.002148 |
| CHR19_11079868_C_T | 9.37E-05 |
| CHR19_11080197_C_T | 0.000119 |
| CHR19_11078622_CG_C | 6.82E-05 |
| CHR19_44912383_G_A | 0.001641 |
| CHR19_11082200_C_CT | 0.000214 |
| CHR19_11079880_T_C | 6.71E-05 |
| CHR19_11079434_A_G | 0.000111 |
| CHR19_11077868_CT_C | 0.001002 |
| CHR19_11079976_G_A | 9.95E-05 |
| CHR19_11077571_G_A | 8.61E-05 |
| CHR2_21063185_T_C | 0.004523 |
| CHR2_21066560_G_A | 0.004129 |
| CHR19_44913574_T_G | 0.001675 |
| CHR2_21065354_G_A | 0.004242 |
| CHR19_11090448_T_C | 0.000139 |
| CHR19_44799247_G_A | 0.000575 |
| CHR5_75278499_A_C | 0.00118 |
| CHR19_11087826_T_C | 0.000136 |
| CHR21_30725132_T_TAC | 0.001186 |
| CHR2_21065449_A_G | 0.004464 |
| CHR19_11091630_G_T | 0.000292 |
| CHR19_11091518_GC_G | 0.000272 |
| CHR19_11082239_A_G | 0.000166 |
| CHR19_11086210_T_C | 0.000448 |
| CHR19_11078371_TA_T | 0.000101 |
| CHR5_75306874_C_A | 0.001549 |
| CHR19_11091312_CHRG | 0.000323 |
| CHR19_44799692_GAA_G | 0.000791 |
| CHR19_11081001_T_A | 0.000212 |
| CHR19_11080624_G_A | 0.000153 |
| CHR19_11080525_A_AG | 0.000178 |
| CHR19_11081517_A_T | 0.00016 |
| CHR19_11081874_G_A | 0.000183 |
| CHR19_11083273_A_G | 0.000168 |
| CHR19_11082155_A_G | 0.00017 |
| CHR19_11087511_C_T | 0.000329 |
| CHR19_11097068_CTT_C | 0.001408 |
| CHR5_75328657_T_C | 0.000557 |
| CHR5_75329662_C_A | 0.000495 |
| CHR5_75330257_T_C | 0.000468 |
| CHR5_75279159_A_G | 0.000963 |
| CHR5_75321437_G_A | 0.00113 |
| CHR5_75325846_A_G | 0.001192 |
| CHR5_75340659_C_A | 0.000646 |
| CHR19_11078223_C_T | 0.000128 |
| CHR3_14529300_AAAG_A | 0.001155 |
| CHR5_75341527_G_GCTTA | 0.00051 |
| CHR5_75352778_A_T | 0.000388 |
| CHR5_75280460_G_A | 0.001043 |
| CHR5_75288207_T_C | 0.000974 |
| CHR19_11079781_GAAAA_G | 0.000219 |
| CHR19_44862466_TA_T | 0.002777 |
| CHR5_75344665_C_T | 0.000442 |
| CHR5_75291041_CT_C | 0.000944 |
| CHR5_75308130_C_T | 0.001761 |
| CHR5_75274031_G_T | 0.001147 |
| CHR5_75348960_T_TA | 0.000473 |
| CHR5_75341886_A_T | 0.000443 |
| CHR5_75348881_T_G | 0.000557 |
| CHR19_44748549_G_T | 0.000998 |
| CHR5_75343751_C_CAA | 0.000377 |
| CHR5_75307654_G_A | 0.00078 |
| CHR5_75332550_A_T | 0.000503 |
| CHR5_75343434_T_G | 0.000411 |
| CHR5_75359901_C_T | 0.000591 |
| CHR5_75272410_G_A | 0.000972 |
| CHR5_75266739_T_C | 0.00278 |
| CHR5_75348173_A_ATTC | 0.000521 |
| CHR19_44858389_A_G | 0.00271 |
| CHR5_75360714_T_C | 0.002676 |
| CHR19_44943964_G_A | 0.001852 |
| CHR5_75270348_T_C | 0.001064 |
| CHR1_219392044_G_A | 0.000977 |
| CHR21_30689613_A_G | 0.000778 |
| CHR5_75315749_G_T | 0.00069 |
| CHR1_107534097_T_C | 0.004314 |
| CHR5_75317068_G_A | 0.000742 |
| CHR19_11085975_AT_A | 0.001114 |
| CHR14_62948858_C_T | 0.000668 |
| CHR1_219199344_G_A | 0.001042 |
| CHR5_75356084_G_A | 0.000543 |
| CHR19_44881148_T_C | 0.004985 |
| CHR5_75347023_A_T | 0.000724 |
| CHR5_75343719_CTTGTA_C | 0.002601 |
| CHR6_73374589_T_G | 0.002493 |
| CHR1_107549780_CT_C | 0.004502 |
| CHR5_75329576_C_CT | 0.001328 |
| CHR5_75343353_A_G | 0.000588 |
| CHR21_30676393_T_C | 0.000677 |
| CHR20_29729529_C_G | 0.000626 |
| CHR1_107534520_A_G | 0.00437 |
| CHR5_75355259_A_G | 0.002805 |
| CHR5_75312907_C_T | 0.00064 |
| CHR19_44537330_C_T | 0.001209 |
| CHR1_107533327_G_A | 0.004267 |
| CHR3_189736784_TAAG_T | 0.011706 |
| CHR5_75524614_G_GA | 0.001001 |
| CHR5_75440775_TA_T | 0.001828 |
| CHR9_30312433_CT_C | 0.004788 |
| CHR19_44888997_C_T | 0.007396 |
| CHR15_32902073_C_T | 0.002931 |
| CHR5_75339400_G_C | 0.000519 |
| CHR19_44728895_G_A | 0.00073 |
| CHR5_75326296_TTA_T | 0.000806 |
| CHR5_75584065_G_A | 0.001084 |
| CHR1_157075636_CTT_C | 0.001889 |
| CHR14_75720349_A_C | 0.00402 |
| CHR5_75472522_C_A | 0.000806 |
| CHR5_75513055_C_T | 0.000755 |
| CHR19_11076509_A_AT | 0.001053 |
| CHR5_75614147_C_T | 0.001245 |
| CHR5_75370667_C_T | 0.000661 |
| CHR5_75452680_C_T | 0.000593 |
| CHR5_75491485_T_C | 0.000627 |
| CHR11_79830315_T_A | 0.002649 |
| CHR7_35455797_C_T | 0.003343 |
| CHR1_107538306_G_A | 0.003871 |
| CHR1_107549350_C_T | 0.003692 |
| CHR5_75521119_C_G | 0.000659 |
| CHR5_75477595_T_C | 0.000639 |
| CHR19_11083117_A_AT | 0.001651 |
| CHR5_75393424_T_C | 0.000628 |
| CHR7_14089487_C_T | 0.004284 |
| CHR7_14071628_C_T | 0.002898 |
| CHR7_35456847_G_A | 0.002931 |
| CHR11_79827765_C_T | 0.002598 |
| CHR7_75666881_G_A | 0.002072 |
| CHR5_75463358_T_A | 0.000606 |
| CHR4_103732725_G_A | 0.00651 |
| CHR6_73358532_A_AC | 0.002118 |
| CHR3_6328378_T_A | 0.01034 |
| CHR5_75615045_A_G | 0.000725 |
| CHR11_79825735_C_T | 0.001945 |
| CHR1_107547807_C_T | 0.003398 |
| CHR11_79825974_G_T | 0.001796 |
| CHR11_7487058_C_T | 0.008907 |
| CHR5_75461731_G_A | 0.000606 |
| CHR2_230042646_A_C | 0.004208 |
| CHR5_75530910_G_A | 0.000563 |
| CHR21_30829780_G_A | 0.000491 |
| CHR1_157000648_G_A | 0.001847 |
| CHR5_75581978_T_C | 0.000621 |
| age | 0.120236 |
| bmi | 0.123867 |
| pc0 | 0.045459 |
| pc1 | 0.048333 |
| pc2 | 0.044837 |
| pc3 | 0.044773 |
| pc4 | 0.042796 |
| pc5 | 0.046687 |
| pc6 | 0.045472 |
| pc7 | 0.046865 |
| pc8 | 0.050072 |
| pc9 | 0.04769 |

**Supplementary table S13.** Feature importance in the polygenic score model for HDL-C levels in the general population sample

| **Feature** | **Importance** |
| --- | --- |
| CHR16_56956804_C_A | 0.005662 |
| CHR16_56957451_C_T | 0.007542 |
| CHR16_56955678_C_T | 0.004862 |
| CHR16_56960332_T_TA | 0.002977 |
| CHR16_56959412_C_A | 0.005424 |
| CHR16_56960616_C_T | 0.003476 |
| CHR16_56953103_C_T | 0.002856 |
| CHR16_56953457_T_C | 0.003052 |
| CHR16_56959249_G_A | 0.003724 |
| CHR16_56953853_C_T | 0.003385 |
| CHR16_56967362_AC_A | 0.001481 |
| CHR16_56954132_C_T | 0.001142 |
| CHR16_56971389_C_T | 0.001271 |
| CHR16_56971567_C_A | 0.000935 |
| CHR16_56962376_G_A | 0.001316 |
| CHR16_56962299_G_A | 0.000924 |
| CHR16_56962733_GCC_G | 0.001277 |
| CHR16_56970977_G_A | 0.000809 |
| CHR16_56962737_C_A | 0.001226 |
| CHR16_56967526_G_A | 0.000918 |
| CHR16_56967304_C_T | 0.000654 |
| CHR16_56965416_C_T | 0.000791 |
| CHR16_56962246_T_C | 0.00124 |
| CHR16_56961324_C_A | 0.001429 |
| CHR16_56965006_A_G | 0.000975 |
| CHR16_56968820_T_G | 0.00347 |
| CHR16_56951602_G_A | 0.001452 |
| CHR16_56967342_T_TCACA | 0.002199 |
| CHR16_56966973_G_A | 0.002831 |
| CHR16_56965866_CG_C | 0.001858 |
| CHR16_56966784_T_A | 0.001615 |
| CHR16_56972466_A_G | 0.001013 |
| CHR16_56972678_C_T | 0.001641 |
| CHR16_56965346_A_C | 0.000877 |
| CHR16_56972917_T_A | 0.001008 |
| CHR16_56963437_C_CA | 0.002801 |
| CHR16_56963321_G_A | 0.002998 |
| CHR16_56959997_A_C | 0.001595 |
| CHR16_56968751_C_G | 0.001212 |
| CHR16_56955918_G_A | 0.001133 |
| CHR16_56973534_T_G | 0.001586 |
| CHR16_56966768_CT_C | 0.000788 |
| CHR16_56952850_G_T | 0.001399 |
| CHR16_56967026_G_A | 0.000548 |
| CHR16_56964660_G_C | 0.001133 |
| CHR16_56960982_G_A | 0.001041 |
| CHR16_56957712_G_A | 0.000824 |
| CHR16_56957829_C_T | 0.000779 |
| CHR16_56959299_T_C | 0.000838 |
| CHR16_56951227_A_G | 0.001824 |
| CHR16_56969234_T_C | 0.000786 |
| CHR15_58563549_C_T | 0.00246 |
| CHR16_56951244_A_G | 0.002043 |
| CHR16_56951643_A_G | 0.001517 |
| CHR15_58387985_C_T | 0.001157 |
| CHR15_58387469_G_A | 0.001338 |
| CHR15_58386521_T_C | 0.00132 |
| CHR15_58387608_CAGA_C | 0.001411 |
| CHR16_56960300_T_C | 0.001102 |
| CHR15_58567196_G_A | 0.003174 |
| CHR16_56959113_G_T | 0.001042 |
| CHR15_58387979_T_C | 0.001178 |
| CHR15_58388444_G_T | 0.001065 |
| CHR15_58399949_G_T | 0.001353 |
| CHR15_58382109_G_A | 0.001394 |
| CHR16_56980963_A_G | 0.001643 |
| CHR15_58386313_C_T | 0.001384 |
| CHR8_20002028_G_A | 0.001084 |
| CHR8_19967156_C_T | 0.001002 |
| CHR15_58388440_T_C | 0.001238 |
| CHR15_58388439_T_C | 0.001608 |
| CHR18_71032991_C_T | 0.000525 |
| CHR15_58381250_GA_G | 0.001079 |
| CHR15_58391343_G_C | 0.001065 |
| CHR8_19966981_T_C | 0.001193 |
| CHR16_56977273_G_A | 0.001419 |
| CHR18_49656294_A_G | 0.002907 |
| CHR16_56976320_C_T | 0.001038 |
| CHR18_71062516_G_A | 0.000799 |
| CHR15_58400516_C_T | 0.000987 |
| CHR16_56975476_G_A | 0.001412 |
| CHR15_58391858_A_G | 0.001011 |
| CHR8_19966137_A_T | 0.000895 |
| CHR18_71056827_C_T | 0.000902 |
| CHR16_56977540_G_A | 0.001333 |
| CHR8_19961928_A_G | 0.000919 |
| CHR16_56978787_A_G | 0.001363 |
| CHR18_49682332_A_G | 0.001768 |
| CHR15_58396988_T_C | 0.00092 |
| CHR16_56976574_C_T | 0.000349 |
| CHR15_58406811_C_T | 0.000583 |
| CHR15_58388755_T_C | 0.001392 |
| CHR18_49681721_A_G | 0.001533 |
| CHR15_58410665_T_C | 0.001562 |
| CHR18_71099062_T_C | 0.001185 |
| CHR18_49685882_A_G | 0.001549 |
| CHR15_58383833_A_T | 0.001149 |
| CHR8_20009934_C_G | 0.000827 |
| CHR15_58390297_G_C | 0.000735 |
| CHR15_58391167_A_G | 0.001258 |
| CHR15_58384622_G_A | 0.001253 |
| CHR15_58405635_C_G | 0.000783 |
| CHR15_58409732_A_G | 0.000756 |
| CHR22_47938294_G_GA | 0.000956 |
| CHR15_58400737_C_T | 0.001037 |
| CHR15_58395053_GA_G | 0.000588 |
| CHR8_20009120_A_G | 0.000968 |
| CHR15_58395331_G_A | 0.000668 |
| CHR15_58406158_G_A | 0.000651 |
| CHR15_58409352_A_G | 0.00076 |
| CHR2_56022155_G_GCGCA | 0.001501 |
| CHR16_56879601_C_T | 0.000819 |
| CHR16_56968492_C_A | 0.000755 |
| CHR15_58396998_C_T | 0.000686 |
| CHR1_40377660_G_A | 0.000429 |
| CHR15_58382496_G_C | 0.001081 |
| CHR11_120766238_C_T | 0.001743 |
| CHR18_49669395_G_A | 0.001436 |
| CHR8_20012752_T_A | 0.000268 |
| CHR8_20005513_C_T | 0.000976 |
| CHR16_56911137_C_A | 0.001333 |
| CHR16_56981179_G_C | 0.00044 |
| CHR18_49633082_A_AT | 0.001339 |
| CHR16_56968073_A_C | 0.000814 |
| CHR8_20012476_A_G | 0.000133 |
| CHR8_20005277_A_T | 0.000804 |
| CHR8_20009709_T_C | 0.000175 |
| CHR15_58410901_T_C | 0.000871 |
| CHR8_20012165_G_A | 8.18E-05 |
| CHR15_58395404_T_C | 0.00213 |
| CHR8_20011377_T_C | 6.11E-05 |
| CHR8_20010342_G_A | 4.17E-05 |
| CHR15_58411865_G_T | 0.000486 |
| CHR8_20009367_A_T | 8.34E-05 |
| CHR8_20010875_A_C | 9.34E-05 |
| CHR16_386726_C_CA | 0.000286 |
| CHR8_20009083_C_T | 4.46E-05 |
| CHR8_20011428_G_A | 8.54E-05 |
| CHR8_20011553_G_A | 0.000162 |
| CHR8_20009889_C_T | 0.000144 |
| CHR16_56904104_A_G | 0.000609 |
| CHR16_56887917_C_T | 0.000512 |
| CHR16_56866188_A_G | 0.001802 |
| CHR8_20010025_G_T | 8.72E-05 |
| CHR15_58381852_G_A | 0.00093 |
| CHR8_20009983_G_T | 0.000196 |
| CHR8_20000988_T_A | 6.33E-05 |
| CHR8_20012533_G_C | 0.000159 |
| CHR4_48727648_T_A | 0.000381 |
| CHR8_20003715_T_C | 0.000685 |
| CHR8_20010591_C_T | 7.3E-05 |
| CHR8_20011855_G_T | 9.59E-05 |
| CHR6_43244755_G_A | 0.002347 |
| CHR16_56966037_A_G | 0.000858 |
| CHR16_56865094_C_T | 0.001761 |
| CHR8_20000832_C_T | 6.29E-05 |
| CHR8_20007664_A_G | 0.000103 |
| CHR16_56896638_G_A | 0.00032 |
| CHR8_20006965_CA_C | 8.39E-05 |
| CHR8_20008763_G_A | 0.000105 |
| CHR8_20012516_G_C | 0.000172 |
| CHR8_20007238_C_T | 9.85E-05 |
| CHR8_20010866_G_C | 7.76E-05 |
| CHR8_20012504_T_G | 0.000164 |
| CHR8_19970337_C_A | 0.000833 |
| CHR8_20007752_T_G | 5.42E-05 |
| CHR8_20002116_C_T | 7.84E-05 |
| CHR8_20005059_T_C | 5.23E-05 |
| CHR15_58379566_G_T | 0.001867 |
| CHR12_65957337_G_A | 0.000127 |
| CHR8_20010779_T_C | 3.86E-05 |
| CHR16_23796059_G_A | 0.000733 |
| CHR8_20007176_G_A | 8.19E-05 |
| CHR8_20003850_A_C | 8.93E-05 |
| CHR8_20005996_C_T | 0.000105 |
| CHR17_21318324_C_T | 0 |
| CHR8_20007237_G_C | 5.57E-05 |
| CHR18_49683385_G_A | 0.001577 |
| CHR8_20007998_C_A | 9.73E-05 |
| CHR12_65948093_C_T | 0.000274 |
| CHR8_20012576_G_A | 0.000232 |
| CHR18_49640844_T_C | 0.001592 |
| CHR16_56895645_T_A | 0.000181 |
| CHR16_56897008_T_C | 0.00019 |
| CHR8_20007045_T_C | 7.31E-05 |
| CHR8_19998333_GTATTTT_G | 6.71E-05 |
| CHR16_56972393_C_T | 0.000812 |
| CHR16_56897136_G_C | 0.000246 |
| CHR8_19998089_T_C | 4.04E-05 |
| CHR8_20003236_G_A | 6.64E-05 |
| CHR4_70595190_CT_C | 0.000895 |
| CHR8_20008236_C_T | 0.000189 |
| CHR4_48785549_G_C | 0.000344 |
| CHR16_56895252_C_T | 0.000247 |
| CHR16_56895929_T_C | 0.000253 |
| CHR16_56897409_A_G | 0.000251 |
| CHR16_56895644_T_C | 0.000186 |
| CHR18_49661607_G_A | 0.001268 |
| CHR8_19962894_G_A | 0.000296 |
| CHR5_71611793_C_CT | 0.00141 |
| CHR12_65949088_C_T | 7.67E-05 |
| CHR12_65946506_T_A | 0.0006 |
| CHR16_56895597_A_T | 0.000213 |
| CHR16_56897653_C_T | 0.000305 |
| CHR12_65939995_T_G | 0.000126 |
| CHR16_56897553_T_A | 0.000257 |
| CHR8_20004709_C_T | 4.68E-05 |
| CHR8_19967357_G_A | 0.000318 |
| CHR16_56864286_A_G | 0.001738 |
| CHR18_49667573_G_C | 0.001223 |
| CHR8_19994799_C_G | 9.03E-05 |
| CHR16_88142954_CAG_C | 0.00035 |
| CHR15_58408112_T_C | 0.000772 |
| CHR20_53851945_G_A | 0.002136 |
| CHR16_56894763_C_A | 0.000115 |
| CHR16_56895768_G_T | 0.000193 |
| CHR16_56894304_C_T | 8.95E-05 |
| CHR16_56894148_T_C | 0.000128 |
| CHR16_56896753_T_A | 0.000136 |
| CHR16_56972387_GC_G | 0.000659 |
| CHR16_56898619_G_A | 0.000311 |
| CHR8_19995428_A_T | 0.000154 |
| CHR8_19995318_G_T | 0.000124 |
| CHR16_56902360_T_C | 0.000171 |
| CHR16_56896031_C_T | 0.000129 |
| CHR16_56896723_G_A | 0.000104 |
| CHR16_56894165_A_G | 0.000173 |
| CHR8_19996756_C_T | 0.0001 |
| CHR16_56894038_C_T | 0.000148 |
| CHR12_65961168_C_T | 0.000301 |
| CHR8_19994534_T_A | 0.000118 |
| CHR15_58406556_T_G | 0.001372 |
| CHR16_56899308_A_G | 0.000242 |
| CHR18_49630556_C_A | 0.002431 |
| CHR8_19994851_T_C | 0.000165 |
| CHR16_56896007_C_T | 0.000173 |
| CHR16_56903168_G_A | 0.00014 |
| CHR8_19996850_G_T | 9.84E-05 |
| CHR8_19997820_T_A | 8.31E-05 |
| CHR8_19998150_A_G | 8.82E-05 |
| CHR16_56898410_T_C | 0.00033 |
| CHR8_19997833_G_A | 7.09E-05 |
| CHR8_19997378_A_G | 7.99E-05 |
| CHR20_22560078_C_T | 0.000766 |
| CHR16_56896339_T_C | 0.000125 |
| CHR16_56902746_A_G | 0.000444 |
| CHR16_56895989_A_G | 0.000104 |
| CHR16_56902651_A_G | 0.000163 |
| CHR16_56904610_C_T | 0.000318 |
| CHR16_56894270_G_A | 0.000105 |
| CHR8_19997126_C_A | 5.9E-05 |
| CHR8_19995007_C_T | 0.000153 |
| CHR16_56894793_A_C | 0.000166 |
| CHR8_19996989_C_T | 4.82E-05 |
| CHR8_19996998_C_T | 5.95E-05 |
| CHR15_58417551_G_A | 0.000839 |
| CHR8_19995989_G_A | 6.14E-05 |
| CHR16_56896317_G_T | 8.99E-05 |
| CHR16_56893808_T_C | 0.000149 |
| CHR8_19997149_C_T | 8.75E-05 |
| CHR16_56896519_A_G | 0.000135 |
| CHR21_46303757_T_TA | 0.006883 |
| CHR15_58420618_C_G | 0.000753 |
| CHR8_19996076_G_A | 9.86E-05 |
| CHR15_58416982_C_T | 0.000733 |
| CHR8_19995075_T_C | 0.000212 |
| CHR16_56894368_G_C | 0.000118 |
| CHR16_56895532_T_A | 0.000142 |
| CHR8_19997171_T_C | 5.95E-05 |
| CHR8_19997710_C_T | 7.73E-05 |
| CHR8_20012760_C_A | 0.000801 |
| CHR16_56896008_T_G | 0.000127 |
| CHR8_19997761_T_C | 7.08E-05 |
| CHR8_19995878_A_C | 9.41E-05 |
| CHR8_19997042_C_A | 3.36E-05 |
| CHR16_56903294_G_A | 0.000188 |
| CHR16_56893416_A_C | 8.28E-05 |
| CHR16_56893935_A_G | 8.77E-05 |
| CHR16_56902766_GTCC_G | 0.000471 |
| CHR4_81644054_G_C | 0.003682 |
| CHR15_58407738_C_T | 0.00208 |
| CHR16_56893529_G_C | 0.000162 |
| CHR18_49657424_A_G | 0.001208 |
| CHR16_56903054_C_G | 0.000175 |
| CHR16_56893846_C_A | 0.000148 |
| CHR16_56906601_T_C | 0.000181 |
| CHR15_58431740_G_A | 0.002523 |
| CHR16_56952133_G_T | 0.000876 |
| CHR2_232430709_A_AT | 0.001753 |
| CHR16_56895017_GA_G | 0.000313 |
| CHR16_56958105_G_A | 0.000934 |
| CHR16_56958930_G_T | 0.000781 |
| CHR14_67233111_T_TTTTTA | 0.001031 |
| CHR16_56895108_G_C | 0.000175 |
| CHR16_23800523_A_C | 0.001093 |
| CHR16_56901054_CCT_C | 0.000168 |
| CHR16_56893384_C_T | 0.000216 |
| CHR6_43232144_A_G | 0.001732 |
| CHR5_87412693_G_T | 0.001478 |
| CHR15_58400003_A_C | 0.002041 |
| CHR4_169898254_T_G | 0.000596 |
| CHR17_4552908_A_C | 0.006286 |
| CHR15_58431227_A_G | 0.002968 |
| CHR16_56897792_G_A | 0.000295 |
| CHR8_19962025_T_G | 0.000455 |
| CHR16_56904868_C_G | 0.000163 |
| CHR16_23748049_G_A | 0.000613 |
| CHR15_94156195_T_G | 0.000225 |
| CHR16_56894964_A_C | 0.000174 |
| CHR15_58431280_T_C | 0.002686 |
| CHR16_56901178_G_C | 0.000301 |
| CHR12_65958792_C_T | 0.000217 |
| CHR16_56903417_C_T | 0.000152 |
| CHR18_49657982_T_G | 0.002704 |
| CHR18_49657636_G_A | 0.0014 |
| CHR16_23808649_A_G | 0.000853 |
| CHR8_19961706_A_C | 0.000459 |
| CHR12_65958038_T_C | 0.000135 |
| CHR8_19980958_C_T | 0.001688 |
| CHR13_110638900_G_A | 0.001823 |
| CHR6_27164172_T_A | 0.000512 |
| CHR22_29647045_A_AAG | 0.002192 |
| CHR16_56888856_C_A | 0.000241 |
| CHR12_51019946_A_T | 0.00036 |
| CHR19_16434935_CT_C | 0.002696 |
| CHR15_58434545_G_C | 0.002773 |
| CHR8_19961566_T_G | 0.000309 |
| CHR16_56886192_G_A | 0.000391 |
| CHR16_56888748_C_T | 0.00029 |
| CHR16_56963457_A_T | 0.000919 |
| CHR5_29870475_C_G | 0.001768 |
| CHR16_56907226_C_T | 0.000352 |
| CHR15_40724889_A_AT | 0.000787 |
| CHR11_78461783_C_CA | 0.000449 |
| CHR6_101793639_G_T | 0.00208 |
| CHR18_49658314_G_A | 0.001142 |
| CHR6_27155494_T_A | 0.000594 |
| CHR6_43231440_T_C | 0.002054 |
| CHR15_58431476_C_T | 0.002305 |
| CHR12_49959342_C_T | 0.000736 |
| CHR7_30162494_G_GT | 0.000538 |
| CHR12_65955010_A_G | 0.000197 |
| CHR1_69764790_CAGAT_C | 0.002763 |
| CHR15_58438299_C_T | 0.002229 |
| CHR16_56960280_C_T | 0.00103 |
| CHR11_116965760_T_A | 0.003608 |
| CHR19_16349246_C_CAA | 0.005687 |
| CHR16_56963639_C_T | 0.000856 |
| CHR16_56985480_T_C | 0.002775 |
| CHR16_56960023_C_T | 0.000869 |
| CHR18_12624542_CA_C | 0.000501 |
| CHR12_50046015_T_C | 0.000243 |
| CHR19_56164639_CA_C | 0.001051 |
| CHR12_50046019_T_C | 0.000222 |
| CHR18_49717542_A_C | 0.002759 |
| CHR12_113505545_T_C | 0.000767 |
| CHR18_49631864_C_T | 0.001668 |
| CHR16_56909009_A_T | 0.001044 |
| CHR2_3298142_T_C | 0.001129 |
| CHR5_87255443_T_C | 0.000753 |
| CHR4_52070614_A_G | 0.000433 |
| CHR15_58420004_T_G | 0.001022 |
| CHR3_85393304_G_A | 0.000704 |
| CHR16_56983407_G_A | 0.000265 |
| CHR10_16974429_CTTT_C | 0.000349 |
| CHR20_22478460_G_A | 0.000674 |
| CHR8_19958567_T_TA | 0.00051 |
| CHR8_20006328_G_GA | 0.000129 |
| CHR11_35165046_G_A | 0.000862 |
| CHR4_169946385_A_G | 0.000613 |
| CHR12_51233005_C_G | 0.000122 |
| CHR16_56883470_C_T | 0.00029 |
| CHR16_56882035_C_T | 0.000393 |
| CHR8_19959890_A_G | 0.000398 |
| CHR6_43221202_C_T | 0.002048 |
| CHR12_50044067_T_A | 0.000183 |
| CHR10_73139216_CA_C | 0.000652 |
| CHR8_20011460_T_C | 0.000552 |
| CHR16_10132340_CA_C | 0.00065 |
| CHR8_19936070_C_T | 0.000523 |
| CHR8_19958108_T_C | 0.000415 |
| CHR5_176556502_T_G | 0.001201 |
| CHR3_85202120_G_A | 0.000671 |
| CHR13_110638909_G_A | 0.001921 |
| CHR8_92869433_C_CA | 0.000576 |
| CHR17_68904281_C_CA | 0.003185 |
| CHR16_56897091_C_CA | 0.000446 |
| CHR8_19960531_G_A | 0.001429 |
| CHR8_19958860_T_C | 0.000483 |
| CHR4_51937984_G_A | 0.00035 |
| CHR3_184513987_G_C | 0.002935 |
| CHR8_19959423_T_C | 0.000462 |
| CHR3_85449083_A_ATT | 0.00126 |
| CHR8_20085797_A_G | 0.002267 |
| CHR3_33992700_A_G | 0.000914 |
| CHR3_184512980_A_G | 0.003202 |
| CHR3_85124678_T_G | 0.001221 |
| CHR16_34148044_A_T | 0.000308 |
| CHR6_43150069_TGATAA_T | 0.000937 |
| CHR19_53193973_A_G | 0.003144 |
| CHR13_110638917_T_C | 0.001761 |
| CHR8_20084670_G_T | 0.002807 |
| CHR8_20082955_A_AT | 0.00186 |
| CHR3_85586502_C_T | 0.004684 |
| CHR8_19959850_T_G | 0.000505 |
| CHR18_49700964_A_G | 0.002536 |
| CHR4_52478296_G_A | 0.002426 |
| CHR8_19958341_T_C | 0.000387 |
| CHR20_22541187_G_T | 0.000584 |
| CHR8_19958727_A_C | 0.000459 |
| CHR2_232439835_T_C | 0.000559 |
| CHR16_56896032_A_G | 0.002916 |
| CHR11_35182575_A_G | 0.000725 |
| CHR3_85567967_C_A | 0.005161 |
| CHR3_12499474_A_G | 0.000778 |
| CHR8_19994665_TTC_T | 0.000762 |
| CHR2_3303013_A_C | 0.001098 |
| CHR2_3299603_T_TTC | 0.001167 |
| CHR4_51876384_A_G | 0.000896 |
| CHR19_53194115_T_G | 0.003169 |
| CHR20_22657100_T_C | 0.000515 |
| CHR4_169974243_G_A | 0.000421 |
| CHR3_85092645_G_A | 0.000827 |
| CHR3_54530748_G_T | 0.001174 |
| CHR3_184505666_A_G | 0.002578 |
| CHR20_7870460_A_AT | 0.000447 |
| CHR4_67062220_T_C | 0.001025 |
| CHR15_99872179_A_G | 0.001395 |
| CHR7_53391451_T_C | 0.000754 |
| CHR15_58379522_G_A | 0.001549 |
| CHR2_232435235_C_G | 0.000417 |
| CHR2_232433924_T_C | 0.000501 |
| CHR6_27386164_C_T | 0.000417 |
| CHR2_232434958_A_G | 0.000462 |
| CHR13_110638919_T_C | 0.001646 |
| CHR8_128026848_C_T | 0.001623 |
| CHR2_78110238_T_C | 0.000221 |
| CHR20_7877279_G_A | 0.000576 |
| CHR16_26574444_C_T | 0.001169 |
| CHR2_232434354_C_T | 0.000514 |
| CHR10_26100645_C_T | 0.001453 |
| CHR5_87445049_T_C | 0.000834 |
| CHR2_232430353_A_T | 0.000616 |
| CHR3_12609052_T_C | 0.001112 |
| CHR2_232436038_A_G | 0.000427 |
| CHR4_48982716_G_A | 0.000416 |
| CHR2_232435003_G_A | 0.000452 |
| CHR11_97017916_G_A | 0.00085 |
| CHR5_87326450_A_G | 0.000957 |
| CHR2_232435336_T_C | 0.000378 |
| CHR8_20082711_C_CTT | 0.001821 |
| CHR2_232430354_G_A | 0.00065 |
| CHR2_232432278_G_C | 0.000424 |
| CHR8_20084343_A_G | 0.001746 |
| CHR5_179626956_T_C | 0.00148 |
| CHR2_232413330_T_C | 0.00077 |
| CHR16_56957612_G_A | 0.000936 |
| CHR2_232435570_A_G | 0.000472 |
| CHR16_57067088_T_G | 0.000253 |
| CHR18_49631816_T_C | 0.001573 |
| CHR2_232429063_A_G | 0.000433 |
| CHR16_57065531_G_A | 0.000262 |
| CHR2_232434834_T_A | 0.00039 |
| CHR6_27348873_C_T | 0.000408 |
| CHR6_160587614_G_A | 0.002145 |
| CHR5_87310837_G_A | 0.001224 |
| CHR16_56954590_G_A | 0.001186 |
| CHR3_184513982_G_C | 0.002604 |
| CHR1_7721974_G_A | 0.000387 |
| Sex_from_meta_manual | 0.189785 |
| age | 0.015936 |
| bmi | 0.138752 |
| pc0 | 0.018106 |
| pc1 | 0.019106 |
| pc2 | 0.018584 |
| pc3 | 0.018905 |
| pc4 | 0.01859 |
| pc5 | 0.018433 |
| pc6 | 0.017111 |
| pc7 | 0.021383 |
| pc8 | 0.019374 |
| pc9 | 0.018796 |

**Supplementary table S14.** Feature importance in the polygenic score model for LDL-C levels in the general population sample

| **Predictor** | **Coefficient** |
| --- | --- |
| CHR19_44909976_G_T | 0.014823 |
| CHR19_44908822_C_T | 0.013441 |
| CHR19_44911142_C_A | 0.003405 |
| CHR19_44912383_G_A | 0.001972 |
| CHR19_44913574_T_G | 0.002129 |
| CHR19_44879418_G_A | 0.003273 |
| CHR19_44816374_G_A | 0.000707 |
| CHR19_44897490_T_A | 0.001032 |
| CHR19_44899005_T_G | 0.000863 |
| CHR19_44893972_G_A | 0.00089 |
| CHR19_44886339_G_A | 0.001016 |
| CHR19_44908684_T_C | 0.003835 |
| CHR19_44889415_AT_A | 0.000945 |
| CHR19_44912456_G_A | 0.001765 |
| CHR19_44912678_G_T | 0.001666 |
| CHR19_44799692_GAA_G | 0.00044 |
| CHR19_44923868_T_A | 0.001427 |
| CHR19_44924977_G_A | 0.001503 |
| CHR19_44916968_T_TA | 0.000611 |
| CHR19_44918903_C_G | 0.00232 |
| CHR19_44917997_G_A | 0.001466 |
| CHR19_44919589_G_A | 0.002255 |
| CHR19_44799247_G_A | 0.000277 |
| CHR19_44906745_G_A | 0.001572 |
| CHR19_44921095_A_T | 0.0015 |
| CHR19_44919689_A_G | 0.00218 |
| CHR19_44921257_A_C | 0.002067 |
| CHR19_44921094_A_T | 0.002037 |
| CHR19_44728895_G_A | 0.000661 |
| CHR19_44920730_C_CA | 0.001395 |
| CHR19_44748549_G_T | 0.000667 |
| CHR19_44740135_A_AAT | 0.000525 |
| CHR2_21065354_G_A | 0.001781 |
| CHR19_44943964_G_A | 0.000567 |
| CHR2_21063185_T_C | 0.002397 |
| CHR2_21066560_G_A | 0.00178 |
| CHR19_44883800_A_G | 0.000772 |
| CHR19_44883777_A_C | 0.000775 |
| CHR2_21065449_A_G | 0.00161 |
| CHR2_20951027_T_C | 0.003661 |
| CHR5_75348881_T_G | 0.000272 |
| CHR5_75329662_C_A | 0.000223 |
| CHR5_75348960_T_TA | 0.000158 |
| CHR5_75340659_C_A | 0.000374 |
| CHR5_75330257_T_C | 0.000215 |
| CHR5_75352778_A_T | 0.000166 |
| CHR5_75344665_C_T | 0.000133 |
| CHR5_75343751_C_CAA | 0.000151 |
| CHR5_75341886_A_T | 0.000186 |
| CHR5_75341527_G_GCTTA | 0.000164 |
| CHR5_75359901_C_T | 0.000298 |
| CHR5_75343434_T_G | 0.000159 |
| CHR5_75328657_T_C | 0.000162 |
| CHR5_75278499_A_C | 0.000881 |
| CHR5_75348173_A_ATTC | 0.000216 |
| CHR5_75332550_A_T | 0.000165 |
| CHR2_20993943_C_A | 0.002969 |
| CHR5_75347023_A_T | 0.000223 |
| CHR5_75356084_G_A | 0.000258 |
| CHR5_75321437_G_A | 0.000343 |
| CHR5_75325846_A_G | 0.000323 |
| CHR5_75343353_A_G | 0.000262 |
| CHR19_44888997_C_T | 0.001457 |
| CHR19_11076935_A_G | 0.000324 |
| CHR19_11076682_T_G | 0.000221 |
| CHR5_75279159_A_G | 0.000379 |
| CHR5_75329576_C_CT | 0.000746 |
| CHR19_11087074_G_GA | 0.000359 |
| CHR19_44892362_A_G | 0.000747 |
| CHR19_44892887_C_T | 0.000729 |
| CHR19_11076746_T_C | 0.000243 |
| CHR5_75291041_CT_C | 0.00035 |
| CHR5_75288207_T_C | 0.000343 |
| CHR19_44892652_C_G | 0.000724 |
| CHR19_11076648_C_G | 0.000186 |
| CHR19_44891079_T_C | 0.000988 |
| CHR5_75280460_G_A | 0.000441 |
| CHR5_75339400_G_C | 0.000369 |
| CHR19_11086922_G_T | 0.000142 |
| CHR19_11078690_C_CAG | 8.26E-05 |
| CHR19_11078174_T_C | 0.00012 |
| CHR19_11079398_G_A | 8E-05 |
| CHR19_11077637_C_CTTTA | 8.18E-05 |
| CHR19_11078529_C_G | 0.000115 |
| CHR19_11079261_T_A | 7.65E-05 |
| CHR2_21016795_T_C | 0.002332 |
| CHR19_11079616_T_C | 7.92E-05 |
| CHR19_11082415_T_G | 8.19E-05 |
| CHR19_11086585_G_A | 0.000129 |
| CHR19_11081053_C_T | 6.69E-05 |
| CHR19_11078298_AC_A | 0.000101 |
| CHR5_75306874_C_A | 0.000441 |
| CHR5_75360714_T_C | 0.001769 |
| CHR19_11079805_G_T | 6E-05 |
| CHR19_11079434_A_G | 5.83E-05 |
| CHR19_11079304_C_A | 6.99E-05 |
| CHR5_75274031_G_T | 0.00042 |
| CHR5_75315749_G_T | 0.000226 |
| CHR19_11077477_A_C | 8.11E-05 |
| CHR19_11085680_AC_A | 0.000125 |
| CHR5_75326296_TTA_T | 0.000322 |
| CHR19_11080521_G_A | 6.58E-05 |
| CHR19_11079858_G_A | 7.86E-05 |
| CHR19_11079088_T_C | 8.84E-05 |
| CHR19_11079868_C_T | 0.000106 |
| CHR5_75272410_G_A | 0.00038 |
| CHR19_11078223_C_T | 8.95E-05 |
| CHR19_11078596_T_C | 6.73E-05 |
| CHR19_11077441_C_T | 7.38E-05 |
| CHR19_11078622_CG_C | 6.37E-05 |
| CHR19_11077488_T_C | 8.26E-05 |
| CHR19_11090448_T_C | 0.000115 |
| CHR19_11080197_C_T | 0.000101 |
| CHR5_75266739_T_C | 0.00103 |
| CHR19_11079880_T_C | 7.43E-05 |
| CHR19_11079976_G_A | 7.44E-05 |
| CHR5_75317068_G_A | 0.000269 |
| CHR19_11079873_G_A | 0.000104 |
| CHR19_11078371_TA_T | 0.000339 |
| CHR5_75355259_A_G | 0.001251 |
| CHR19_44739710_C_T | 0.000669 |
| CHR19_44827846_A_G | 0.002377 |
| CHR19_11077571_G_A | 9.34E-05 |
| CHR5_75312907_C_T | 0.000223 |
| CHR19_11091630_G_T | 0.000217 |
| CHR5_75307654_G_A | 0.000227 |
| CHR19_44738916_G_A | 0.000642 |
| CHR19_11087826_T_C | 0.000151 |
| CHR19_11091518_GC_G | 0.000256 |
| CHR19_44744370_A_G | 0.000623 |
| CHR19_44884873_G_A | 0.000974 |
| CHR19_45041227_C_CA | 0.00145 |
| CHR19_44884339_G_A | 0.000676 |
| CHR19_44883210_G_GTAA | 0.000689 |
| CHR19_11091312_CHRG | 0.000265 |
| CHR19_44884202_C_G | 0.000671 |
| CHR5_75343719_CTTGTA_C | 0.000859 |
| CHR19_44752422_G_C | 0.00054 |
| CHR5_75308130_C_T | 0.000422 |
| CHR5_75325749_A_AT | 0.001061 |
| CHR19_11077868_CT_C | 0.000776 |
| CHR2_21050024_G_A | 0.001302 |
| CHR2_21051295_A_T | 0.00144 |
| CHR19_44752009_T_C | 0.000608 |
| CHR5_75309395_G_A | 0.00068 |
| CHR5_75319196_C_T | 0.000573 |
| CHR5_75321018_T_C | 0.000537 |
| CHR5_75584065_G_A | 0.000394 |
| CHR19_11082200_C_CT | 0.000259 |
| CHR5_75322361_G_A | 0.000547 |
| CHR19_44865946_G_A | 0.000522 |
| CHR5_75477595_T_C | 0.000127 |
| CHR5_75461731_G_A | 0.000149 |
| CHR5_75491485_T_C | 0.000148 |
| CHR5_75452680_C_T | 0.000145 |
| CHR5_75521119_C_G | 0.000163 |
| CHR5_75472522_C_A | 0.000163 |
| CHR5_75370667_C_T | 0.000172 |
| CHR2_21248731_T_C | 0.002546 |
| CHR19_11082239_A_G | 0.000155 |
| CHR5_75322120_G_A | 0.000652 |
| CHR19_11080525_A_AG | 0.000125 |
| CHR5_75463358_T_A | 0.000145 |
| CHR19_44750234_C_T | 0.001862 |
| CHR5_75513055_C_T | 0.000183 |
| CHR5_75220642_CA_C | 0.001012 |
| CHR5_75440775_TA_T | 0.000391 |
| CHR19_11086210_T_C | 0.00017 |
| CHR5_75393424_T_C | 0.000131 |
| CHR5_75581978_T_C | 0.000202 |
| CHR19_11082155_A_G | 0.000122 |
| CHR5_75232565_GTT_G | 0.000516 |
| CHR19_11081874_G_A | 0.000125 |
| CHR19_11081517_A_T | 0.000136 |
| CHR19_11079781_GAAAA_G | 0.000296 |
| CHR19_45150625_TAGTC_T | 0.000933 |
| CHR19_11080624_G_A | 0.000143 |
| CHR2_21196778_G_A | 0.001585 |
| CHR19_11081001_T_A | 0.000137 |
| CHR2_21199531_G_A | 0.000188 |
| CHR19_11083273_A_G | 0.000128 |
| CHR5_75530910_G_A | 0.000118 |
| CHR19_44750354_ATTGGC_A | 0.001773 |
| CHR5_75614147_C_T | 0.000359 |
| CHR19_11087511_C_T | 0.000195 |
| CHR5_75615045_A_G | 0.000136 |
| CHR2_21196937_A_T | 0.00017 |
| CHR5_75323814_C_T | 0.000565 |
| CHR2_21194704_C_T | 0.000232 |
| CHR2_21198910_C_A | 0.000171 |
| CHR2_21197825_A_G | 0.000181 |
| CHR2_21195884_A_G | 0.000189 |
| CHR2_21197486_C_A | 0.000164 |
| CHR2_21196448_C_G | 0.000194 |
| CHR2_21195847_C_A | 0.000172 |
| CHR19_44862466_TA_T | 0.001875 |
| CHR2_21203871_C_A | 0.000195 |
| CHR5_75512412_G_T | 0.000148 |
| CHR2_21201117_T_C | 0.000221 |
| CHR19_44905371_T_C | 0.001321 |
| CHR19_45028360_CHRG | 0.000582 |
| CHR2_21202451_A_G | 0.000166 |
| CHR5_75117888_T_C | 0.000465 |
| CHR5_75292432_G_A | 0.001147 |
| CHR2_21195211_G_A | 0.000189 |
| CHR2_21200056_C_T | 0.000321 |
| CHR2_21041028_G_A | 0.001717 |
| CHR19_45038524_G_A | 0.000631 |
| CHR2_21086250_C_T | 0.000455 |
| CHR19_44930070_T_TA | 0.000702 |
| CHR5_75065315_T_C | 0.000644 |
| CHR2_21086984_G_A | 0.000409 |
| CHR19_44881148_T_C | 0.002455 |
| CHR2_21092201_AT_A | 0.000458 |
| CHR2_21080598_T_C | 0.000508 |
| CHR5_75099216_T_C | 0.000482 |
| CHR19_44537330_C_T | 0.001057 |
| CHR19_44728555_G_A | 0.000595 |
| CHR2_20967152_C_T | 0.001734 |
| CHR5_75611138_A_C | 0.000772 |
| CHR2_21155687_CHRG | 0.000455 |
| CHR19_44892587_G_A | 0.001406 |
| CHR5_75104691_G_C | 0.000601 |
| CHR19_44929891_AAGGG_A | 0.000692 |
| CHR5_75076316_T_C | 0.000578 |
| CHR19_44736280_C_A | 0.000475 |
| CHR19_44732598_TGAA_T | 0.000494 |
| CHR19_11085975_AT_A | 0.00081 |
| CHR5_75097057_C_T | 0.000591 |
| CHR19_44933400_G_A | 0.000711 |
| CHR19_44732593_AAG_A | 0.000535 |
| CHR5_75087344_T_G | 0.000629 |
| CHR5_75080833_G_C | 0.000618 |
| CHR5_75524614_G_GA | 0.000307 |
| CHR5_75112166_C_T | 0.000529 |
| CHR5_75085132_A_G | 0.000716 |
| CHR2_21196630_T_TAC | 0.000642 |
| CHR2_21044589_G_A | 0.001776 |
| CHR5_75279976_A_G | 0.00116 |
| CHR2_21194559_C_A | 0.000449 |
| CHR19_44932209_C_CT | 0.000669 |
| CHR2_20998527_T_G | 0.001313 |
| CHR5_75292115_C_A | 0.00104 |
| CHR2_21009323_G_A | 0.00122 |
| CHR19_44928196_G_A | 0.00058 |
| CHR19_44743791_C_T | 0.001742 |
| CHR19_44730118_A_G | 0.000524 |
| CHR5_75085134_A_G | 0.000842 |
| CHR5_75276089_G_A | 0.000582 |
| CHR19_44746252_C_T | 0.000499 |
| CHR5_75299369_A_C | 0.001006 |
| CHR5_75067896_T_TA | 0.001631 |
| CHR2_21009932_G_A | 0.00127 |
| CHR2_21128454_C_T | 0.000326 |
| CHR19_11122020_C_T | 0.003526 |
| CHR2_21190866_G_A | 0.000329 |
| CHR19_44846145_T_C | 0.001735 |
| CHR19_44747899_A_C | 0.001864 |
| CHR2_21164085_T_C | 0.00027 |
| CHR1_109274968_G_T | 0.000993 |
| CHR5_75273532_C_T | 0.000614 |
| CHR5_75274706_G_A | 0.000578 |
| CHR5_75243979_T_TA | 0.00078 |
| CHR2_21162277_T_C | 0.000301 |
| CHR1_109274570_A_G | 0.00098 |
| CHR2_20946761_G_T | 0.001289 |
| CHR5_75293560_C_T | 0.001052 |
| CHR1_109278889_T_G | 0.001154 |
| CHR2_21158396_G_C | 0.000371 |
| CHR6_160678471_G_A | 0.000679 |
| CHR6_160678258_T_C | 0.000613 |
| CHR2_20946172_AC_A | 0.001222 |
| CHR19_44844654_C_A | 0.001619 |
| CHR5_75269328_C_T | 0.000628 |
| CHR2_21162289_C_G | 0.000286 |
| CHR2_20945069_G_T | 0.001109 |
| CHR19_44849230_A_T | 0.001872 |
| CHR2_20945739_G_A | 0.001225 |
| CHR19_44858389_A_G | 0.002887 |
| CHR2_21169417_C_G | 0.000351 |
| CHR6_160676839_A_G | 0.000713 |
| CHR5_75268365_A_G | 0.00058 |
| CHR12_130148997_G_A | 0.002743 |
| CHR2_21030749_CA_C | 0.001857 |
| CHR2_21158420_A_AAAAC | 0.00035 |
| CHR6_160683938_A_G | 0.000871 |
| CHR5_75128542_C_T | 3.54E-05 |
| CHR5_75323307_C_T | 0.001019 |
| CHR6_160678927_T_A | 0.000667 |
| CHR2_20944222_C_T | 0.001158 |
| CHR2_21248115_C_T | 0.000479 |
| CHR1_109275536_C_CT | 0.00119 |
| CHR6_160676789_C_T | 0.000572 |
| CHR19_44884984_T_G | 0.000909 |
| CHR2_21030756_A_T | 0.001879 |
| CHR2_21246464_C_T | 0.000475 |
| CHR2_20943917_C_A | 0.00119 |
| CHR6_160679753_A_G | 0.000589 |
| CHR2_21246866_G_T | 0.000469 |
| CHR2_21049891_AG_A | 0.000645 |
| CHR14_71540901_C_T | 0.001504 |
| CHR2_21246280_G_T | 0.000469 |
| CHR19_11083117_A_AT | 0.001174 |
| CHR19_11116804_A_C | 0.00234 |
| CHR6_160701242_A_G | 0.000463 |
| CHR13_106200380_T_G | 0.002043 |
| CHR6_31529014_C_G | 0.00146 |
| CHR6_160681799_C_T | 0.000499 |
| CHR2_21245000_ATT_A | 0.000496 |
| CHR18_11953858_C_T | 0.003141 |
| CHR5_75181894_T_TA | 4.58E-05 |
| CHR6_160685986_G_A | 0.000505 |
| CHR5_75186850_A_T | 7.6E-05 |
| CHR19_11097068_CTT_C | 0.001126 |
| CHR2_21163186_C_T | 0.000705 |
| CHR5_75174028_G_A | 0.000188 |
| CHR5_75140912_C_T | 0.000199 |
| CHR1_109275684_G_T | 0.000849 |
| CHR5_75582948_G_GA | 0.000809 |
| CHR2_21055901_G_T | 0.000369 |
| CHR5_75175223_C_G | 0.000217 |
| CHR5_75175865_G_A | 0.000242 |
| CHR5_75638184_A_T | 0.001322 |
| CHR5_75184174_G_A | 0.000161 |
| CHR6_150400294_G_A | 0.001935 |
| CHR19_44852884_C_T | 0.002467 |
| CHR5_75162395_C_T | 0.000214 |
| CHR5_75177103_C_T | 0.000228 |
| CHR1_109279544_G_A | 0.000975 |
| CHR6_160679463_A_C | 0.000623 |
| CHR5_75155913_CT_C | 0.00058 |
| CHR2_21048835_T_A | 0.001076 |
| CHR5_75093500_C_A | 0.001327 |
| CHR2_21089338_C_T | 0.000426 |
| CHR12_130153145_G_A | 0.002412 |
| CHR5_75272020_C_A | 0.000573 |
| CHR5_75615668_CTG_C | 0.001927 |
| CHR1_219199344_G_A | 0.00172 |
| CHR2_21079148_T_C | 0.000426 |
| CHR2_21238858_G_C | 0.000626 |
| CHR19_11116394_C_T | 0.002401 |
| CHR1_109275908_C_T | 0.000812 |
| CHR5_75076050_AAT_A | 5.66E-05 |
| CHR5_75237433_AT_A | 1.89E-05 |
| CHR5_75249832_C_CA | 3.55E-05 |
| CHR1_109279521_G_A | 0.000966 |
| CHR2_21059143_C_T | 0.000372 |
| CHR2_21111599_AT_A | 0.001515 |
| CHR5_75264662_C_T | 8.78E-05 |
| CHR5_75308917_G_A | 0.00143 |
| CHR19_44750285_C_CTTTG | 0.00176 |
| CHR2_21059706_C_G | 0.000409 |
| CHR2_21062839_C_A | 0.000343 |
| CHR5_75241926_C_T | 4.95E-05 |
| CHR5_75175910_A_G | 0.000208 |
| CHR5_75083850_T_A | 0.001193 |
| CHR2_21072355_G_A | 0.000888 |
| CHR1_109278685_G_T | 0.00093 |
| CHR6_160695384_C_T | 0.00046 |
| CHR6_160684121_A_G | 0.000617 |
| CHR2_21011100_T_C | 0.001516 |
| CHR5_75066397_A_G | 1.59E-05 |
| CHR2_21072567_A_T | 0.000852 |
| CHR2_21060030_G_A | 0.000358 |
| CHR6_160695433_C_T | 0.000435 |
| CHR5_75222492_T_TA | 5.45E-05 |
| CHR6_160696418_G_A | 0.000435 |
| CHR5_75180571_G_A | 8.12E-06 |
| CHR5_75072817_G_T | 1.88E-05 |
| CHR5_75067750_T_G | 1.94E-05 |
| CHR5_75181033_CAA_C | 0.000134 |
| CHR5_75255831_G_GC | 5.41E-05 |
| CHR1_109275216_T_C | 0.000935 |
| CHR2_230042646_A_C | 0.007477 |
| CHR5_75241781_A_G | 4.25E-05 |
| CHR2_21230861_A_G | 0.000534 |
| CHR5_75224450_T_A | 9.1E-06 |
| CHR19_44739483_A_G | 0.001925 |
| CHR14_71588361_C_G | 0.001364 |
| CHR5_75240830_G_A | 5.74E-05 |
| CHR5_75238547_A_G | 4.57E-05 |
| CHR5_75190607_ATG_A | 0.000615 |
| CHR5_75249723_A_T | 4.87E-05 |
| CHR10_49278682_G_A | 0.003055 |
| CHR5_75197153_C_T | 2.43E-05 |
| CHR5_75098823_A_G | 5.18E-06 |
| CHR5_75098838_T_C | 2.22E-05 |
| CHR19_44870308_G_A | 0.002041 |
| CHR5_75068475_G_A | 1.47E-05 |
| CHR5_75147307_C_T | 8.71E-06 |
| CHR5_75352671_G_T | 0.000433 |
| CHR5_75217162_A_G | 1.64E-05 |
| CHR5_75254892_A_AT | 5.35E-05 |
| CHR5_75244362_G_A | 5.8E-05 |
| CHR5_75069228_T_C | 8.01E-06 |
| CHR5_75202017_A_C | 1.39E-05 |
| CHR5_75065156_A_ATCT | 2.88E-05 |
| CHR2_20948514_A_C | 0.001471 |
| CHR5_75070161_C_T | 1.13E-05 |
| CHR5_75123968_G_A | 1.1E-05 |
| CHR5_75112169_T_C | 7.47E-06 |
| CHR5_75348856_G_T | 0.000448 |
| CHR5_75075378_T_G | 1.43E-05 |
| CHR19_58449563_C_T | 0.00421 |
| CHR5_75256003_A_G | 5.53E-05 |
| CHR19_44885917_T_A | 0.001408 |
| CHR6_160701184_T_C | 0.000451 |
| CHR5_75176404_G_C | 0.000237 |
| CHR5_75209670_A_C | 1.83E-05 |
| CHR5_75061032_T_C | 3.86E-05 |
| CHR5_75091492_C_G | 9.01E-06 |
| CHR5_75070198_A_G | 1.14E-05 |
| CHR5_75183018_A_C | 9.98E-06 |
| CHR5_75102316_A_G | 9.89E-06 |
| CHR5_75127514_A_G | 1.52E-05 |
| CHR5_75082917_C_T | 1.06E-05 |
| CHR2_21050618_G_T | 0.000634 |
| CHR6_160688021_A_G | 0.000514 |
| CHR5_75249973_A_G | 4.65E-05 |
| CHR5_75191550_A_C | 4.76E-05 |
| CHR14_71738229_T_G | 0.000957 |
| CHR5_75099143_A_G | 1.2E-05 |
| CHR5_75214528_G_T | 1.04E-05 |
| CHR5_75202159_T_C | 8.34E-06 |
| CHR2_21071167_A_C | 0.000318 |
| CHR5_75111406_T_A | 6.07E-06 |
| CHR5_75069766_C_G | 1.08E-05 |
| CHR5_75206972_G_A | 2.54E-05 |
| CHR5_75084101_C_T | 9.5E-06 |
| CHR2_155129145_C_G | 0.001364 |
| CHR5_75264754_G_A | 9.01E-05 |
| CHR5_75159238_A_G | 1.35E-05 |
| CHR5_75231309_A_G | 9.86E-06 |
| CHR5_75083800_A_T | 1.06E-05 |
| CHR5_75214692_T_G | 1.23E-05 |
| CHR5_75080484_A_C | 1.89E-05 |
| CHR5_75080746_C_T | 1.21E-05 |
| CHR5_75164220_A_G | 2.42E-05 |
| CHR5_75108032_T_A | 1.08E-05 |
| CHR5_75127418_A_T | 9.81E-06 |
| CHR5_75109924_A_T | 8.16E-06 |
| CHR5_75083600_T_C | 2.33E-05 |
| CHR5_75203594_T_C | 7.22E-06 |
| CHR5_75223224_G_A | 9.62E-06 |
| CHR5_75181024_A_C | 1.57E-05 |
| CHR5_75219003_T_C | 9.71E-06 |
| CHR5_75345265_CT_C | 0.00045 |
| CHR5_75137433_G_A | 7.73E-06 |
| CHR19_44869537_G_A | 0.002098 |
| CHR5_75202339_C_A | 5.11E-06 |
| CHR5_75097626_C_T | 7.98E-06 |
| CHR5_75069201_C_T | 1.02E-05 |
| CHR5_75256137_T_C | 5.06E-05 |
| CHR5_75126294_T_C | 1.38E-05 |
| CHR5_75083456_T_C | 2.21E-05 |
| CHR5_75180386_T_G | 9.88E-06 |
| CHR5_75147095_A_G | 5.34E-06 |
| CHR5_75174525_G_A | 1.07E-05 |
| CHR5_75122479_G_A | 9.83E-06 |
| CHR5_75170912_C_T | 6.01E-06 |
| CHR6_160698399_C_T | 0.00049 |
| CHR5_75161013_A_G | 6.19E-06 |
| CHR5_75157993_C_G | 8.54E-06 |
| CHR5_75110491_T_C | 6.7E-06 |
| CHR5_75186012_T_C | 4.93E-06 |
| CHR5_75250853_T_A | 6.64E-05 |
| CHR5_75259428_T_C | 0.000117 |
| CHR5_75207879_A_C | 1.17E-05 |
| CHR2_21055060_C_T | 0.000298 |
| CHR5_75109272_A_G | 1.66E-05 |
| CHR5_75237905_C_T | 4.66E-05 |
| CHR5_75141328_G_A | 5.8E-06 |
| CHR5_75077559_AT_A | 1.58E-05 |
| CHR5_75096701_C_A | 8.38E-06 |
| CHR5_75219633_GA_G | 8.45E-06 |
| CHR2_208097206_A_G | 0.003592 |
| CHR5_75145875_G_A | 1.16E-05 |
| CHR5_75146585_G_A | 1.02E-05 |
| CHR5_75135806_C_T | 4.27E-06 |
| CHR5_75233066_C_T | 1.94E-05 |
| CHR6_160699531_G_A | 0.000507 |
| CHR5_75201259_G_A | 1.41E-05 |
| CHR5_75190953_T_G | 6.61E-05 |
| CHR5_75229340_G_A | 1.64E-05 |
| CHR5_75243818_T_C | 5.67E-05 |
| CHR5_75163395_A_G | 1.24E-05 |
| CHR5_75158881_C_T | 1.14E-05 |
| CHR5_75141403_C_A | 7.01E-06 |
| CHR5_75166025_T_G | 1.36E-05 |
| CHR5_75196986_T_C | 8.39E-06 |
| CHR2_21058225_A_G | 0.000345 |
| CHR5_75101287_G_A | 9.42E-06 |
| CHR5_75214516_C_T | 1.39E-05 |
| CHR2_21200660_G_A | 0.000404 |
| CHR5_75257014_T_C | 4.99E-05 |
| CHR5_75161448_C_T | 1.27E-05 |
| CHR5_75210385_A_G | 1.65E-05 |
| CHR5_75173487_G_C | 1.27E-05 |
| CHR5_75263068_A_G | 5.9E-05 |
| CHR5_75100107_T_A | 8.59E-06 |
| CHR5_75105444_A_G | 1.08E-05 |
| CHR5_75238484_A_G | 4.16E-05 |
| CHR5_75167360_T_G | 1.77E-05 |
| CHR5_75183720_C_T | 4.93E-06 |
| CHR5_75206748_G_A | 9.42E-06 |
| CHR5_75149992_G_A | 4.18E-06 |
| CHR5_75194393_T_TG | 4.88E-06 |
| CHR5_75232951_G_A | 4.67E-06 |
| CHR5_75255610_T_C | 5.5E-05 |
| CHR5_75165850_C_T | 1.48E-05 |
| CHR5_75119263_T_C | 9.11E-06 |
| CHR5_75134309_A_G | 9.3E-06 |
| CHR5_75257792_C_T | 5.92E-05 |
| CHR6_31527842_A_T | 0.001477 |
| CHR5_75102824_T_G | 9.5E-06 |
| CHR5_75177114_G_A | 6.03E-06 |
| CHR5_75176400_T_C | 5.55E-06 |
| CHR5_75070708_C_T | 3.92E-05 |
| CHR5_75186358_C_T | 1.02E-05 |
| CHR5_75212196_T_A | 1.46E-05 |
| CHR5_75081176_T_C | 6.13E-06 |
| CHR5_75121180_T_TA | 0.00127 |
| CHR5_75171059_A_C | 8.1E-06 |
| CHR5_75168590_T_C | 5.86E-06 |
| CHR5_75173706_T_C | 5.92E-06 |
| CHR5_75177527_T_C | 1.17E-05 |
| CHR5_75155353_G_C | 2.52E-05 |
| CHR5_75168735_GA_G | 4.76E-06 |
| CHR5_75095841_C_T | 9.16E-06 |
| CHR5_75157202_T_G | 1.15E-05 |
| CHR5_75229012_A_G | 7.71E-06 |
| CHR2_21072960_G_A | 0.000864 |
| CHR8_142742301_G_T | 0.001981 |
| CHR5_75221805_AAT_A | 1.17E-05 |
| CHR5_75179993_C_T | 1.33E-05 |
| CHR5_75208327_G_A | 2.66E-06 |
| CHR5_75175201_G_A | 7.3E-06 |
| CHR5_75188450_C_T | 7.94E-06 |
| CHR5_75211182_C_A | 1.01E-05 |
| CHR5_75227730_C_T | 8.08E-06 |
| CHR5_75126137_A_G | 7.1E-06 |
| CHR5_75126385_T_C | 9.86E-06 |
| CHR5_75171100_C_G | 6.53E-06 |
| CHR5_75056355_A_G | 0.00144 |
| CHR5_75175852_A_G | 8.45E-06 |
| CHR5_75093974_G_A | 1.49E-05 |
| CHR5_75094076_A_C | 1.21E-05 |
| CHR5_75061650_G_A | 9.71E-05 |
| CHR17_57521416_GA_G | 0.003275 |
| CHR5_75202890_C_T | 4.7E-06 |
| CHR5_75140749_C_T | 4.17E-06 |
| CHR5_75109080_C_T | 9.3E-06 |
| CHR5_75137535_T_C | 1.21E-05 |
| CHR5_75211565_C_A | 1.06E-05 |
| CHR5_75250102_A_G | 4.56E-05 |
| CHR5_75202060_T_C | 1.07E-05 |
| CHR5_75101559_A_G | 7.67E-06 |
| CHR2_32880300_G_T | 0.00125 |
| CHR5_75200911_C_CAA | 0.00095 |
| CHR5_75627765_A_G | 0.000151 |
| CHR5_75204930_G_A | 1.33E-05 |
| CHR5_75230664_CHRG | 5.27E-06 |
| CHR5_75231814_G_A | 5.69E-06 |
| CHR5_75174938_T_A | 9.36E-06 |
| CHR5_75180131_A_C | 1.21E-05 |
| CHR5_75194173_G_A | 1.14E-05 |
| CHR5_75132517_T_A | 6.17E-06 |
| CHR6_160698546_T_C | 0.000473 |
| CHR5_75234833_G_T | 1.16E-05 |
| CHR5_75234832_G_GT | 1.93E-05 |
| CHR5_75238077_G_A | 2.87E-05 |
| CHR2_32881692_A_G | 0.001337 |
| CHR5_75212587_G_T | 1.21E-05 |
| CHR5_75136063_G_C | 8.13E-06 |
| CHR2_21055050_A_G | 0.00033 |
| CHR5_75077535_C_T | 7.88E-06 |
| CHR5_75230117_C_T | 5.07E-06 |
| CHR5_75154453_T_A | 1.32E-05 |
| CHR5_75152677_T_A | 1.85E-05 |
| CHR5_75235325_A_T | 2.07E-05 |
| CHR5_75329687_G_A | 0.00039 |
| CHR5_75130278_T_A | 7.24E-06 |
| CHR5_75131912_A_C | 6.04E-06 |
| CHR5_75156185_G_A | 1.14E-05 |
| CHR5_75131884_T_C | 3.43E-06 |
| CHR2_155110646_T_C | 0.001267 |
| CHR10_49254466_CTG_C | 0.003813 |
| CHR5_75548537_G_A | 0.000485 |
| CHR5_75132853_G_A | 7.31E-06 |
| CHR5_75126782_C_T | 1.13E-05 |
| CHR5_75152669_C_T | 8.02E-06 |
| CHR5_75122294_CA_C | 5.82E-05 |
| CHR5_75124771_G_C | 1.49E-05 |
| CHR17_6469704_A_T | 0.001764 |
| CHR5_75129950_G_A | 9.58E-06 |
| CHR19_44928869_AAAAAC_A | 0.000927 |
| CHR2_200831180_C_CA | 0.003156 |
| CHR6_31589449_G_A | 0.001256 |
| CHR5_75125572_A_G | 1.8E-05 |
| CHR5_75252239_C_T | 4.99E-05 |
| CHR5_75231647_C_T | 5.84E-06 |
| CHR5_75195890_TTCA_T | 3.31E-06 |
| CHR2_21158342_A_C | 0.000258 |
| CHR5_75115502_C_A | 4.09E-05 |
| CHR5_75202190_C_T | 1.12E-05 |
| CHR13_106199807_C_T | 0.002546 |
| CHR5_75130543_A_G | 8.41E-06 |
| CHR5_75233033_G_T | 8.71E-06 |
| CHR5_75066032_C_A | 7.34E-06 |
| CHR5_75205036_T_TTG | 2.49E-05 |
| CHR5_75252347_C_G | 6.44E-05 |
| CHR19_11118069_T_C | 0.002269 |
| CHR5_75255957_C_T | 6.32E-05 |
| CHR2_32882192_T_C | 0.001365 |
| CHR5_75135348_C_A | 1.26E-05 |
| CHR2_155114581_G_A | 0.001143 |
| CHR17_6469703_C_A | 0.001801 |
| CHR2_154952742_T_G | 0.001231 |
| CHR19_44929021_T_C | 0.000708 |
| CHR19_44892009_G_A | 0.002276 |
| CHR10_110614544_C_T | 0.00451 |
| CHR5_75130494_A_G | 2.76E-05 |
| CHR2_32881183_G_A | 0.001273 |
| CHR2_21160664_G_A | 0.000278 |
| CHR13_106201597_G_A | 0.002849 |
| CHR5_75627153_A_G | 9.8E-05 |
| CHR5_75160906_T_C | 0.00018 |
| CHR5_75379150_T_C | 0.000146 |
| CHR19_44928640_G_A | 0.000748 |
| CHR2_21017042_TAAAG_T | 0.001718 |
| CHR2_21241050_C_T | 0.000722 |
| CHR8_142751911_G_A | 0.001962 |
| CHR1_77459339_G_A | 0.003987 |
| CHR5_75531791_C_CA | 0.001808 |
| CHR2_20952995_G_A | 0.001331 |
| CHR5_75396951_A_G | 0.001526 |
| CHR5_6292343_C_T | 0.00278 |
| CHR5_75345142_C_CT | 0.000811 |
| CHR19_44928379_A_G | 0.000764 |
| CHR5_75133340_A_C | 2.85E-05 |
| CHR5_75458530_A_T | 8.64E-05 |
| CHR2_20943529_T_C | 0.002275 |
| CHR19_44929300_G_C | 0.000865 |
| CHR2_21145345_A_G | 0.000237 |
| CHR5_75253696_C_G | 4.79E-05 |
| CHR19_44895376_G_C | 0.003591 |
| CHR5_75486300_C_T | 0.000103 |
| CHR19_11119089_G_A | 0.002116 |
| CHR5_75385948_T_C | 0.000177 |
| CHR5_75332672_C_A | 0.000445 |
| CHR19_44842530_A_T | 0.001962 |
| CHR2_20994618_C_T | 0.001824 |
| CHR7_101404440_C_T | 0.002026 |
| CHR5_75551591_A_G | 0.000103 |
| CHR5_75111202_CA_C | 0.000221 |
| CHR5_75627496_G_A | 6.6E-05 |
| CHR5_75140269_A_G | 0.000265 |
| CHR6_150400629_G_A | 0.001809 |
| CHR5_75602927_T_C | 6.82E-05 |
| CHR8_142693718_TA_T | 0.00151 |
| CHR10_49253733_CTG_C | 0.003467 |
| CHR5_75544636_T_C | 8.27E-05 |
| CHR19_11119174_C_G | 0.001995 |
| CHR5_75629337_C_T | 0.00015 |
| CHR5_75626058_C_T | 0.000166 |
| CHR2_32878601_A_G | 0.001714 |
| CHR5_75550788_G_A | 8.7E-05 |
| CHR6_31565829_C_T | 0.001131 |
| CHR2_21248878_G_A | 0.000816 |
| CHR1_219392044_G_A | 0.00122 |
| CHR5_75559601_A_G | 7.19E-05 |
| CHR5_75131368_T_C | 6.86E-06 |
| CHR5_75241646_A_G | 4E-05 |
| CHR5_75629065_A_G | 7.66E-05 |
| CHR5_75251659_A_C | 6.24E-05 |
| CHR5_75383112_G_A | 0.000114 |
| CHR5_75600973_G_A | 7.61E-05 |
| CHR5_75241688_G_A | 0.00111 |
| CHR19_11096053_T_C | 0.000975 |
| CHR19_44928832_T_C | 0.000665 |
| CHR5_75534122_T_C | 0.000116 |
| CHR5_75617971_G_A | 8.34E-05 |
| CHR5_75521411_G_A | 9.27E-05 |
| CHR5_75628131_G_A | 6.18E-05 |
| CHR17_6465504_T_A | 0.001839 |
| CHR5_75626791_T_G | 0.000296 |
| CHR2_21103311_A_G | 0.000263 |
| CHR6_31553525_G_C | 0.001157 |
| CHR5_75554538_T_C | 6.49E-05 |
| CHR13_106192773_A_G | 0.002599 |
| CHR13_106191201_T_C | 0.002631 |
| CHR2_155008421_T_C | 0.001163 |
| CHR5_75627756_C_T | 0.000146 |
| CHR5_75562461_G_A | 7.63E-05 |
| CHR5_75429784_T_C | 0.000111 |
| CHR6_150397050_C_T | 0.001606 |
| CHR2_21160481_C_T | 0.002008 |
| CHR5_75521016_CT_C | 0.000179 |
| CHR5_75625861_G_A | 8.86E-05 |
| CHR6_150403445_G_A | 0.001754 |
| CHR7_101408251_T_A | 0.002101 |
| CHR2_21158305_G_A | 0.001916 |
| CHR19_11097101_C_T | 0.00109 |
| CHR5_75332189_G_C | 0.000419 |
| CHR5_75387079_A_G | 0.000156 |
| CHR10_116619563_C_CT | 0.00313 |
| CHR5_75421828_C_T | 0.000107 |
| CHR5_75385675_C_A | 0.000109 |
| CHR5_75353673_C_T | 0.000407 |
| CHR5_75623584_G_A | 0.000294 |
| CHR11_61813896_A_G | 0.003444 |
| CHR5_75206151_G_C | 8.95E-06 |
| CHR5_75059050_G_T | 0.000648 |
| CHR17_6474679_G_A | 0.001556 |
| CHR5_75626071_G_A | 6.52E-05 |
| CHR5_75533025_G_A | 9.85E-05 |
| CHR5_75563609_T_C | 8.92E-05 |
| CHR6_31551998_T_C | 0.001093 |
| CHR13_106193923_AT_A | 0.001801 |
| CHR5_75252107_T_A | 4.5E-05 |
| CHR2_21151359_G_C | 0.000948 |
| CHR2_21116413_C_A | 0.000246 |
| CHR16_54695579_T_C | 0.003011 |
| CHR6_31585691_A_G | 0.001133 |
| CHR2_21205417_A_G | 0.00045 |
| CHR5_75618236_G_A | 8.26E-05 |
| CHR5_75510279_C_T | 0.000164 |
| CHR2_21017012_A_T | 0.0017 |
| CHR16_54679956_A_G | 0.002869 |
| CHR5_75183020_G_A | 0.001006 |
| CHR5_75364531_T_C | 0.000203 |
| CHR1_56698111_C_G | 0.00502 |
| CHR6_6444484_C_A | 0.003897 |
| Sex_from_meta_manual | 0.032414 |
| age | 0.080396 |
| bmi | 0.14219 |
| pc0 | 0.022552 |
| pc1 | 0.024264 |
| pc2 | 0.023441 |
| pc3 | 0.026334 |
| pc4 | 0.023289 |
| pc5 | 0.022688 |
| pc6 | 0.024274 |
| pc7 | 0.023735 |
| pc8 | 0.024709 |
| pc9 | 0.024706 |

**Supplementary table S15.** Predictive ability assessment for total cholesterol scales from PGS Catalog.

| **Polygenic score model ID from PGS Catalog** | **Partial correlation for the entire validation set** | **Partial correlation for men** | **Partial correlation for women** |
| --- | --- | --- | --- |
| PGS000062 | -0.11297 | -0.10873 | -0.11697 |
| PGS003827 | -0.02289 | -0.1168 | 0.035789 |
| PGS003832 | -0.02047 | -0.13408 | 0.049838 |
| PGS002522 | -0.01418 | -0.13795 | 0.055275 |
| PGS002377 | 0.009666 | -0.03711 | 0.053748 |
| PGS003822 | 0.01435 | 0.102684 | -0.03954 |
| PGS004333 | 0.020638 | 0.000894 | 0.03867 |
| PGS002669 | 0.029864 | 0.060262 | -0.00679 |
| PGS002718 | 0.036003 | 0.062406 | 0.006326 |
| PGS002352 | 0.04017 | 0.057774 | 0.006963 |
| PGS002473 | 0.04066 | 0.0544 | 0.020946 |
| PGS002424 | 0.051591 | 0.08884 | 0.017853 |
| PGS003350 | 0.081235 | 0.021705 | 0.118508 |
| PGS003821 | 0.092846 | 0.036287 | 0.137271 |
| PGS002620 | 0.111214 | 0.145103 | 0.077365 |
| PGS003826 | 0.120265 | 0.081523 | 0.158487 |
| PGS003341 | 0.122254 | 0.083824 | 0.144614 |
| PGS003831 | 0.122808 | 0.176727 | 0.091624 |
| PGS003830 | 0.127711 | 0.094343 | 0.150142 |
| PGS003853 | 0.139665 | 0.163217 | 0.134105 |
| PGS003481 | 0.146188 | 0.012135 | 0.238261 |
| PGS003134 | 0.148031 | 0.064755 | 0.201973 |
| PGS003139 | 0.154618 | 0.058024 | 0.217939 |
| PGS003495 | 0.161996 | 0.04328 | 0.234543 |
| PGS004678 | 0.166905 | 0.192501 | 0.156048 |
| PGS003820 | 0.168169 | 0.128286 | 0.200648 |
| PGS000658 | 0.1715 | 0.11232 | 0.213701 |
| PGS004668 | 0.175159 | 0.1175 | 0.227781 |
| PGS003819 | 0.179244 | 0.100717 | 0.225829 |
| PGS004677 | 0.180577 | 0.192748 | 0.17867 |
| PGS003824 | 0.180634 | 0.141697 | 0.206382 |
| PGS004667 | 0.186315 | 0.166341 | 0.211073 |
| PGS000831 | 0.188924 | 0.137839 | 0.23866 |
| PGS003141 | 0.189513 | 0.077462 | 0.258691 |
| PGS003136 | 0.191212 | 0.090635 | 0.2539 |
| PGS003137 | 0.194046 | 0.089335 | 0.261961 |
| PGS003135 | 0.198675 | 0.127553 | 0.242374 |
| PGS003140 | 0.199889 | 0.105584 | 0.262955 |
| PGS003142 | 0.203216 | 0.101502 | 0.270011 |
| PGS001895 | 0.205592 | 0.097597 | 0.275788 |
| PGS003143 | 0.206089 | 0.184028 | 0.219574 |
| PGS004673 | 0.206102 | 0.219487 | 0.20341 |
| PGS003138 | 0.212728 | 0.188538 | 0.229508 |
| PGS003825 | 0.214216 | 0.143329 | 0.264489 |
| PGS002108 | 0.216451 | 0.124449 | 0.276098 |
| PGS000311 | 0.222376 | 0.131629 | 0.288371 |
| PGS004670 | 0.227779 | 0.201689 | 0.255044 |
| PGS004671 | 0.228518 | 0.183726 | 0.269315 |
| PGS000677 | 0.23358 | 0.114979 | 0.305217 |
| PGS002571 | 0.236804 | 0.147502 | 0.295546 |
| PGS003823 | 0.238579 | 0.189866 | 0.277578 |
| PGS003829 | 0.243014 | 0.218153 | 0.259985 |
| PGS004672 | 0.246029 | 0.236607 | 0.261357 |
| PGS004674 | 0.248229 | 0.200925 | 0.290355 |
| PGS004669 | 0.250421 | 0.192599 | 0.293513 |
| PGS002783 | 0.255253 | 0.226624 | 0.282273 |
| PGS004841 | 0.255489 | 0.159025 | 0.319326 |
| PGS004679 | 0.256825 | 0.20308 | 0.293724 |
| PGS004676 | 0.265014 | 0.238852 | 0.290097 |
| PGS004681 | 0.266924 | 0.232042 | 0.295731 |
| PGS004844 | 0.269431 | 0.174818 | 0.337911 |
| PGS003828 | 0.269639 | 0.243727 | 0.290457 |
| PGS004842 | 0.271517 | 0.177704 | 0.340595 |
| PGS004680 | 0.275428 | 0.230698 | 0.310928 |
| PGS003834 | 0.276244 | 0.25043 | 0.293847 |
| PGS003818 | 0.277754 | 0.208887 | 0.32459 |
| PGS004675 | 0.286665 | 0.260281 | 0.312272 |
| PGS004843 | 0.297425 | 0.227873 | 0.348549 |
| PGS003833 | 0.297566 | 0.238901 | 0.344997 |

**Supplementary table S16.** Predictive ability assessment for HDL-cholesterol scales from PGS Catalog.

| **Polygenic score model ID from PGS Catalog** | **Partial correlation for the entire validation set** |
| --- | --- |
| PGS000309 | -0.06733 |
| PGS004140 | -0.05062 |
| PGS002499 | -0.0459 |
| PGS004914 | -0.04079 |
| PGS004631 | -0.03941 |
| PGS002548 | -0.03117 |
| PGS004636 | -0.02641 |
| PGS000686 | -0.0201 |
| PGS002450 | -0.01956 |
| PGS003781 | -0.01891 |
| PGS004633 | -0.01645 |
| PGS000064 | -0.01633 |
| PGS003779 | -0.0152 |
| PGS003776 | -0.01519 |
| PGS002329 | -0.01424 |
| PGS004628 | -0.01383 |
| PGS004632 | -0.01289 |
| PGS003877 | -0.01182 |
| PGS002597 | -0.01166 |
| PGS004627 | -0.01067 |
| PGS002695 | -0.0094 |
| PGS002401 | -0.00932 |
| PGS004630 | -0.00875 |
| PGS003771 | -0.00579 |
| PGS004110 | -0.00559 |
| PGS003878 | -0.00407 |
| PGS004002 | -0.00223 |
| PGS003338 | -0.00163 |
| PGS004126 | 0.001693 |
| PGS000845 | 0.002771 |
| PGS002954 | 0.004717 |
| PGS003880 | 0.004848 |
| PGS004635 | 0.006295 |
| PGS003778 | 0.007551 |
| PGS002959 | 0.010379 |
| PGS003769 | 0.010618 |
| PGS003768 | 0.010967 |
| PGS004056 | 0.013175 |
| PGS002781 | 0.014127 |
| PGS003856 | 0.016434 |
| PGS004086 | 0.01845 |
| PGS004043 | 0.019032 |
| PGS004028 | 0.021441 |
| PGS002646 | 0.021451 |
| PGS003347 | 0.021766 |
| PGS004626 | 0.023777 |
| PGS004777 | 0.026494 |
| PGS000660 | 0.027626 |
| PGS004100 | 0.028029 |
| PGS000825 | 0.028635 |
| PGS004776 | 0.029273 |
| PGS004778 | 0.029273 |
| PGS002962 | 0.029884 |
| PGS003767 | 0.030494 |
| PGS004072 | 0.031248 |
| PGS003879 | 0.031538 |
| PGS003774 | 0.031953 |
| PGS002957 | 0.032632 |
| PGS004623 | 0.033355 |
| PGS004932 | 0.03365 |
| PGS004634 | 0.034448 |
| PGS000192 | 0.037462 |
| PGS003876 | 0.037735 |
| PGS003777 | 0.03805 |
| PGS004629 | 0.038337 |
| PGS001954 | 0.038348 |
| PGS004156 | 0.038489 |
| PGS004625 | 0.041593 |
| PGS004622 | 0.047466 |
| PGS002958 | 0.047602 |
| PGS002172 | 0.048678 |
| PGS003782 | 0.049299 |
| PGS003775 | 0.049831 |
| PGS003986 | 0.049922 |
| PGS003783 | 0.050354 |
| PGS002366 | 0.051533 |
| PGS002956 | 0.054001 |
| PGS003534 | 0.059598 |
| PGS002961 | 0.060105 |
| PGS003772 | 0.061226 |
| PGS002963 | 0.062858 |
| PGS002960 | 0.063677 |
| PGS003875 | 0.064799 |
| PGS004775 | 0.067986 |
| PGS003773 | 0.068839 |
| PGS004624 | 0.069422 |
| PGS002955 | 0.069936 |
| PGS003780 | 0.071424 |
| PGS003770 | 0.084739 |
| PGS000060 | 0.087206 |

**Supplementary table S17.** Predictive ability assessment for LDL-cholesterol scales from PGS Catalog.

| **Polygenic score model ID from PGS Catalog** | **Partial correlation for the entire validation set** |
| --- | --- |
| PGS000061 | -0.12122 |
| PGS002507 | -0.0544 |
| PGS003977 | -0.04121 |
| PGS003793 | -0.01765 |
| PGS003798 | -0.01724 |
| PGS000896 | 0.001114 |
| PGS003788 | 0.007471 |
| PGS003339 | 0.011021 |
| PGS003348 | 0.011105 |
| PGS002730 | 0.023188 |
| PGS003871 | 0.024344 |
| PGS002654 | 0.025255 |
| PGS003975 | 0.034902 |
| PGS000192 | 0.037462 |
| PGS003974 | 0.040955 |
| PGS002703 | 0.043739 |
| PGS004936 | 0.045165 |
| PGS002337 | 0.045693 |
| PGS003787 | 0.052021 |
| PGS002458 | 0.054952 |
| PGS002409 | 0.058206 |
| PGS004975 | 0.058957 |
| PGS000661 | 0.061353 |
| PGS002369 | 0.061842 |
| PGS003796 | 0.065195 |
| PGS000897 | 0.069183 |
| PGS000890 | 0.070531 |
| PGS000891 | 0.072618 |
| PGS003976 | 0.073561 |
| PGS000340 | 0.076882 |
| PGS003403 | 0.076882 |
| PGS003978 | 0.082025 |
| PGS004969 | 0.084263 |
| PGS003797 | 0.085737 |
| PGS003792 | 0.088294 |
| PGS004647 | 0.09325 |
| PGS004970 | 0.098691 |
| PGS000886 | 0.099852 |
| PGS003874 | 0.104266 |
| PGS003791 | 0.104899 |
| PGS004648 | 0.108304 |
| PGS000814 | 0.10899 |
| PGS004643 | 0.109415 |
| PGS003517 | 0.111149 |
| PGS000894 | 0.113116 |
| PGS003404 | 0.113459 |
| PGS003872 | 0.119176 |
| PGS003869 | 0.120305 |
| PGS000875 | 0.121826 |
| PGS004974 | 0.122201 |
| PGS000895 | 0.123483 |
| PGS004977 | 0.124842 |
| PGS004638 | 0.12617 |
| PGS003785 | 0.131487 |
| PGS004637 | 0.132407 |
| PGS003855 | 0.135522 |
| PGS004976 | 0.137271 |
| PGS003786 | 0.138327 |
| PGS004979 | 0.140047 |
| PGS004971 | 0.142053 |
| PGS004641 | 0.144216 |
| PGS003870 | 0.146006 |
| PGS004973 | 0.147078 |
| PGS003029 | 0.14798 |
| PGS003405 | 0.149838 |
| PGS000887 | 0.14985 |
| PGS003474 | 0.151149 |
| PGS004980 | 0.153378 |
| PGS003790 | 0.153575 |
| PGS003034 | 0.154818 |
| PGS000065 | 0.155803 |
| PGS003789 | 0.157575 |
| PGS000824 | 0.160835 |
| PGS004972 | 0.160842 |
| PGS004981 | 0.161281 |
| PGS004646 | 0.169283 |
| PGS003795 | 0.169675 |
| PGS004978 | 0.170361 |
| PGS004640 | 0.175422 |
| PGS004639 | 0.175887 |
| PGS000846 | 0.179205 |
| PGS000889 | 0.182544 |
| PGS000893 | 0.183218 |
| PGS000888 | 0.183452 |
| PGS003030 | 0.183921 |
| PGS003035 | 0.184841 |
| PGS003036 | 0.187819 |
| PGS002605 | 0.18937 |
| PGS004651 | 0.189725 |
| PGS004915 | 0.190501 |
| PGS000892 | 0.190519 |
| PGS000115 | 0.190681 |
| PGS003031 | 0.191318 |
| PGS004642 | 0.193378 |
| PGS004644 | 0.195856 |
| PGS001933 | 0.196295 |
| PGS003038 | 0.19666 |
| PGS002556 | 0.197589 |
| PGS003032 | 0.197833 |
| PGS002150 | 0.198357 |
| PGS004649 | 0.19868 |
| PGS004650 | 0.19925 |
| PGS003873 | 0.200734 |
| PGS000310 | 0.200963 |
| PGS003033 | 0.211864 |
| PGS003794 | 0.212828 |
| PGS004645 | 0.21914 |
| PGS003800 | 0.219567 |
| PGS004794 | 0.222506 |
| PGS003037 | 0.22293 |
| PGS000688 | 0.225603 |
| PGS004792 | 0.2275 |
| PGS003799 | 0.238204 |
| PGS003784 | 0.252851 |
| PGS002274 | 0.26063 |
| PGS004793 | 0.264183 |
| PGS004791 | 0.266756 |
